# Genome-wide DNA methylation encodes cardiac transcriptional reprogramming in human ischemic heart failure

**DOI:** 10.1038/s41374-018-0104-x

**Published:** 2018-08-08

**Authors:** Mark E. Pepin, Chae-Myeong Ha, David K. Crossman, Silvio H. Litovsky, Sooryanarayana Varambally, Joseph P. Barchue, Salpy V. Pamboukian, Nikolaos A. Diakos, Stavros G. Drakos, Steven M. Pogwizd, Adam R. Wende

**Affiliations:** 1 Dept of Pathology, Div of Molecular and Cellular Pathology, University of Alabama at Birmingham, Birmingham, AL 35294 USA; 2 Dept of Biomedical Engineering, University of Alabama at Birmingham, Birmingham, AL 35294 USA; 3 Dept of Genetics, Heflin Center for Genomic Science, University of Alabama at Birmingham, Birmingham, AL 35294 USA; 4 Dept of Pathology, Div of Anatomic Pathology, University of Alabama at Birmingham, Birmingham, AL 35294 USA; 5 Dept of Medicine, Div of Cardiovascular Medicine, University of Alabama at Birmingham, Birmingham, AL 35294 USA; 6 Dept of Internal Medicine, Div of Cardiovascular Medicine & Nora Eccles Harrison Cardiovascular Research and Training Institute (CVRTI), University of Utah, Salt Lake City, UT 84108 USA

**Keywords:** Transcriptomics, Heart failure, Diagnostic markers, Mechanisms of disease, DNA methylation

## Abstract

Ischemic cardiomyopathy (ICM) is the clinical endpoint of coronary heart disease and a leading cause of heart failure. Despite growing demands to develop personalized approaches to treat ICM, progress is limited by inadequate knowledge of its pathogenesis. Since epigenetics has been implicated in the development of other chronic diseases, the current study was designed to determine whether transcriptional and/or epigenetic changes are sufficient to distinguish ICM from other etiologies of heart failure. Specifically, we hypothesize that genome-wide DNA methylation encodes transcriptional reprogramming in ICM. RNA-sequencing analysis was performed on human ischemic left ventricular tissue obtained from patients with end-stage heart failure, which enriched known targets of the polycomb methyltransferase EZH2 compared to non-ischemic hearts. Combined RNA sequencing and genome-wide DNA methylation analysis revealed a robust gene expression pattern consistent with suppression of oxidative metabolism, induced anaerobic glycolysis, and altered cellular remodeling. Lastly, KLF15 was identified as a putative upstream regulator of metabolic gene expression that was itself regulated by EZH2 in a SET domain-dependent manner. Our observations therefore define a novel role of DNA methylation in the metabolic reprogramming of ICM. Furthermore, we identify EZH2 as an epigenetic regulator of KLF15 along with DNA hypermethylation, and we propose a novel mechanism through which coronary heart disease reprograms the expression of both intermediate enzymes and upstream regulators of cardiac metabolism such as KLF15.

## Abstract (web summary)

Human ischemic cardiomyopathy is defined by DNA hypermethylation, methyltransferase EZH2 induction, and transcription factor KLF15 suppression. Together these changes may mediate a gene expression pattern reflecting decreased oxidative phosphorylation and increased cellular remodeling. This study therefore identifies a novel mechanism through which coronary heart disease may be regulated.

## Introduction

Coronary heart disease (CHD) is the leading cause of death and disability in developed countries. Although the successes of fibrinolytic and non-invasive therapies have markedly improved survival post-myocardial infarction, cardiac function often declines until patients develop symptoms of congestive heart failure, a terminal outcome known as ischemic cardiomyopathy (ICM). Despite its growing prevalence, the current medical approach to ICM is limited to symptomatic management, in part due to an inadequate understanding of the distinct etiologic mechanisms that describe the clinical syndrome [1]. Effectiveness of even symptomatic management varies widely among patients, suggesting that both pathogenesis and optimal treatment of ICM require precision-based medical approaches [2, 3].

Contrary to prior belief, it is now well-recognized that up to 40% of the myocardial segments affected by coronary artery stenosis remain viable [4, 5]. This ischemic myocardium adapts to chronic hypoperfusion by suppressing myofibrillar protein expression and reducing its energetic demand, a process termed colliquative myocytolysis [6]. These morphologic changes are accompanied by metabolic adaptation of cardiac tissue to a “fetal-like” state, wherein the heart preferentially consumes glucose over fatty acids for its energy supply [7–9]. Although hypoxia and nutrient availability contribute to the reactivation of this transcriptional program, as is the case during its in utero development, the mechanisms through which cardiac nutrient-sensing produces global changes in metabolic gene expression remain poorly understood [10, 11].

Epigenetics is a regulatory mechanism whereby fluctuations in the cellular microenvironment reprogram gene expression. As a field, epigenetics describes a number of stable yet reversible modifications to genomic structure. While epigenetics was originally used to explain the establishment of cellular identity during embryogenesis, the reverse process occurs in many adult diseases such as cancer [12]. Interestingly, global fluctuations in DNA methylation have been recognized as both a cause and consequence of metabolic plasticity [13, 14].

Therefore, owing to the analogous metabolic changes that occur in the failing ischemic heart, the current study examines genome-wide cardiac methylation of patients with end-stage heart failure to determine whether epigenetic reprogramming also exists in ICM. Our findings establish a novel relationship between DNA methylation and gene expression of metabolic pathway intermediates in cardiac tissue. Furthermore, we identify EZH2 and KLF15 as putative regulators of ICM-associated epigenetic reprogramming. We propose that EZH2 contributes to this well-known shift in metabolic substrate preference, offering a new molecular target of mechanistic study in the pursuit of precision-based cardiac therapies.

## Materials and methods

### Human left ventricular cardiac samples

**Fig. 1:**
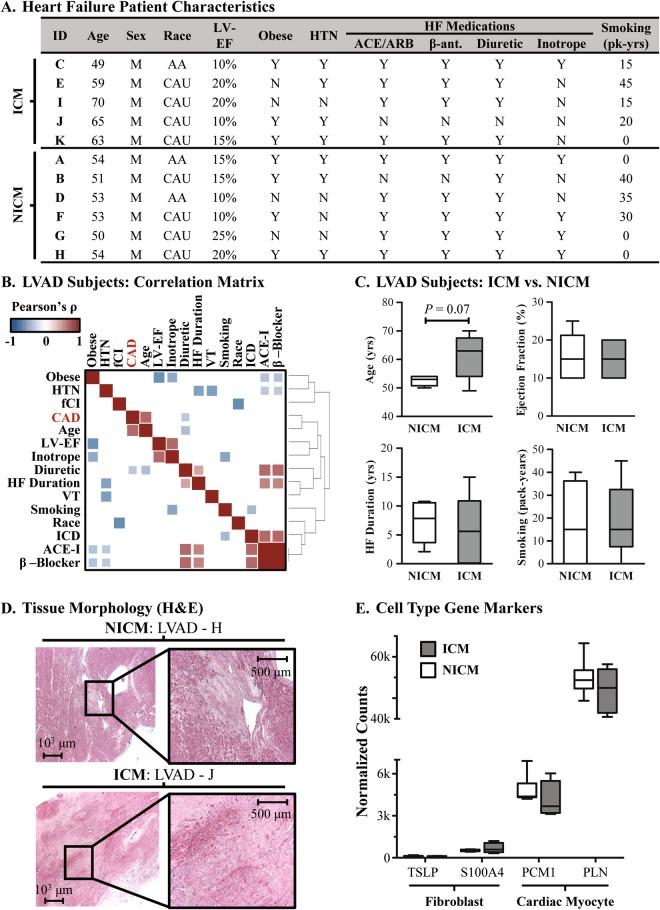
Correlation of pre-LVAD patient characteristics. **a** Table outlining patient characteristics collected from electronic medical records. **b** Correlation matrix and hierarchical clustering based on Pearson’s coefficient performed on patient metrics, displaying only interrelations reaching statistical significance at *P* = 0.05 level. All Boolean (i.e. “Yes” or “No”) variables were converted to binary (“1” or “0”, respectively) prior to analysis; race was converted to a limited factor of African American (“1”) and Caucasian (“0”). **c** Distributions of patient age (yrs), ejection fraction (%), duration of heart failure (yrs), and history of smoking (pack-years) were compared between ICM and NICM. **d** Representative H&E-stained sections of myocardial samples used in the sequencing analysis, showing Subject LVAD-H (NICM) and LVAD-J (ICM). **e** RNA expression of cardiomyocyte nucleic markers PCM1 and PLN as normalized counts from RNA-sequencing analysis; n.s. = non-significant

Adult heart failure patients admitted to the University of Alabama at Birmingham University Hospital for LVAD implantation due to end-stage heart failure were considered for the study. Because segmental influences of ischemic events have documented transcriptional effects [15, 16], the consistency of our tissue collection protocol was of highest priority. Only cardiac tissue located in the apex of the left ventricle was used from patients with or without prior history of obstructive coronary artery disease (CAD). Male patients between 49 and 70 years of age were selected. All HF patients were end-stage (NYHA Class IV) and exhibited severe systolic dysfunction, as assessed by reduced ejection fraction (Fig. 1a). Left ventricular samples from patients who passed the selection process were classified as either ICM or NICM based on presence or absence of significant coronary artery stenosis (>80%), as determined from left heart catheterization prior to LVAD implantation.

### RNA sequencing analysis

RNA isolation was performed using the RNeasy® Fibrous Tissue Mini Kit (Qiagen Inc., Valencia, CA), following the manufacture’s protocol. Extracted RNA was analyzed to ensure RNA quality, with RNA Integrity Numbers (RINs) >7. High-throughput mRNA-sequencing was performed using Illumina HiSeq2000, providing up to 300 Gb of sequence output per flow cell. The quality of total RNA was assessed using the Agilent 2100 Bioanalyzer, followed by 2 rounds of poly A+ selection and conversion to cDNA. The TruSeq library generation kits were used per the manufacturer’s instructions (Illumina, San Diego, CA). Library construction consisted of random fragmentation of the polyA mRNA, followed by cDNA production using random primers. The ends of the cDNA were repaired, with A-tails and adapters ligated for indexing (up to 12 different barcodes per lane) during the sequencing runs. The cDNA libraries were quantified using qPCR in a Roche LightCycler 480 with the Kapa Biosystems kit for library quantitation (Kapa Biosystems, Woburn, MA) prior to cluster generation. Clusters were generated to yield approximately 725K-825K clusters/mm^2^. Cluster density and quality were determined during the run after the first base addition parameters were assessed. A series paired-end 2 × 100 bp sequencing runs was performed to align the cDNA sequences to the reference genome.

TopHat (version 2.0.12) was used to align RNA-Seq reads to the reference genome (hg19) using short-read aligner Bowtie2 (version 2.2.3). Cufflinks (version 2.2.1) was used to assemble transcripts from the aligned reads, estimate their abundances and test for differential expression. Cuffcompare (version 2.2.1), a component of Cufflinks, related the assembled transcripts to a reference annotation and tracked Cufflinks transcripts across multiple experiments. Finally, Cuffdiff (version 2.2.1) identified significant changes in transcript expression.

### DNA methylation array

Genome-wide differential DNA methylation was performed on remaining tissue from the same excised tissue that was used for RNA sequencing. DNA was extracted using the DNeasy kit (Qiagen Inc., Valencia, CA). The Illumina Infinium Human Methylation450 Beadchip array was then used to interrogate 485,577 Cytosine-p-Guanine (CpG) sites, which covers >99% of the RefSeq genes with an average of 17 CpG sites per reference gene, and distributed across the gene promoter, 5′ UTR, body, and 3′ UTR. For each assay, 500 ng DNA was bisulfite treated with the EZ DNA Methylation Kit (Zymo, Irvine, CA) before amplification, hybridization, and imaging standard to the Illumina protocol.

The R package minfi (version 1.15.11), was used to analyze the raw idat files from the Illumina iScan to identify significant CpG site changes. Samples were preprocessed and normalized using subset-quantile within array normalization (SWAN) which corrected for the technical differences between the Type I and Type II array designs [17]. Probes that contained a SNP at either the CpG interrogation or at the single nucleotide extension were removed from all downstream analysis. The methylation data were then filtered using significance thresholds consistent with current literature, with statistical significance based on a *P*-value <0.05. Statistical analysis was performed using multiple t-tests with ICM vs. NICM as the primary comparison, controlling for other available patient variables. Biological relevance was estimated by |methylation change| ≥5%.

### Cell culture and transient transfection

The H9c2 cells (rat cardiomyoblasts) were cultured in 10% fetal bovine serum (Gibco, Grand Island, NY, USA) containing DMEM (Gibco), 5 mM Glucose, and 1× penicillin and streptomycin (Sigma-Aldrich, St. Louis, MO, USA), in a humidified atmosphere with 5% CO_2_ at 37 °C. Plasmids expressing constitutively active human myc-tagged EZH2 (pcDNA3.1-EZH2), ΔSET-EZH2 (pcDNA3.1-ΔSET-EZH2), and empty vector (pcDNA3.1-EMPTY) were used as previously described [18]. For transient expression of pcDNA3.1-EZH2 or pcDNA3.1-ΔSET-EZH2, H9c2 cells were transfected with Lipofectamine^®^ 3000 (Invitrogen, Carlsbad, CA, USA) according to the manufacturer’s instructions.

### Quantitative real-time PCR

Total RNA from cell cultures of H9c2 rat cardiomyoblasts was isolated using TRIzol™ reagent (Invitrogen), as described previously [19]. cDNA was synthesized by reverse transcription PCR using SuperScript III, DNase I treatment and anti-RNase treatment (Invitrogen). Differential gene expression of *Ezh2* (forward:5′-CCCTGACCTCTGTCTTACTTGTGGA-3′; reverse:5′-ACGTCAGATGGTGCCAGCAATA-3′) and Klf15 (forward: 5′-GCCAAGTTCAGCCGCCA-3′; reverse: 5′-CACCATAGCAGGAGCAGAGG-3′) were standardized and normalized to reference gene β-actin (forward:5′-GCCTTCCTTCTTGGGTATGG-3′; reverse:5′-GTGCTAGGAGCCAGAGCAGT-3′).

### Western blotting

Protein expression was determined via Western blotting, as described previously [20]. Briefly, total protein lysates were prepared from H9c2 rat cardiomyoblasts. Lysates (10–20 μg) were resolved by 12% SDS-PAGE and electro-transferred onto polyvinylidene difluoride membranes (Bio-Rad, Hercules, CA, USA). Protein detection was performed with Alexa Fluor^®^ 680 (Thermo Fisher, Cat. #A20172) or IRDye^®^ 800 (LI-COR Bioscience, Lincoln, NE, USA) secondary antibodies and fluorescence was quantified using the LI-COR Odyssey Imager (LI-COR Biosciences). In order to minimize the contribution that position on the gel might have on outcomes, samples were randomized on each gel as pairs and normalized with β-tubulin (Applied Biological Materials, Richmond, BC, Canada, Cat. #G098, Mouse) to control for loading error. All bands for representative images for an individual experiment were from the same gel. EZH2 (Cell Signaling Technology, Danvers, MA, USA, Cat. #5246, Rabbit) and KLF15 (Thermo Fisher, Waltham, MA, USA, Cat. #PA5-18056, Goat) antibodies were used in the current study.

### Statistical and bioinformatics analysis

A detailed bioinformatics protocol is included as supplemental information, along with session information and input data (see Expanded Methods). Briefly, R software, (version 3.2.1, R Foundation for Statistical Computing, Vienna, Austria), was used to compute statistics and perform data visualization. Two-way comparisons were made using ischemic status as the primary variable. To test for confounding patient variables, Pearson’s correlation and linear multiple regression analyses were performed (Fig. 1b). For all targets, an unpaired student’s *t*-test was performed using Tukey’s correction for multiple comparisons. Statistical significance was concluded based on a false discovery-corrected *P*-value less than 0.05. Functional and network GSEA, along with curated literature-supported candidate upstream regulators, were performed using QIAGEN’s Ingenuity Pathway Analysis (IPA®, QIAGEN Redwood City) unless otherwise specified.

The study initially contained 6 ICM and 6 NICM subjects, but an ICM sample was identified as an extreme outlier in both DNA methylation and RNA sequencing analyses (Fig. S1); this concern was reinforced in our experimental records, which described visible sample contamination during pre-processing. Therefore, the current study considered 5 ICM and 6 NICM samples. Gene set enrichment analysis and ranking of putative upstream regulators was accomplished using *Enrichr*, a web-based interactive tool [21]. Heat map generation and hierarchical clustering were performed using *pheatmap* package (1.0.8) within R. Unless otherwise specified, hierarchical clustering was assembled using Ward’s minimum squared variance algorithm, and dendrograms were constructed by Euclidean distance.

## Results

### Patient characteristics

To ensure that our analysis of ischemic cardiomyopathy (ICM) vs. non-ischemic heart failure (NICM) was not confounded by other patient metrics, we collected a wide array of patient demographics and comorbidities (Fig. 1a). Inter-variable correlations were quantified using a correlation matrix via Pearson’s correlation, followed by clustering of variables (Fig. 1b). Patient age was found to correlate with ischemic classification; however, when we compared the group statistics of all numeric variables, only a trending difference was found with age in the selected samples between ICM and NICM (*P* = 0.07), this being largely due to the low variance of patient age in the NICM group (Fig. 1c). These observations indicate that the analysis of ICM vs. NICM was not significantly confounded by any other available patient health metrics.

### Histologic and transcriptional analysis for cellular heterogeneity

Because tissue and cellular composition are shown to influence differential gene expression within RNA sequencing data [22], we inspected the biopsies in a blinded fashion based on histopathologic evidence of ischemia. All samples were consistently identified as “ischemic” or “non-ischemic” based on histologic examination (Fig. 1d and Fig. S2), matching our prior classification based on clinical parameters. This histology also revealed that the fibrotic portions of tissue were largely acellular. Furthermore, we observed widespread dysmorphic cardiomyocytes which lacked myofibrillar striations; though seen in both tissue groups, this colliquative myocytolysis was more evident in ICM sections and was adjacent to the largely acellular foci of dense collagen in patients with ischemic cardiomyopathy.

To determine whether morphologic or cellular differences likely confer transcriptional changes, unsupervised transcriptional deconvolution was performed via principal component analysis (PCA) and hierarchical clustering with heatmap visualization. While the PCA showed lack of group separation within the first 3 principal components, heatmap revealed marked divergence between ICM and NICM at the *P* < 0.05 level (Fig. S3A and S3B). We then used *xCell* [22], a supervised machine-learning model developed for cell-type deconvolution, which we used to compare our whole-tissue RNA-sequencing data to that of isolated stromal and immune cells (Supplemental Fig. S3C). This approach elicited no significant differences in our data between ICM and NICM biopsies. Finally, we inspected the following established cell-type specific gene markers to verify similar representation of fibroblasts (TSLP, S100A4), cardiomyocytes (PCM1, PLN) (Fig. 1e). Fibroblast and cardiomyocyte gene markers were unchanged, and cardiomyocyte markers were expressed at a much higher level. Together, these observations suggest that any gene expression changes are due to differential regulation within a globally unchanging tissue composition. However, this does not rule out the need to do cardiomyocyte studies in the future to confirm the exact cellular location of our observed changes.

### Putative upstream regulator EZH2 in ischemic cardiomyopathy

**Fig. 2:**
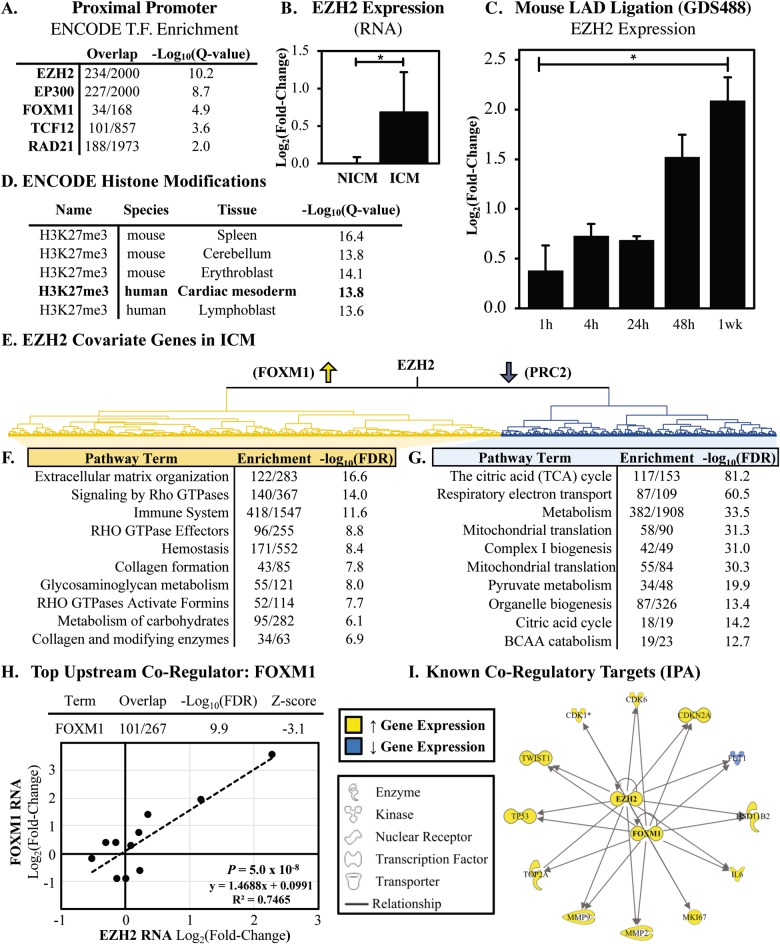
RNA sequencing analysis for ischemic heart failure comparison. **a** Empirical enrichment analysis comparing DEGs to ENCODE consortium ChIP-sequencing datasets. **b** Expression of EZH2 in Pre-LVAD left ventricular myocardium. **c** Time-course myocardial infarction microarray via LAD ligation in mouse, as published by Tarnaski et al. (PMID: 14679301, GDS488). All bar graphs illustrate the expression mean (± SD). **d** ENCODE 2015 database was used for enrichment of DEGs based on ChIP-sequencing of histone modifications. **e** Genes were selected by degree of covariation with EZH2 gene expression via Pearson’s correlation, and segregated by hierarchical clustering. Positively-correlating (**f**) and inversely-correlated genes (**g**) with EZH2 were subjected to pathway analysis using the Reactome database, reporting the enrichment ratio (# DEGs/Total Genes in dataset) and FDR-adjusted *P*-value computed by Fisher exact test. **h** Positively-correlating genes (*P* < 0.05) were inspected within the *ChIP-Enrichment Analysis* (ChEA) 2016 database to identify a putative upstream co-regulator with EZH2. **i**. IPA® was used to visualize the expression of EZH2-FOXM1 heterodimer and shared downstream targets in ICM relative to NICM (*P* < 0.05)

To identify candidate transcriptional regulators in ICM, we performed gene set enrichment against the ChIP-sequencing database developed by the ENCyclopedia of DNA Elements (ENCODE) Consortium [23]. This approach identified polycomb methyltransferase enhancer of zeste 2 polycomb repressive complex 2 subunit (EZH2) as by-far the most enriched transcriptional regulator (FDR = 10^−10.2^) (Fig. 2a, Table S1). Differential expression of EZH2 revealed a significant increase in its expression (2.0-fold, *P* = 0.008), further supporting its potential relevance as a differential regulator in ICM relative to NICM (Fig. 2b). To validate our observations of EZH2 induction in ICM, we interrogated a previously published microarray dataset (GDS488) by Tarnavski et al. detailing a time-course analysis of mouse left anterior descending coronary artery ligation [24]. We found a graded and persistent EZH2 induction in the ischemic hearts when compared to non-ischemic control hearts (Fig. 2c, Table S2). Since EZH2 is an enzyme that catalyzes the tri-methylation of histone-3 at lysine-27 (H3K27me3), we used the ENCODE histone modifications database to identify enriched histone modifications within DEG proximal promoters (Fig. 2d). Regardless of tissue-type or species, H3K27me3 was the most enriched histone modification (FDR = 10^−13.8^ for human cardiac mesoderm). Together, these observations implicate EZH2 as an epigenetic regulator of differential gene expression in ICM.

To identify genes and pathways most likely associated with EZH2 expression, all genes were ranked by Pearson’s correlation according with EZH2 expression (*P* < 0.05) (Fig. 2e). Because EZH2 is both an activator and repressor of gene expression as histone methyltransferase and heterodimer, respectively, pathway enrichment was performed separately on genes that were positively and negatively correlated with EZH2. This approach revealed a striking consistency with the established dual role of EZH2 (Fig. 2f, g). Of the genes positively correlated with EZH2, “extracellular matrix organization” was the most upregulated, and “glycolytic metabolism” (95 genes, FDR = 10^−6.1^) was found among the most enriched pathways (Fig. 2f). In contrast, pathways enriched by negatively-correlated genes were associated with oxidative metabolism. Furthermore, analysis of positively correlating genes for putative upstream regulators identified EZH2 binding partner FOXM1 as most-enriched (*P* < 10^−9.9^), which we also found to be significantly increased (4.0-fold, *P* < 0.05) (Fig. 2h, i). Inspection of downstream co-regulated targets of EZH2-FOXM1 revealed robust increases in 10/11 genes: MMP2 (1.5-fold, *P* = 0.01), MMP9 (4.9-fold, *P* *=* 5 × 10^−5^) TOP2A (7.0-fold, 5 × 10^−5^), TP53 (1.42-fold, *P* = 0.03), TWIST1 (1.9-fold, *P* = 0.004), CDK1 (3.7-fold, *P* = 0.0001), CDK6 (1.4-fold, *P* = 0.03), CDKN2A (2.8-fold, *P* = 0.006), HSD11B2 (3.3-fold, *P* = 0.001), IL6 (4.4-fold, *P* = 5 × 10^−5^), MKI67 (7.0-fold, *P* = 5 × 10^−5^). Of these, IL6, MMP2, and MMP9 are established risk correlates and mediators of adverse myocardial remodeling in heart failure [25, 26].

### Differential DNA methylation stratifies ischemic from non-ischemic cardiomyopathy

**Fig. 3:**
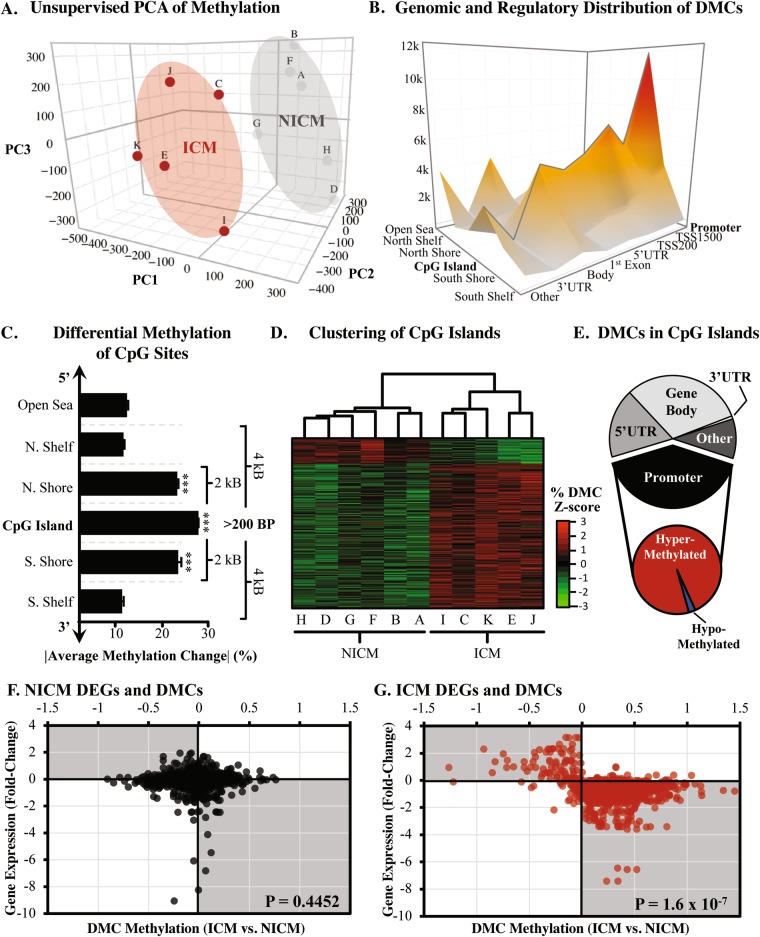
Genome-wide DNA methylation in ischemic heart failure. **a** Unsupervised two-dimensional principal component analysis using normalized beta values of the Methyl450 array. **b** 3-dimensional contour plot depicting the distribution of DMCs about CpG and genomic regions. **c** Differential methylation patterns within annotated CpG regions based on average absolute percent change in methylation (± SEM). **d** Hierarchical clustering via Wald.D2 Test and dendrogram constructed by Euclidean distance with heatmap visualization of DMCs. **e** Pie chart illustrating the distribution of CGI-associated differential methylation annotating to the proximal (1.5 kB) gene promoter (11,946 CpGs), gene body (9211 CpGs), 5′UTR (5244 CpGs), 3′UTR (228 CpGs), and non-annotated “Other” (3002 CpGs). Expanded view of Promoter-associated CGI with differential methylation are 97.5% hypermethylated (10,474 CpGs). **f** DEGs containing promoter-associated inversely changing DMCs were plotted by left ventricle samples obtained from either non-ischemic cardiomyopathy(NICM) or **g** ischemic cardiomyopathy (ICM) patients. Linear regression analysis was performed based on a Pearson’s correlation of the group, with ICM and not NICM displaying robust statistical correlation. *P* < 0.05, |Fold-Change| > 1.5, FPKM > 2. *P* < 0.05, |Methylation Change| > 5%)

While its heterodimeric interaction with FOXM1 is consistent with its role as a positive regulator [27], EZH2 is classically a polycomb group (PRC2) methyltransferase with high binding affinity for CpG-rich methylated regions of the genome [28, 29]. To examine this aspect of its function and/or recruitment, we analyzed genome-wide DNA methylation of the same human cardiac tissue used for RNA-sequencing. Samples were analyzed via Illumina Beadchip Methylation450 (Methyl450) array, a DNA methylation array covering 485,577 CpG sites (DMCs). Of these sites, 12.6% (61,233 DMCs) were differentially methylated (5% change, *P* < 0.05) between ICM and NICM. Unsupervised principal components analysis (PCA) revealed a clear separation by ischemic etiology (Fig. 3a). Interestingly, such high resolution was not attained by PCA of RNA sequencing data (Fig. S3B), highlighting the capability of the cardiac methylome but not transcriptome to distinguish between the two heart failure etiologies.

### Hypermethylation within promoter-associated CGI

To better understand the potential role of DNA methylation as a co-regulatory mechanism of cardiac metabolism with EZH2, we examined the distribution of changes in methylation across the genome. Because cytosine DNA methylation is known to confer site-specific effects on transcriptional activity [30, 31], DMCs (*P* < 0.05) were mapped onto both annotated gene regions (promoter, 5′UTR, gene body, and 3′UTR) as well as according to their distance from established CpG Islands (CGIs). This dual annotation was then used to evaluate the number of differentially-methylated CpG sites via 3-dimensional contour plot (Fig. 3b, Table S3). Although the Methyl450k array disproportionately inspects CGIs, differential DNA methylation in ICM modestly enriched promoter-associated CGIs beyond what is interrogated by the array, determined by chi-squared analysis (*P* = 0.07) (Fig. S6). Furthermore, the absolute magnitude of differential methylation was found to be greatest within CGIs (Fig. 3c). Lastly, hierarchical clustering and heatmap visualization were performed on the promoter-associated CGIs (*P* < 0.05), which illustrated robust hypermethylation between these groups within the promoter region (Fig. 3d, e). Combined, these observations suggest that ICM correlates with a relative hypermethylation of promoter-associated CGIs, consistent with the hypothesized recruitment of PRC2 to stabilize gene suppression.

### Inversely associated promoter-CpG methylation and gene expression

Because we analyzed whole-exome differential expression and genome-wide DNA methylation in the same cardiac biopsies, we also determined whether differential promoter methylation of CGIs inversely correlated with corresponding gene expression, DMCs were identified by association with differentially expressed transcripts (*P* < 0.05, Table S4). A scatterplot was then generated using DEGs and their associated DMCs, revealing a clear and significant inverse correlation between the identified 211 DMCs and related genes’ expression in ICM (Pearson *P* = 1.6 × 10^−7^) but not NICM (Pearson *P* = 0.45) subjects (Fig. 3f, g).

### Genome-wide gene expression and DNA methylation reflects metabolic gene reprogramming

**Fig. 4:**
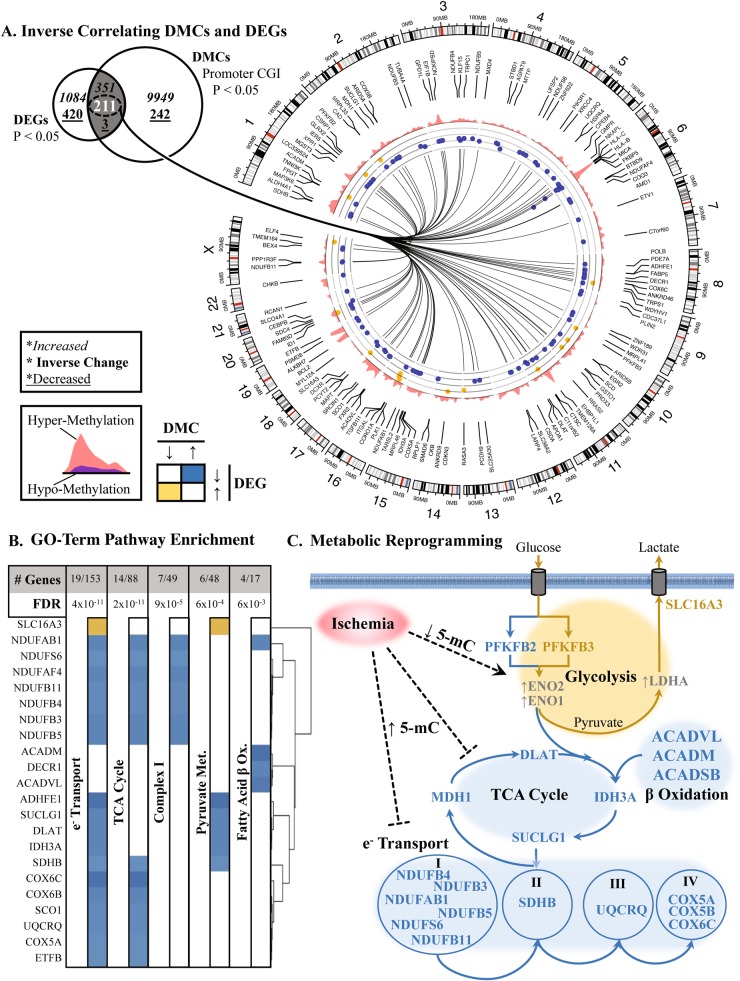
Candidate DNA methylation response genes. **a** Promoter-associated DMCs (*P* < 0.05) found within CGI associated with inversely DEGs (*P* < 0.05). **b** Top 5 gene ontology (GO)-term enrichment analysis of 211 functionally-overlapping DMCs. **c** Schematic illustrating the proposed metabolic reprogramming consistent with differential methylation of key intermediate genes. Key: yellow is hypomethylation with increased RNA levels; blue is hypermethylation with reduced RNA levels. ChrY was omitted because no DMCs or DEGs were found in this chromosome

The 211 DMCs corresponding with 124 DEGs were then viewed on a circular genome plot in combination with a genome methylation density track, showing widespread DMC genomic distribution with predominant hypermethylation and gene suppression (Fig. 4a). Gene set enrichment analysis using WEB-based GEne SeT AnaLysis Toolkit (WebGestalt) [32] was used to populate molecular pathways within the Reactome pathway database [33]. Although 124 genes is typically too small to achieve statistical significance, the gene network “Citric Acid (TCA) Cycle and Respiratory Electron Transport” was robustly enriched (Bonferroni-adjusted *P* *<* 0.0001), which included 19 DEGs associated with Complex I (NDUFS6, NDUFAF4, NDUFAB1, NDUFB4, NDUFB5, NDUFB11), Complex II (SDHB), Complex III (UQCRQ), Complex IV (COX5A, COX5B, COX6C), TCA Cycle (ADHFE1, DLAT, IDH3A, SUCLG1), and transporters (ETFB, SLC16A3) (Fig. 4b, Table S5). Furthermore, nearly all (18/19) significantly regulated genes associated with this pathway were transcriptionally suppressed. The only exception was SLC16A3, an induced (1.8-fold, FDR < 0.05) and hypomethylated (11%, *P* = 0.04), a lactate and ketoacid transporter. This metabolic pattern was further expanded by pulling all metabolic intermediates from the list of 211 DMCs, which defined a clear profile consistent with relative methylation-associated suppression of oxidative metabolism and possible induction of anaerobic glycolysis (Fig. 4c). Together these findings are consistent with both the EZH2-associated suppressive gene signature and the previously known metabolic substrate switching in heart failure [7].

### Differential methylation of Krüppel-like factor response element occurs in ICM

**Fig. 5:**
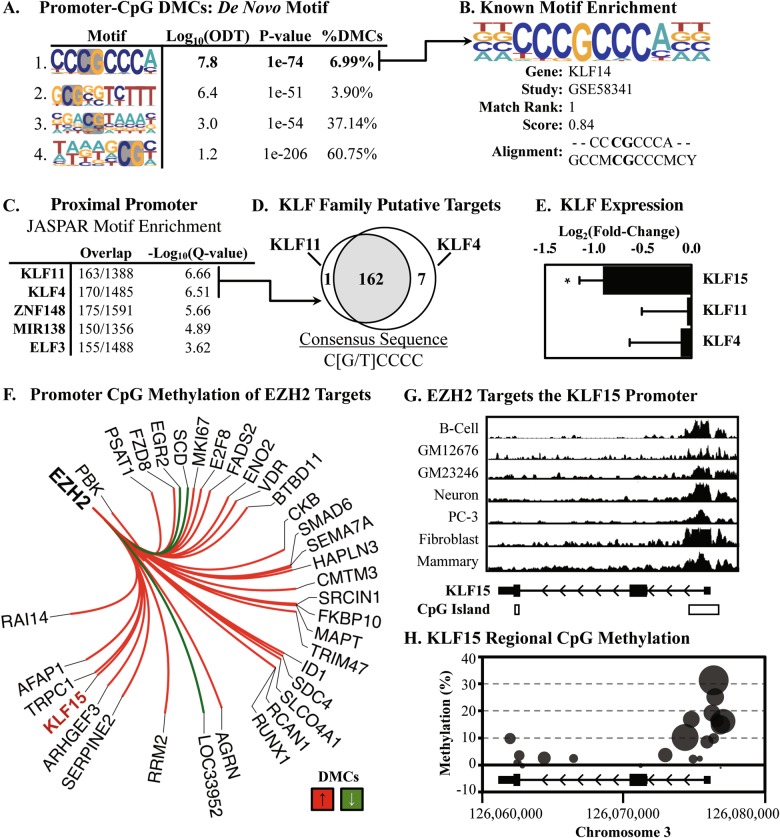
KLF15 and its downstream targets are differentially hypermethylated in ICM and targeted by EZH2. **a** Neighboring genomic regions +/− 10BP from promoter CGI DMCs (*P* < 0.05) used for de novo motif discovery via HOMER, with odds detection threshold (ODT), hypergeometric enrichment *P*-value, %DMCs aligning to the sequence, and relative location of consensus to the CpG for the top 5 motifs. **b** The motif associated with the highest ODT contains CpG site, enriched by known human motifs from HOCOMOCO v11 database. **c** Consensus sequence enrichment analysis of DEGs based on JASPAR database. **d** Overlap of target genes responsible for KLF11 and KLF4 enrichment. **e** Expression of KLF family members in Pre-LVAD left ventricular myocardium exhibiting high degree of consensus sequence homology (CG/TCCCC). **f** ENCODE ChIP-sequencing dataset for EZH2 gene targets possessing differentially-methylated promoter CpG Islands were arranged by chromosome location. **g** KLF15 promoter region viewed in accordance with multiple EZH2 ChIP-Sequencing datasets acquired from the UCSC Genome Browser, illustrating a conserved peak with the promoter CpG Island of KLF15. **h** Bubble plot illustrating differential methylation of all KLF15-associated CpG sites, with bubble size as -Log_10_(*P*-value)

Because both promoter methylation and EZH2-mediated histone methylation are known to mask the binding of transcriptional activators, we sought to identify response elements disproportionately present within the differentially-methylated regions. To identify these consensus sequences, the neighboring region (+/−10 bases) surrounding DMCs was used to generate and rank position-weighted matrices via Hypergeometric Optimization for Motif EnRichment (HOMER), an algorithm that compares the presence of DNA patterns in the “target list” (i.e. DMCs, *P* < 0.05) to that of background DNA sequences while correcting for CpG content [34]. The resulting motifs were ranked by Log_10_(Odds Detection Ratio) and *P*-value (Fig. 5a). The most enriched de novo motif was both highly specific (*P* = 1.0 × 10^−74^, Log_10_(ODT) = 7.8) and centered at the CpG locus. Alignment of this motif via Tomtom [35] algorithm revealed consensus of the CCCGCCC motif region with response elements of numerous human Krüppel-like factors (KLFs), with KLF14 as the most significantly enriched (Fig. 5b).

To determine which, if any, of the identified transcription factors including KLFs might be epigenetically regulated via response element interference, we performed a motif enrichment using the same JASPAR database, this time within the proximal promoter (−1500 to +500) of DEGs in ICM relative to NICM (*P* < 0.05, Fig. 5c, Table S6). We again found KLFs as most statistically enriched, albeit different members (KLF11 and KLF14). Because KLFs are known to possess highly-overlapping CpG-rich response element sequences (Fig. 5d), we examined all KLFs for differential expression in ICM relative to NICM (*P* < 0.05), revealing only KLF15 as differentially expressed in ICM relative to NICM (2-fold decrease, *P* < 0.05, Fig. 5e). Altogether, these observations support that KLF15 target genes are likely suppressed by disruption of the KLF-binding motif via CpG methylation.

### Targets of upstream epigenetic repressor EZH2 are hypermethylated

While the downstream effects of differential methylation depict a process of metabolic reprogramming, it remained unknown whether EZH2 might coordinate with DNA methylation to regulate metabolic gene expression. Binding targets of EZH2 via an ENCODE ChIP-sequencing dataset (ENCSR000AUZ) was inspected for genes possessing DMC’s, revealing 31/34 (94%) EZH2 target genes with promoter-associated CpG Island hypermethylation (Fig. 5f). Among these, the KLF15 promoter was found as a target of both EZH2 binding and CpG methylation. To validate the KLF15 promoter-binding capacity of EZH2, we cross-referenced multiple tissues using available ChIP-sequencing data which all showed EZH2 binding peaks at the CpG island within the KLF15 promoter (Fig. 5g). We also inspected all CpG sites associated with KLF15, which revealed a robust and widespread hypermethylation of the CpG island within the KLF15 promoter (Fig. 5h), which overlapped significantly with the EZH2 binding peak. Altogether, these observations support that CpG methylation and EZH2 may together co-regulate KLF15.

### EZH2 suppresses KLF15 in a SET domain-dependent manner

**Fig. 6:**
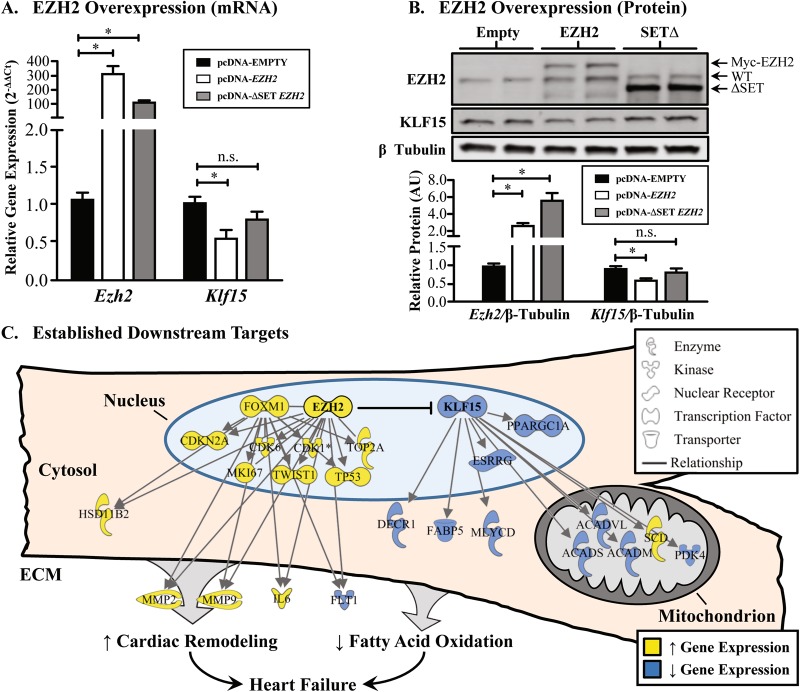
Novel EZH2-mediated suppression of KLF15 describes convergence of both metabolic and structural remodeling in heart failure. **a** Quantification of mRNA via RT-PCR, and **b** protein via Western blotting of *Ezh2* and *Klf15* following 48 h transfection of empty pcDNA (pcDNA-EMPTY), myc-tagged human EZH2 (pcDNA-EZH2), or SET-domain deleted EZH2 (pcDNA-ΔSET-EZH2) into H9c2. **c** Integrative visualization of known downstream co-regulated targets of EZH2-FOXM1 and KLF15, including the novel gene suppression of KLF15 by EZH2. *P < 0.05 by 1-Way ANOVA with Tukey’s multiple comparisons test

Because EZH2 has been shown to facilitate CpG methylation by recruiting DNA methyltransferases to target genes for EZH2-mediated gene repression in other tissues [28], we sought to determine whether EZH2 overexpression, as seen in ICM, is sufficient to suppress KLF15 expression. Lipofectamine-based overexpression of plasmid-derived human EZH2 (pcDNA-EZH2) in cultured H9c2 cardiac myoblasts was performed, resulting in a significant (~2-fold, *P* < 0.05) suppression of KLF15 gene expression and protein levels (Fig. 6a, b). To determine whether the suppression of KLF15 via EZH2 requires its catalytically-active SET domain, overexpression of EZH2 lacking its SET-domain (pcDNA-ΔSET-EZH2) was performed. In contrast to the full-length EZH2 overexpression, ΔSET-EZH2 failed to suppress KLF15 to a significant degree, suggesting that EZH2-mediated suppression of KLF15 occurs in a SET-dependent manner.

Combining the known downstream targets of EZH2 and KLF15 via IPA® revealed a marked pattern consistent with induction of co-regulated genes of EZH2-FOXM1 heterodimer, along with a contrasting signature of KLF15-mediated suppression of key oxidative metabolic genes via inhibition by EZH2 (Fig. 6c). Altogether, these observations implicate EZH2 as a possible regulator of both the structural remodeling and metabolic reprogramming that are known to occur in ICM.

## Discussion

Despite advances in both preventive measures and interventional approaches, ischemic heart disease remains the most common cause of heart failure, a terminal disease that affects nearly 6 million individuals in the United States. In general, heart failure is the clinical endpoint of many diseases including hyperlipidemia, hypertension, valvulopathy, and hereditary or environmental factors [36]. Because of such patient heterogeneity, current medical management remains limited to addressing the symptoms of reduced cardiac output and volume overload. Exposing the underlying etiologic mechanisms would therefore greatly accelerate the development of more effective and individualized therapies.

In the current study, we used human cardiac samples to identify an epigenetic signature capable of describing the known metabolic switch that occurs in the failing ischemic heart. We also identified EZH2 as a possible regulator of cardiac metabolic and structural reprogramming, owing to its dual role as both gene suppressor, via SET-dependent PRC2 subunit, and gene activator, as heterodimer with FOXM1. Altogether, we believe that epigenetics encodes a metabolic memory in failing hearts, the reversal of which may repair the ischemic and failing heart.

### Epigenetic reprogramming of cardiac metabolism

Although nutrient-dependent changes in cardiac metabolism have been known to occur for over 50 years via so-called Randle Cycle [37], the specific molecular cues that both maintain and switch its substrate preference remain unknown. Our observations support that epigenetic cues, specifically differential DNA methylation, governs this metabolic switch in the heart to a greater extent in response to cardiac ischemia. Our analysis reveals a complementary transcriptional program that mediates oxidative metabolic gene suppression via genome-wide promoter hypermethylation of genes associated with electron transport, TCA cycle, and fatty acid β oxidation. Conversely, we found hypomethylation of anaerobic glycolytic genes, altogether depicting a ‘regression to the fetal gene program’ [7]. Therefore, our observations support that DNA methylation is a contributing mechanism by which the heart is metabolically reprogrammed by ischemia.

### EZH2 as a candidate regulator of cardiac CpG methylation

EZH2 emerged early in our study as a likely transcriptional regulator of gene expression in ICM, both as transcriptional activator with FOXM1 and as epigenetic repressor as histone methyltransferase with the polycomb repressor complex 2 (PRC2). Its paradoxical function as activator and repressor has been shown in cancer, where it accelerates the malignant transformation of human cells by governing their switch from oxidative to anaerobic metabolic preference, as process long known as the Warburg effect [38]. Metabolomics profiling of melanoma has demonstrated the role of EZH2 in the suppression of oxidative pathways and increased glycolytic activity [39]. In addition, DNMT1 has been shown to physically interact with EZH2 to provide site-specific differential methylation in prostate cancer [40]. Although DNMT1 is classically associated with germline methylation, it prevents P53-mediated apoptosis in response to hypoxia [41].

**Fig. 7:**
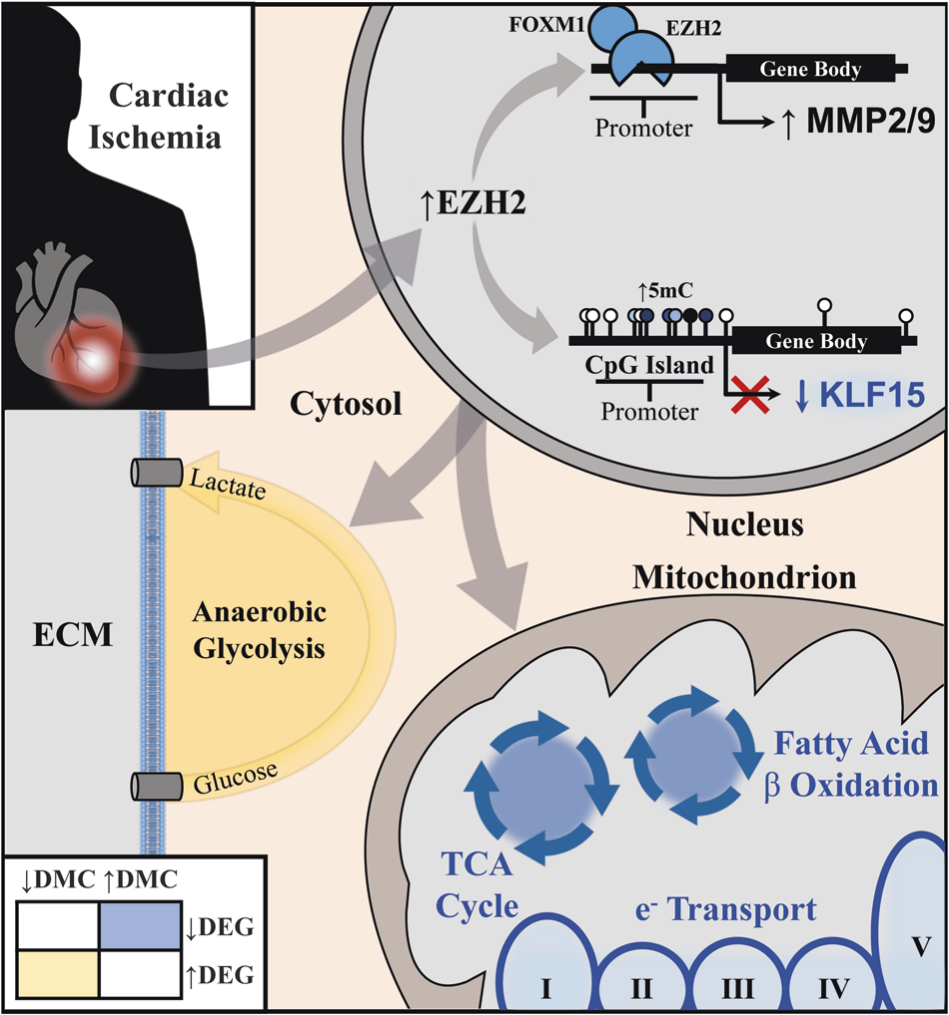
Scheme for the ischemic programming and downstream epigenetic regulation via EZH2 and KLF15. Human ischemic heart failure presents a distinct DNA methylation signature associated with known changes from oxidative metabolism to anaerobic glycolysis. Bioinformatic analysis strongly supports the involvement of differential methylation mediated by EZH2 to coordinately decrease the expression of the transcription factor KLF15 and its function via response element methylation

In the heart, it has been shown in a brief communication that suppressing EZH2 enhances the differentiation of cardiac fibroblasts into beating cardiomyocytes [42]. Our observations therefore provide rationale in human tissue that the induction of EZH2 likely produces a phenotypic switch of ischemic cardiomyocytes, thereby resulting in colliquative myocytolysis. Altogether, these observations lead us to hypothesize that an EZH2-DNMT complex, a mechanism that requires additional exploration, may coordinate the ICM-associated differential methylation and downstream suppression of key metabolic enzymes and transcription factors such as KLF15 (Fig. 7).

### KLF15 as an epigenetically-tuned regulator of cardiac metabolism

In addition to the direct impact of differential CpG methylation on metabolic intermediate enzymes, our analysis also supports that gene suppression of transcription factor KLF15 occurs in ICM. As an established regulator of myocardial fatty acid utilization, KLF15 is suppressed during fetal cardiac development and rapidly induced in the days following birth, during which time the fetal heart becomes reliant on oxidative metabolism as it is starved of a rich supply of placental nutrients [43]. Conversely, suppression of KLF15 has been shown to occur in heart failure, an expression pattern that closely reflects lipid utilization. Furthermore, KLF15 has been shown to negatively regulate adverse cardiac remodeling and hypertrophy in animal models [44–46]. In the current study we provide novel evidence suggesting that cardiac ischemia triggers its epigenetic regulation via promoter hypermethylation and EZH2-mediated suppression. Our in silico analyses suggest that differential promoter methylation of both KLF15 and its targets may be coordinated to a greater degree in ICM relative to NICM.

Examining the Cancer Genome Atlas (TCGA) via UALCAN [47] suggests that this inverse relationship between EZH2 and KLF15 exists in many tissues and appears similarly disrupted in malignancy (Fig. S4). Furthermore, we provide preliminary evidence that in vitro EZH2 negatively regulates KLF15 in cardiomyocytes, doing so in a SET-dependent manner. Although further mechanistic experiments are required, we believe this observation highlights a convergence of multiple epigenetic mechanisms to control the transcriptional activity of KLF15 in response to cardiac ischemia.

### Cross-site validation of ICM vs. NICM differential gene expression

The gene expression changes in ICM relative to NICM have been previously studied using sizeable cohorts and microarray-based platforms. Our analysis yields a consistent pattern of gene expression with that published by Kittleson et al. [48], which similarly shows “fetal gene program induction.” However, their statement pertained primarily to biomarkers that have been previously associated with heart failure, including MYH6, BNP, and ANP. Our analysis yields a relative suppression of B-natriuretic peptide (BNP) in ICM (2.5-fold, *Q* = 0.02), contrary to its induction in ICM relative to NICM from the Kittleson et al. study. Another published microarray-based analysis of 313 human ICM, NICM, and non-failing hearts (GSE57345) shows more robust co-directional gene expression changes in ICM vs. NICM. Of the 171 overlapping DEGs, 143 (84%) are co-expressed in the same direction (*P* < 0.05, FPKM > 2) [49]. Among these, rate-limiting glycolytic gene PFKFB3 is induced, with APOA1 suppressed, which we have expanded upon to correlate with differential methylation of CGI located within the proximal promoter (Table S6).

### Limitations of the current study

Although the current study demonstrates the importance of DNA methylation as a likely contributor to the gene expression changes that distinguish ischemic heart failure, key limitations must be considered when interpreting the results. Although we provide both histologic and in silico evidence that the effects of cellular heterogeneity are unlikely to confound our analysis, our whole-tissue analysis precludes us from defining cell-type specificity of the observed epigenetic changes. Although the cross-validated microarray datasets included female subjects, we were unable to include females in the current study to test for sexually dimorphic gene expression and/or epigenetic patterns. Therefore, we cannot yet infer the differential effects of ischemia on DNA methylation in female heart failure patients. Future studies should consider the impact of ischemic heart failure on DNA methylation by sex, race, and other heart failure etiologies. Lastly, the current study provides correlative evidence supporting the regulatory influence of promoter-associated DNA methylation on gene expression in human heart failure. Therefore, future mechanistic studies will explore the causative role of differential DNA methylation and EZH2 in heart failure pathogenesis and therapy.

Our observations reveal a strong correlation between DNA methylation and the gene expression associated with the known metabolic shift that occurs in ischemic heart failure. Cardiac ischemia also correlates with induction of EZH2, an epigenetic regulator that may coordinate with differential DNA methylation to suppress KLF15 and other downstream metabolic gene targets (Fig. 7). While these metabolic gene changes may initially protect the myocardium from energy collapse under ischemic conditions, it is possible that chronic exposure to ischemic stress potentiates the adverse cardiac structural and metabolic remodeling that defines heart failure. Although mechanistic studies are required to determine the direct role of both DNA methylation and EZH2 in heart failure, defining the epigenetic and/or metabolic machinery contribute to precision-based interventions for ischemic cardiomyopathy.

## Electronic supplementary material

Supplemental 1
