# Supplementary material for: Genome-wide DNA methylation encodes cardiac transcriptional reprogramming in human ischemic heart failure
Source: Lab Invest. 2018 Aug 8;99(3):371–86. doi: 10.1038/s41374-018-0104-x (PMC6515060; doi:10.1038/s41374-018-0104-x)
Supplement: Supplementary file 1 — Supplemental 1 [file 41374_2018_104_MOESM1_ESM.docx]

SUPPLEMENTAL METHODS, FIGURES, AND TABLES

**Genome-Wide DNA Methylation Encodes Cardiac Transcriptional Reprogramming in Human Ischemic Heart Failure**

Mark E. Pepin, Chae-Myeong Ha, David K. Crossman, Silvio H. Litovsky,
Sooryanarayana Varambally, Joseph P. Barchue, Salpy V. Pamboukian, Nikolaos A. Diakos, Stavros G. Drakos, Steven M. Pogwizd, Adam R. Wende

**EXPANDED METHODS**

[Fig. S1: Outlier Analysis 3](#_Toc514857936)

[Fig. S2: Histological Analysis 4](#_Toc514857937)

[Fig. S3: Global Patterns in Gene Expression 5](#_Toc514857937)

[Fig. S4: Relationship between EZH2 and KLF15 in human Cancer 5](#_Toc514857937)

[Fig. 1: Patient Health Metrics Correlation. 6](#_Toc514857938)

[Fig. 2: RNA Sequencing Analysis. 7](#_Toc514857939)

[Fig. 3A: Unsupervised PCA of CpG Methylation. 8](#_Toc514857940)

[Fig. 3B: Distribution of Methylation by Genomic and CpG Annotation. 11](#_Toc514857941)

[Fig. 3D: Heatmap and Hierarchical Clustering of Differential CpG Methylation (P < 0.05) 12](#_Toc514857942)

[Fig. 4A: CpG Site Homology, De Novo Motif Discovery. 13](#_Toc514857943)

[Fig. 4B: Known Motif Enrichment. 16](#_Toc514857944)

[Fig. 5A: Correlation between DEGs and DMCs – DMC vs. DEG for NICM Patients (n = 6). 18](#_Toc514857945)

[Fig. 5B: Correlation between DEGs and DMCs – DMC vs. DEG for ICM Patients (n = 5). 20](#_Toc514857946)

[Fig. 6: Combined DMC vs. DEG for both NICM and ICM Subjects. 22](#_Toc514857947)

[Fig. 5C: Scatter Plot of KLF15 Promoter Methylation with Inverse Gene Expression 24](#_Toc514857948)

[Fig. 5D Bubble Plot and Gene Density of KLF15 Differential Methylation 27](#_Toc514857949)

[Fig. 6A: Merging RNA Sequencing with Inverse Differential DNA Methylation 27](#_Toc514857950)

[Fig. 6B: Hierarchical Pathway Analysis of DEGs with Inverse DMCs. 32](#_Toc514857951)

[Fig. 7B: EZH2 as a Putative Nodal Regulator with DNA Methylation. 34](#_Toc514857952)

[Supplemental Information: R Session Information. 37](#_Toc514857953)

**LIST OF FIGURES**

[Figure S1. Heatmap and Hierarchical Clustering of DNA Methylation with outlier LVAD_L (ICM subject). 3](file:////Users/pepinme/Box/Work/4_PhD/_Papers/Do/huHrt%20Study/v03%20NPG%20Lab%20Investig,%20Feb%2026/Resubmit/Pepin_NPG-LI_ISCH_Sup_v4mep.docx#_Toc514857960)

[Figure S2. Histological Analysis. 4](file:////Users/pepinme/Box/Work/4_PhD/_Papers/Do/huHrt%20Study/v03%20NPG%20Lab%20Investig,%20Feb%2026/Resubmit/Pepin_NPG-LI_ISCH_Sup_v4mep.docx#_Toc514857960)

[Figure S3: Gene Expression Analysis of ICM relative to NICM. A. Heatmap and hierarchical clustering of DEGs (*P* < 0.05). B. Unsupervised Principal Components Analysis (PCA). C. Transcriptomic fingerprinting using *Xcell*. 5](#_Toc514857961)

[Figure S4: Relationship between EZH2 and KLF15 in human Cancer. The Cancer Genome Atlas (TCGA) was used to define a correlation between EZH2 and KLF15 expression in both normal (black bars) and several cancer types. 6](#_Toc514857962)

[Figure S5. Unsupervised Principal Components Analysis of DNA Methylation. 10](file:////Users/pepinme/Box/Work/4_PhD/_Papers/Do/huHrt%20Study/v03%20NPG%20Lab%20Investig,%20Feb%2026/Resubmit/Pepin_NPG-LI_ISCH_Sup_v4mep.docx#_Toc514857963)

[Figure S6: Distribution of CpG Sites. 3-dimensional contour plot depicting the distribution of DMCs about CpG and genomic regions for (A) All CpG sites interrogated by the HumanMethylation450k Array and (B) Differentially-methylated Cytosines (P<0.05 and |% Change|>5). 12](#_Toc514857964)

[Figure S7. DEGs (P<0.05) containing promoter-associated inversely changing DMRs (P<0.05) were plotted by left ventricle samples obtained from non-ischemic cardiomyopathy(NICM) subjects. 20](file:////Users/pepinme/Box/Work/4_PhD/_Papers/Do/huHrt%20Study/v03%20NPG%20Lab%20Investig,%20Feb%2026/Resubmit/Pepin_NPG-LI_ISCH_Sup_v4mep.docx#_Toc514857965)

[Figure S8. DEGs (P<0.05) containing promoter-associated inversely changing DMRs (P<0.05) were plotted by left ventricle samples obtained from ischemic cardiomyopathy(NICM) subjects. 22](file:////Users/pepinme/Box/Work/4_PhD/_Papers/Do/huHrt%20Study/v03%20NPG%20Lab%20Investig,%20Feb%2026/Resubmit/Pepin_NPG-LI_ISCH_Sup_v4mep.docx#_Toc514857966)

[Figure S9. DEGs (P<0.05) containing promoter-associated inversely changing DMRs (P<0.05) were plotted by left ventricle samples obtained from all cardiomyopathy(NICM) subjects. 23](file:////Users/pepinme/Box/Work/4_PhD/_Papers/Do/huHrt%20Study/v03%20NPG%20Lab%20Investig,%20Feb%2026/Resubmit/Pepin_NPG-LI_ISCH_Sup_v4mep.docx#_Toc514857967)

**LIST OF TABLES**

[Table S1. Empirical DNA-Binding Protein Enrichment of Differentially-Expressed Gene Promoters (-1kB → +500B) with ENCODE ChIP-Sequencing Database. 38](#_Toc514858630)

[Table S2. EZH2 Expression Post-LAD Ligation (Tarnaski *et al.* PMID: 14679301;GDS488) 39](#_Toc514858631)

[Table S3. Methylation Distribution (P < 0.05) 39](#_Toc514858632)

[Table S4. Differentially Methylated Promoter CpG Islands with Inversely Expressed Genes (P<0.05). 39](#_Toc514858633)

[Table S5. Gene Set Enrichment of Transcriptionally-Active Promoter DMC's via WebGestalt using the Reactome Pathway Database. 46](#_Toc514858634)

[Table S6. Consensus Sequence Enrichment of Differential Expressed Gene (P < 0.05) Proximal Promoters (-1kB  +500B), using JASPAR database and Enrichr algorithm. 46](#_Toc514858635)

[Table S7: R Bioinformatics Packages and Required Dependencies 48](#_Toc514858636)

EXPANDED METHODS

This document summarizes the bioinformatics workflow that Pepin *et al.* used to identify a distinct epigenomic pattern consistent with metabolic reprogramming in human ischemic heart failure vs. non-ischemic heart failure. Most of the analyses were performed in the R statistical computing environment; however, multiple LINUX-based analytic tools were also used, and are referenced accordingly.

Fig. S1: Outlier Analysis

Figure S1. Heatmap and Hierarchical Clustering of DNA Methylation with outlier LVAD_L (ICM subject).


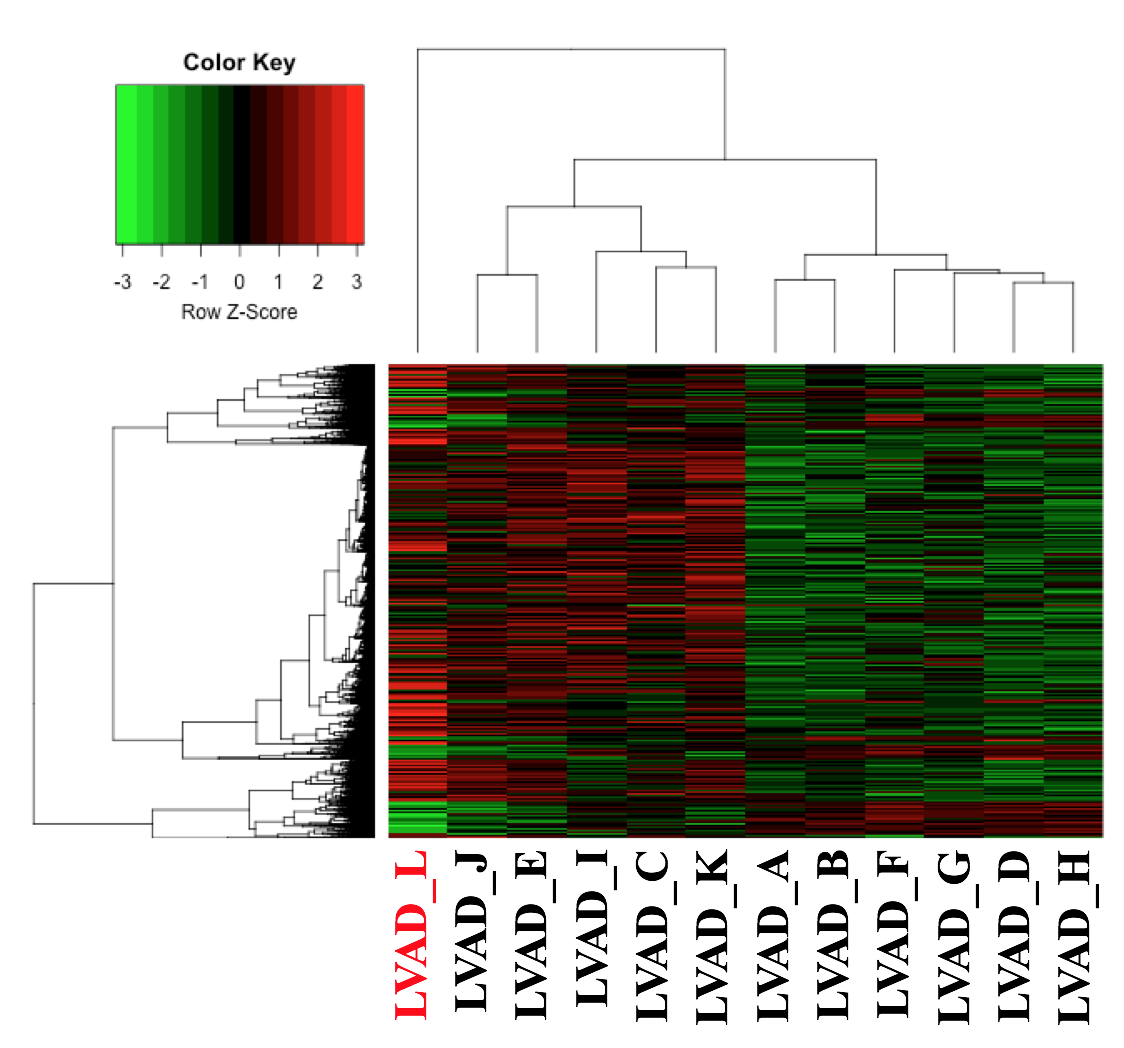


Before initiating the bioinformatics analysis of human left ventricular tissue, a visual outlier analysis was performed by hierarchical clustering and heatmap analysis of the genome-wide methylation. Shown in Figure A1, LVAD_L is an extreme outlier. Because we had noted a red color in the LVAD_L sample prior to submission for RNA sequencing and DNA Methylation, its removal was validated.

**Fig. S2: Histological Analysis**

**
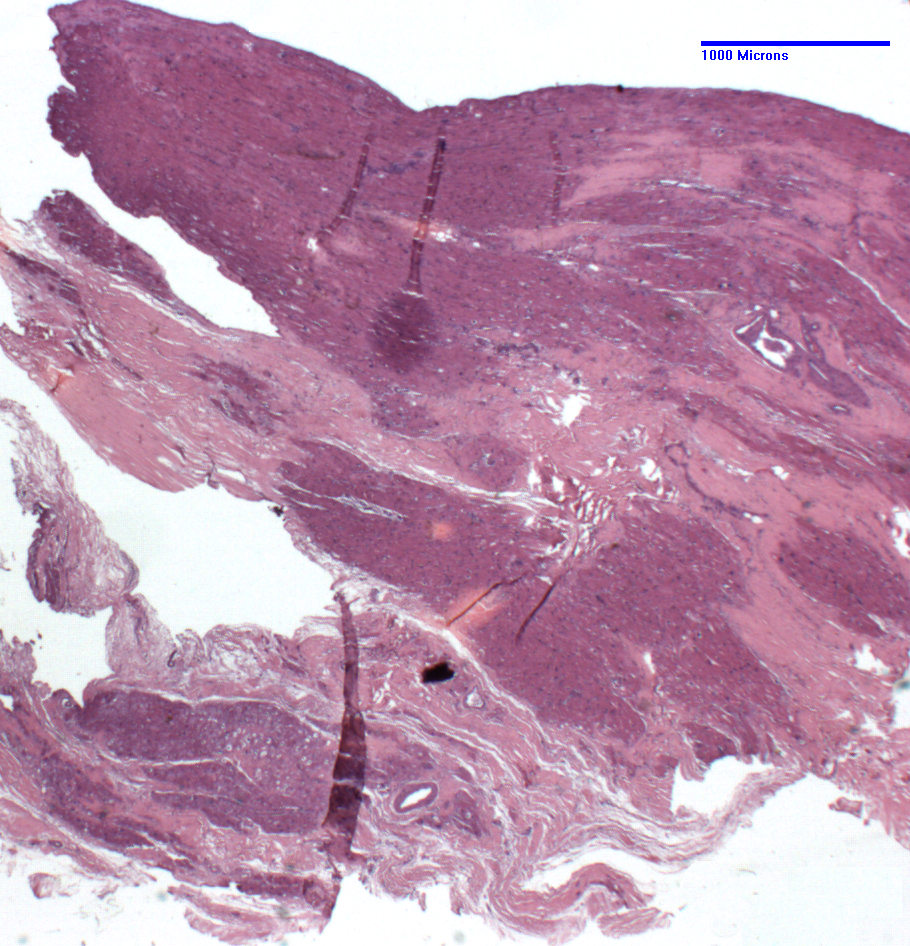
Non-Ischemic Ischemic**

C


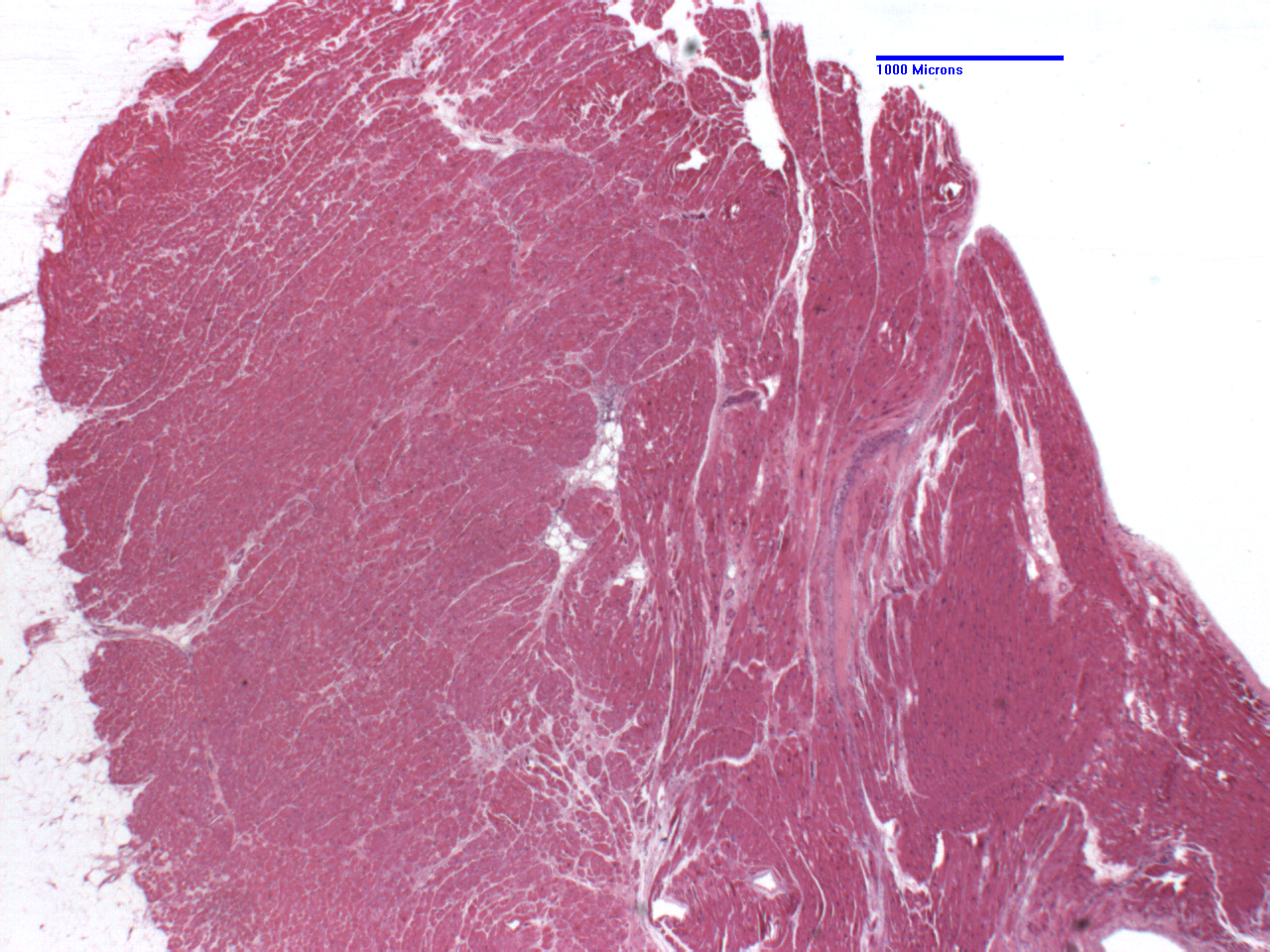


B


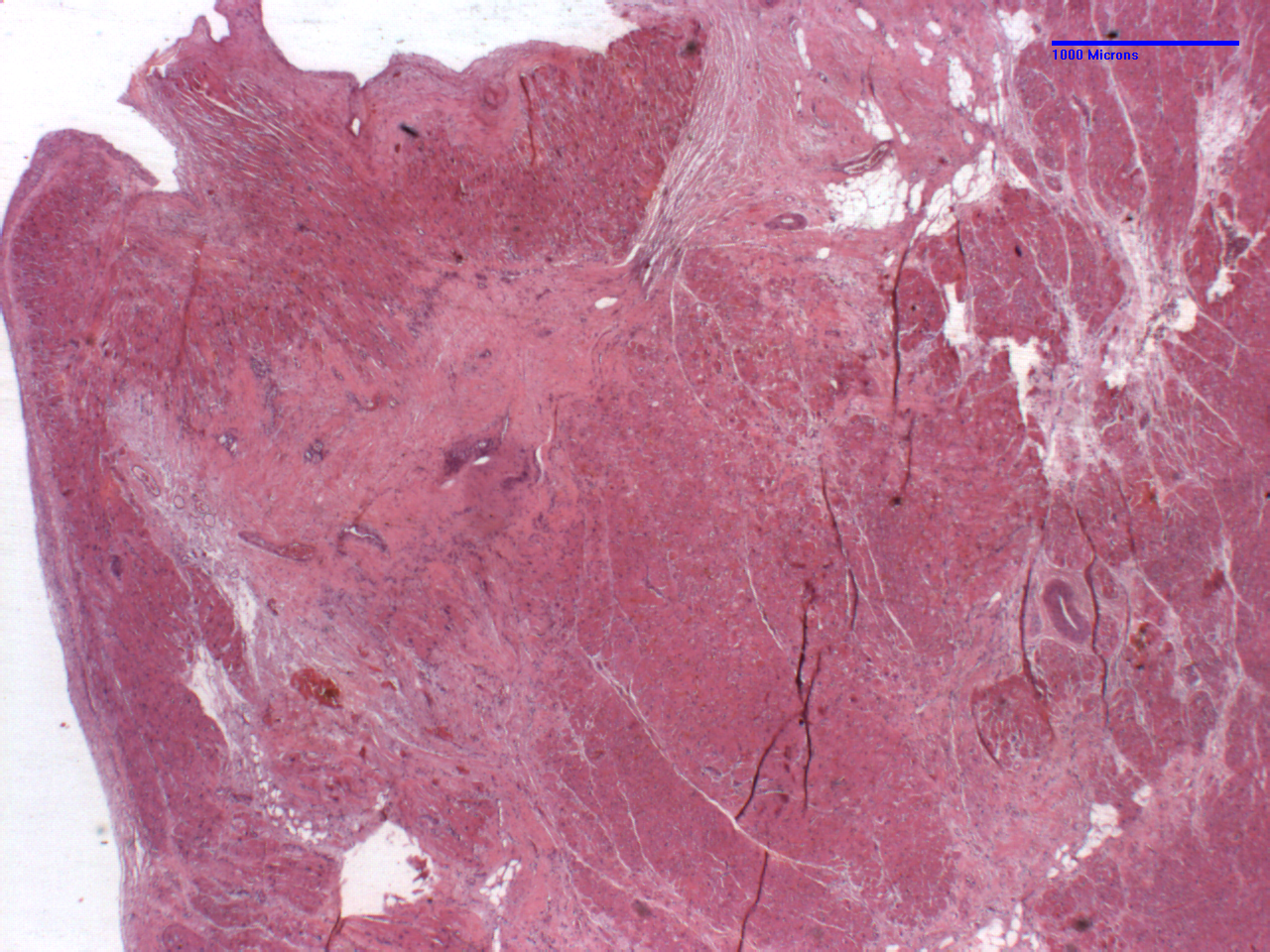


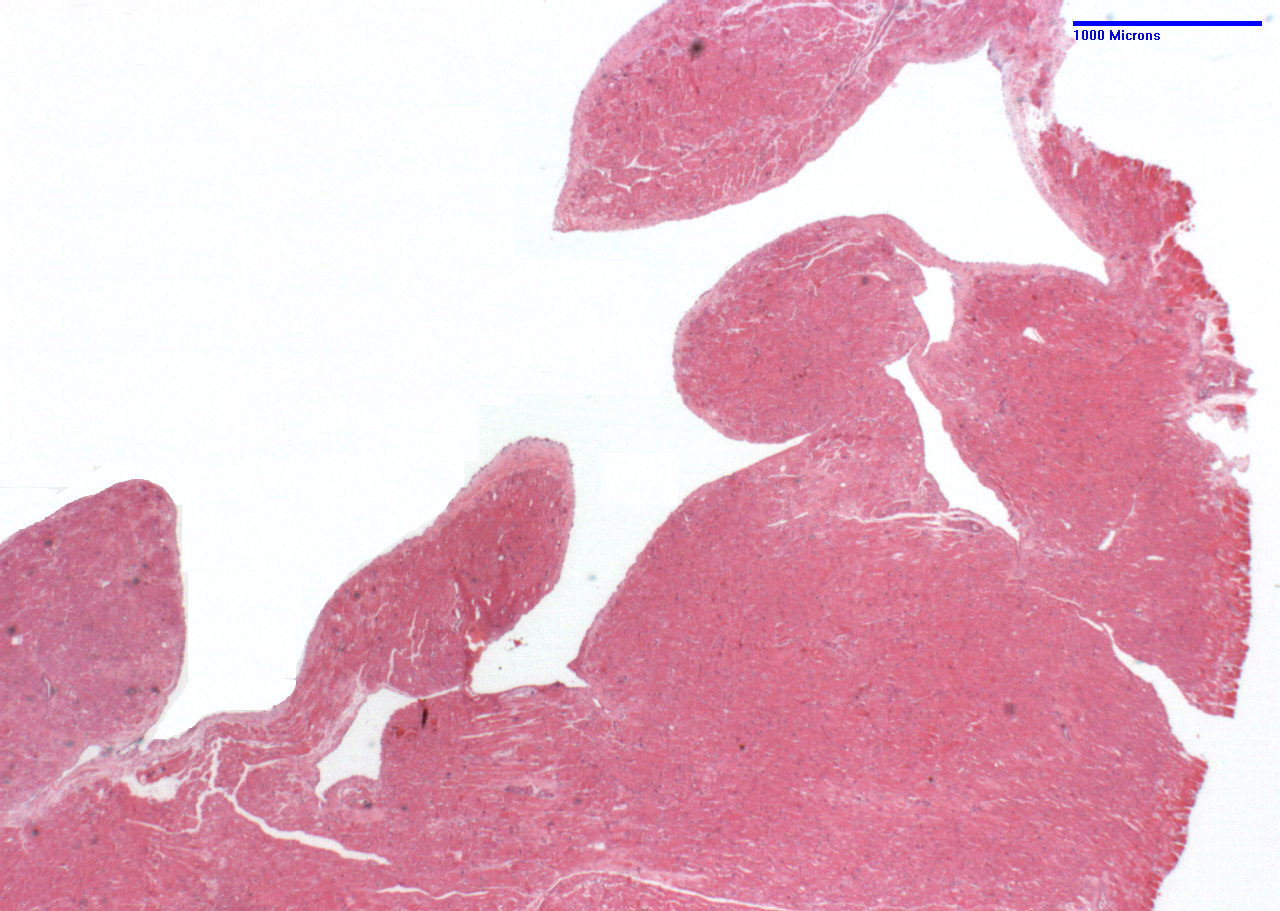


F


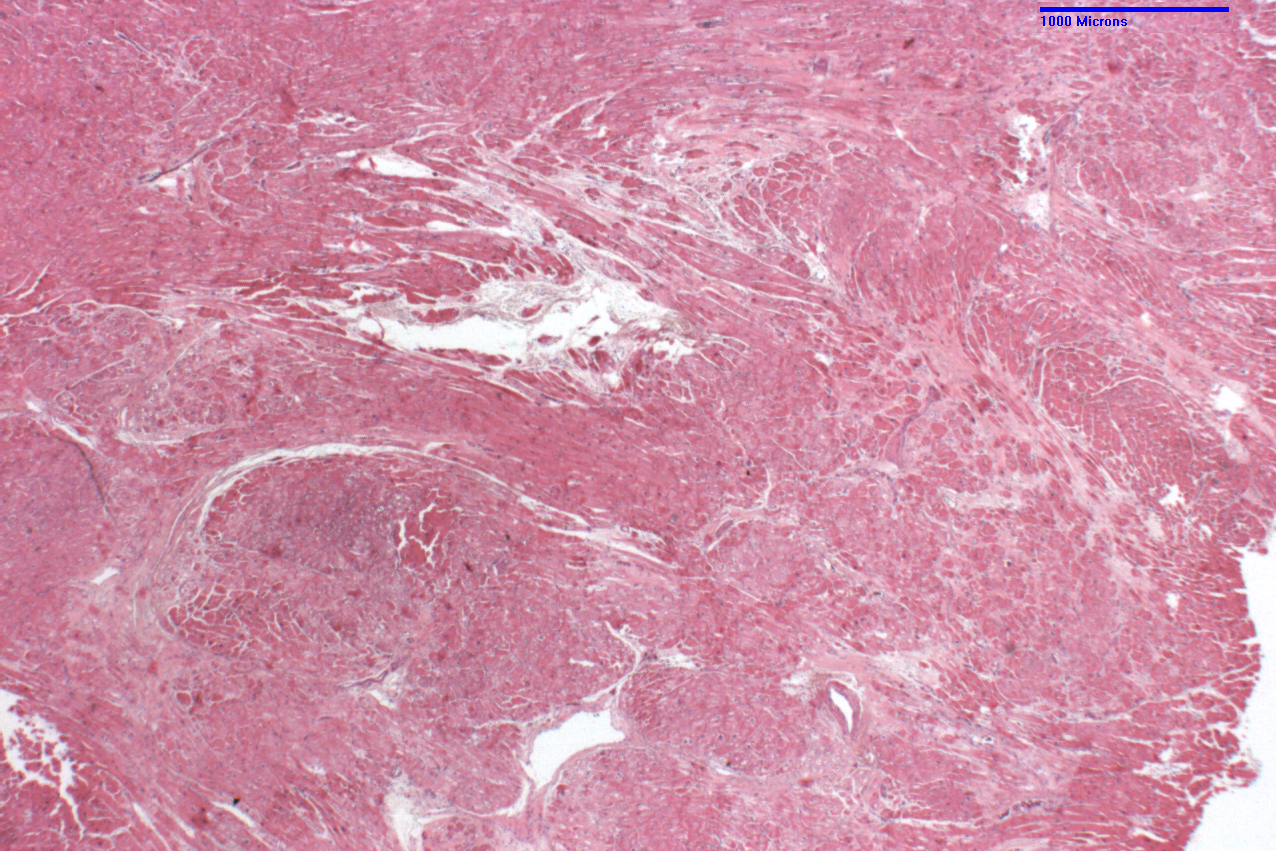


I


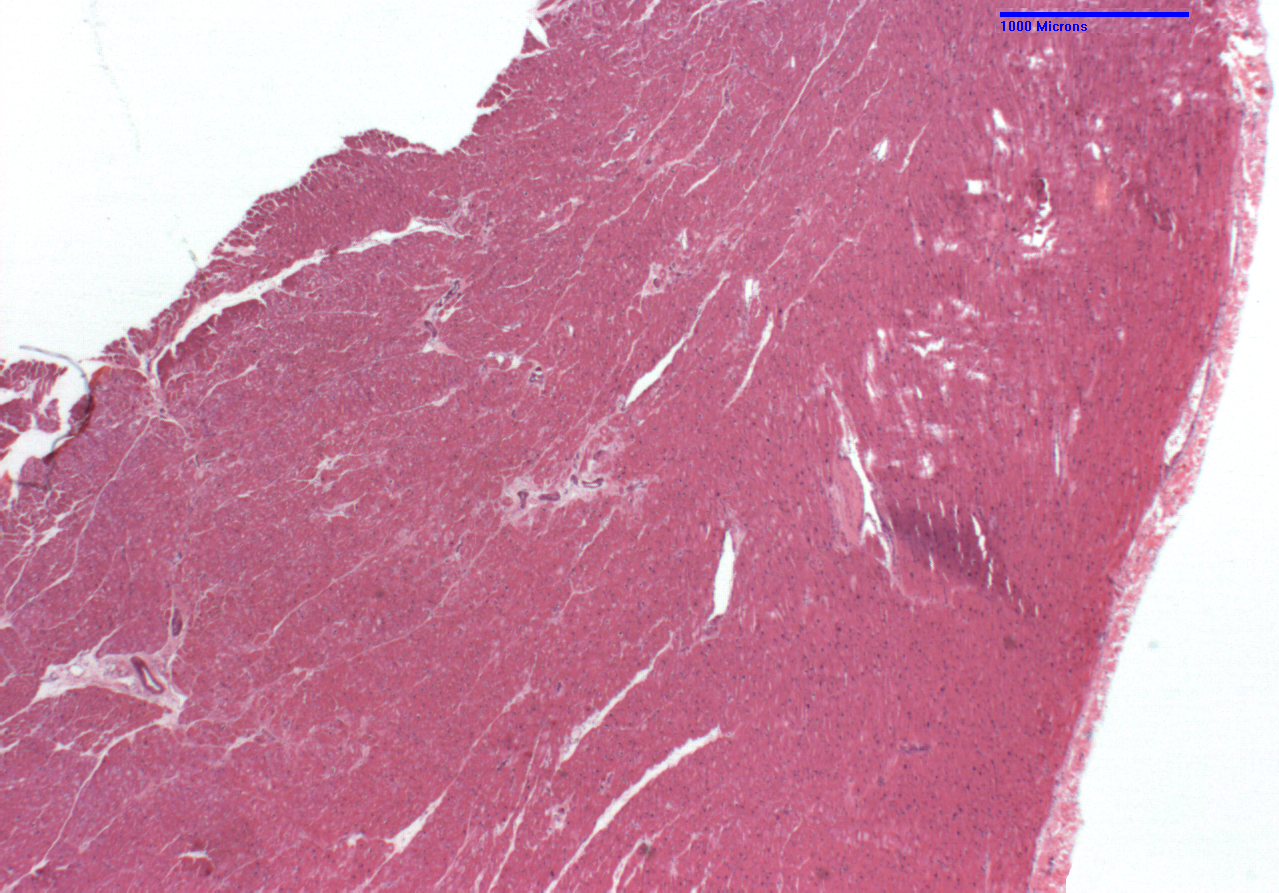


D


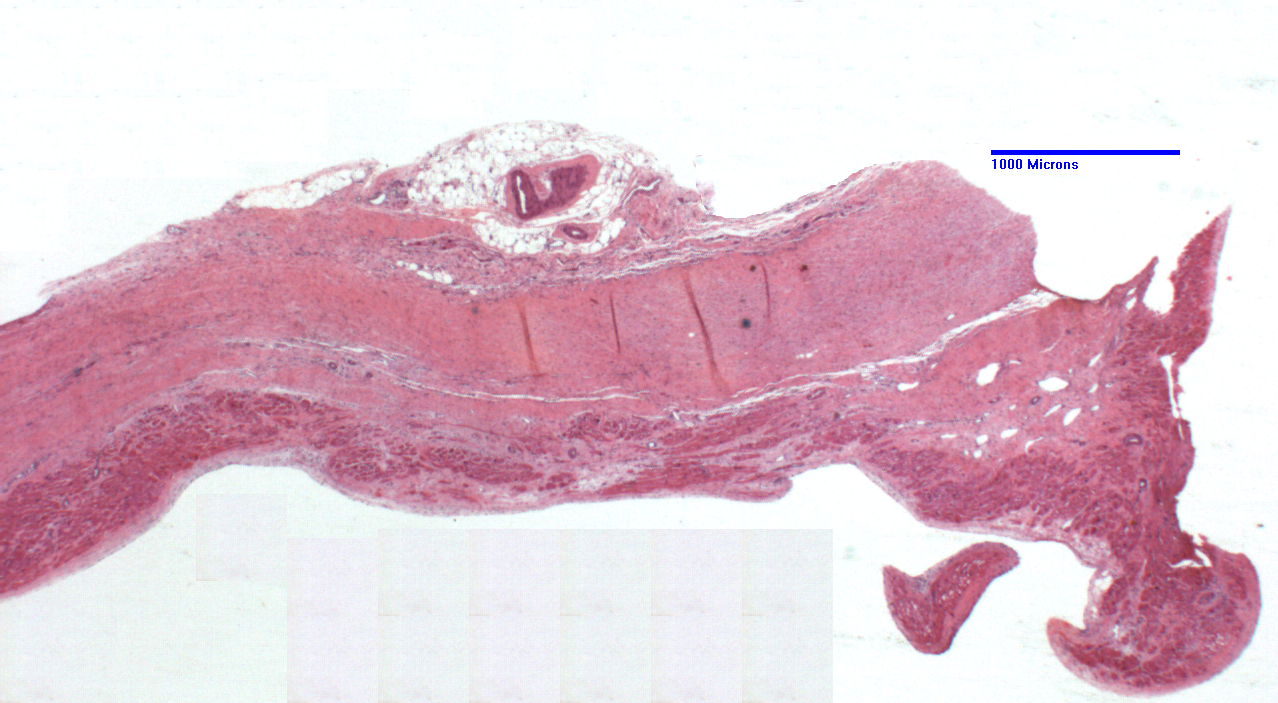


E

K

Fig. S3: Global Patterns in Gene Expression


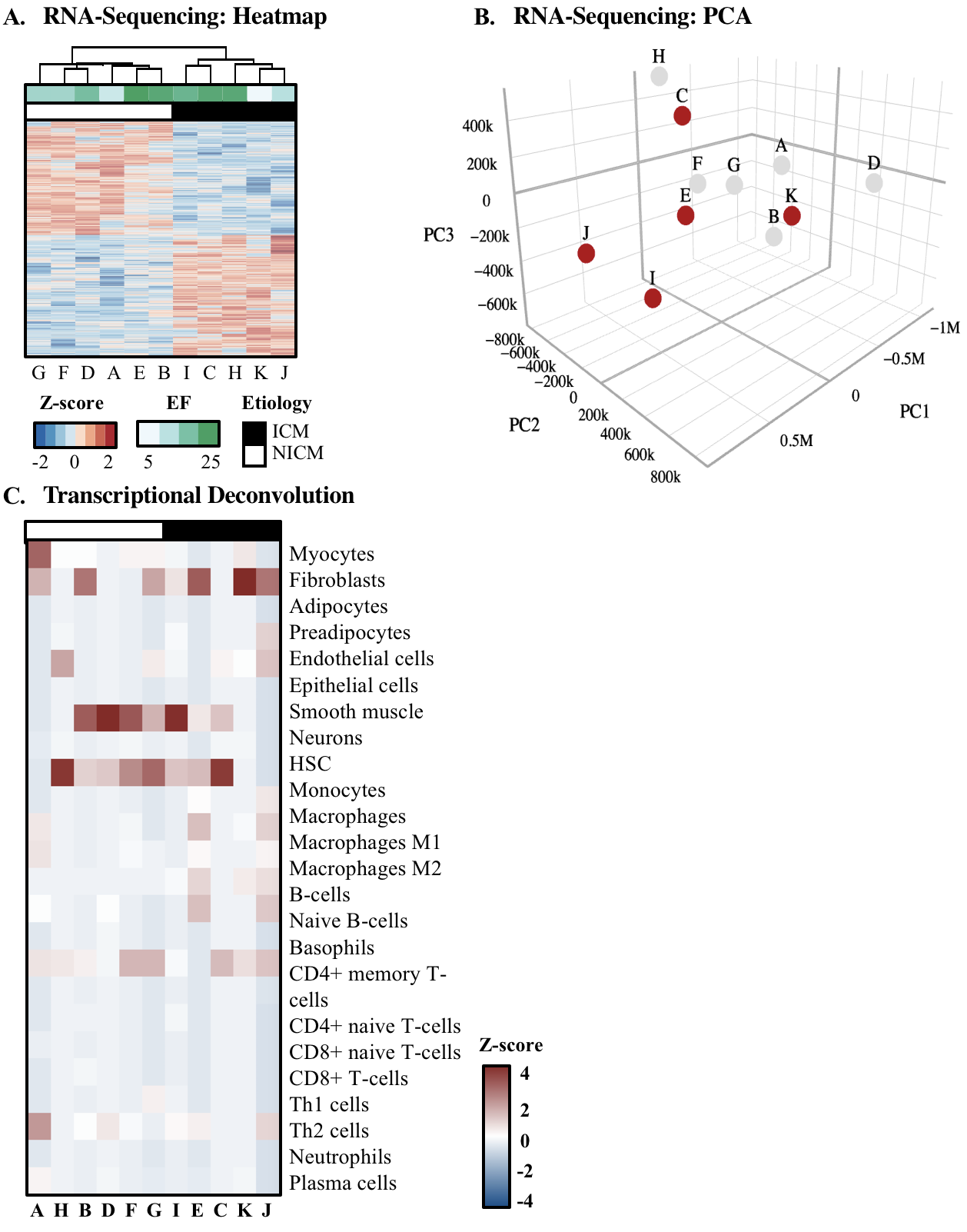


Figure S3: Gene Expression Analysis of ICM relative to NICM. A. Heatmap and hierarchical clustering of DEGs (*P* < 0.05). B. Unsupervised Principal Components Analysis (PCA). C. Transcriptomic fingerprinting using *Xcell*.


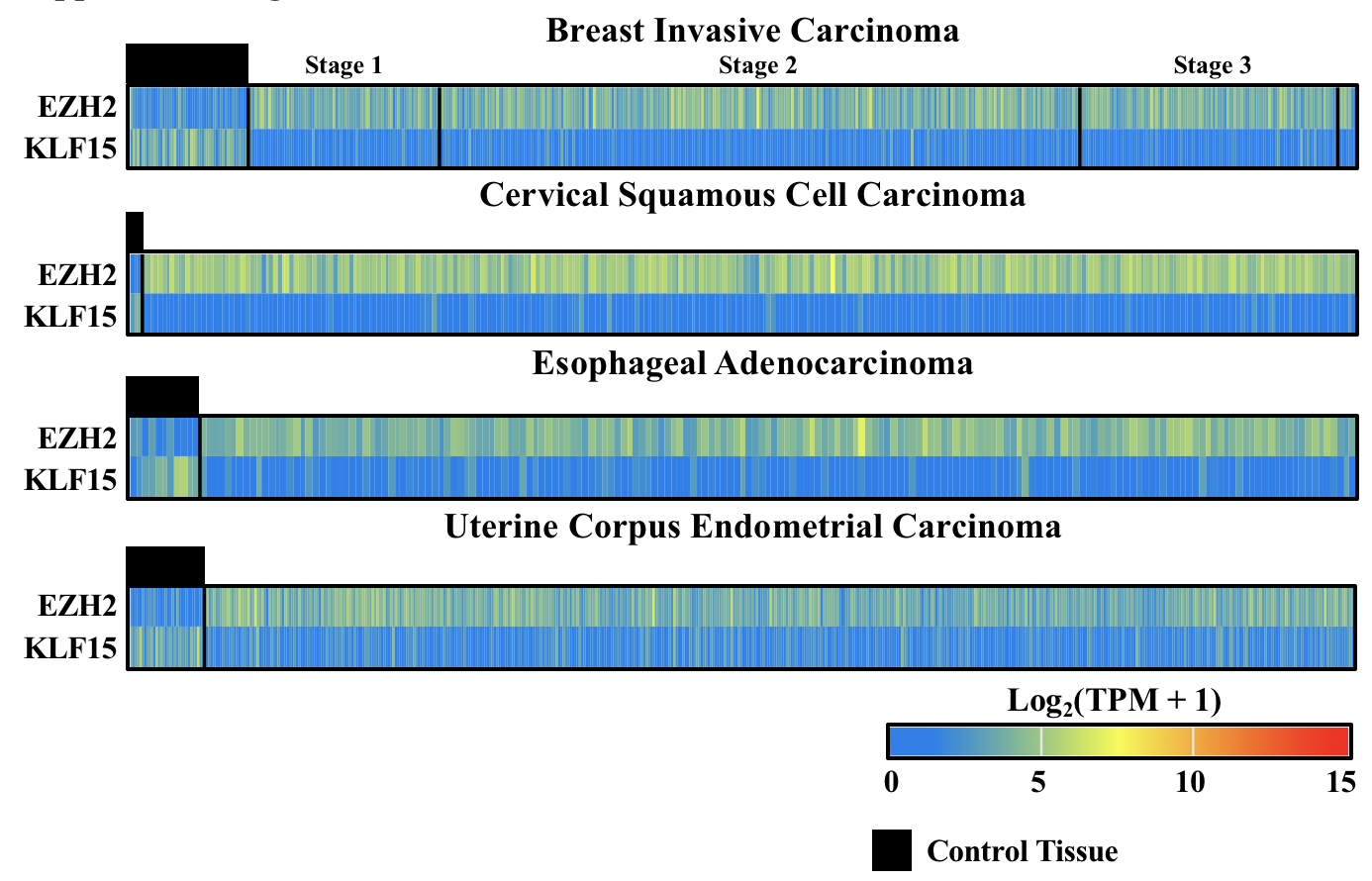


Figure S4: Relationship between EZH2 and KLF15 in human Cancer. The Cancer Genome Atlas (TCGA) was used to define a correlation between EZH2 and KLF15 expression in both normal (black bars) and several cancer types.

Fig. 1: Patient Health Metrics Correlation.

The first task in this project was to verify that the heart failure patients from which the cardiac tissues were obtained allowed for a simple comparison (i.e. not requiring multiple regression) between Ischemic and Non-Ischemic Cardiomyopathy, as a means of confirming that no confounding variables existed. We conclude that, although patient **Age is weakly correlated with** **Ischemic status (*P* < 0.07)**, our sample selection adequately controls for known risk covariates of heart failure and contributors to differential CpG Methylation. Furthermore, it is likely that the stringent age range selected yields a statistically trending albeit biologically irrelevant difference.

library(readxl)
library(dplyr)
library(Hmisc)
library(corrplot)
library(RColorBrewer)
library(kableExtra)
Patient_Data <- read_xlsx("../1_Input/1_Patient/Patient_Data.xlsx")
Patient_Data<-as.data.frame(Patient_Data)
# Print the table for use
Patient_Data %>% kable(format="latex",
 align="c",
 booktabs=T,
 caption="Patient Characteristics") %>%
 kable_styling(latex_options=c("striped",
 "condensed",
 "scale_down"))

# Format for Correlation
Patient_Data$Obese<-as.numeric(as.factor(Patient_Data$Obese))
Patient_Data$`Cardiac Index`<-as.numeric(as.factor(Patient_Data$`Cardiac Index`))
Patient_Data[is.na(Patient_Data)]<-""
Patient_Data$`Cardiac Index`<-as.numeric(as.factor(Patient_Data$`Cardiac Index`))
Patient_Data$Inotrope<-as.numeric(as.factor(Patient_Data$Inotrope))
rownames(Patient_Data)<-Patient_Data$Sample_ID
cor<-subset(Patient_Data, select=-c(Sample_ID, Sex))
cor<-data.matrix(cor)
cor.m<-rcorr(cor)
cor.r<-cor(cor)
paletteLength <- 100
myColor <- colorRampPalette(c("dodgerblue4", "white", "brown4"))(paletteLength)
p.mat<-cor.mtest(cor.r)$p
corrplot(cor.r,
 order="AOE",
 type="lower",
 method = "circle",
 hclust.method = "ward.D2",
 outline=TRUE,
 addrect = 3,
 col = myColor,
 tl.cex=0.7,
 tl.col="black",
 addgrid.col = NA)

Fig. 2: RNA Sequencing Analysis.

Fig. 3A: Unsupervised PCA of CpG Methylation.

Since EZH2 is known to interact with DNMT1, we sought to determine whether the EZH2 induction corresponded with increased promoter-associated CpG methylation, as determined by Illumina BeadChip HumanMethylation450k array. The first step was to determine whether the samples clustered on unsupervised Principal Components Analysis (PCA).

**An unbiased (unsupervised) Principal Components Analysis (PCA) was performed on all CpG Sites interrogated by the HumanMethylation450k array. From this figure, we observe a clear distinction between ICM and NICM, independent of statistical filtering.
library**(readxl) **library**(dplyr) **library**(plotly)
##Import Data Matrix
DMR.raw <- **read.csv**("../1_Input/3_Methylation/LVAD_ICM.v.NICM_Methyl450k.csv")
#Import the Index File
LVAD_Counts_Data <- readxl**::read_xlsx**("../1_Input/1_Patient/Patient_Data.xlsx") **rownames**(LVAD_Counts_Data)<-LVAD_Counts_Data**$**Sample_ID
Index<-**as.data.frame**(dplyr**::select**(LVAD_Counts_Data, Ischemia, LV.EF, Age))
##PCA of raw Annotated Methlyation
All.t<-**t**(dplyr**::select**(DMR.raw, J**:**A))
PCA.All<-**prcomp**(All.t, scale=TRUE)
data.pca<-PCA.All**$**x
##merge the file
data.pca_Final<-**merge**(Index, data.pca, by=0) **rownames**(data.pca_Final)<-data.pca_Final**$**Row.names

pca.comp<-**prcomp**(data.pca_Final[,(**ncol**(Index)**+**1)**:ncol**(data.pca_Final)])

PCs<-**merge**(pca.comp**$**x, Index, by=0) **rownames**(PCs)<-PCs**$**Row.names
ax_text<-**list**(
 family = "times",
 size = 12,
 color = "black")
t <- **list**(
 family = "times",
 size = 16,
 color = "black")
p <- **plot_ly**(PCs, x = **~**PC1, y = **~**PC2, z = **~**PC3,
 marker = **list**(color = **~**Ischemia,
 colorscale = **c**('#FFE1A1', '#683531'),
 showscale = TRUE), text=**rownames**(PCs)) **%>%** **add_markers**() **%>%** **add_text**(textfont = t, textposition="bottom") **%>%** **layout**(scene = **list**(
 xaxis = **list**(title = 'PC1', zerolinewidth = 4,
 zerolinecolor="darkgrey", linecolor="darkgrey",
 linewidth=4, titlefont=t, tickfont=ax_text),
 yaxis = **list**(title = 'PC2', zerolinewidth = 4,
 zerolinecolor="darkgrey", linecolor="darkgrey",
 linewidth=4, titlefont=t, tickfont=ax_text),
 zaxis = **list**(title = 'PC3', zerolinewidth = 4,
 zerolinecolor="darkgrey", linecolor="darkgrey",
 linewidth=4, titlefont=t, tickfont=ax_text)),
 annotations = **list**(
 x = 1.13,
 y = 1.03,
 text = 'Ischemia',
 xref = '1',
 yref = '0',
 showarrow = FALSE,
 plot_bgcolor = 'black'))
p #must comment out for PDF generation via knitr (Pandoc)

Unsupervised Principal Components Analysis of DNA Methylation Data. The distribution of explained variance was wider for this dataset; however, an inflection of the variance curve is seen at PC3, therefore supporting the analysis of 3 principal components using a 3-dimensional plot.

pcaCharts=**function**(x) {
 x.var <- x**$**sdev **^** 2
 x.pvar <- x.var**/sum**(x.var)
 **par**(mfrow=**c**(2,2))
 **plot**(x.pvar,xlab="Principal component",
 ylab="Proportion of variance",
 ylim=**c**(0,1), type='b')
 **plot**(**cumsum**(x.pvar),xlab="Principal component",
 ylab="Cumulative Proportion of variance",
 ylim=**c**(0,1), type='b')
 **screeplot**(x)
 **screeplot**(x,type="l")
 **par**(mfrow=**c**(1,1))
} **pcaCharts**(pca.comp)

**
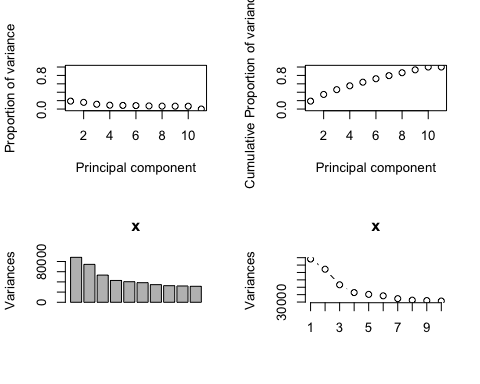
**

Figure S5. Unsupervised Principal Components Analysis of DNA Methylation.

Fig. 3B: Distribution of Methylation by Genomic and CpG Annotation.

The following figure illustrates the enrichment of differential methylation within CpG Islands (CpG-rich regions of the genome, GC% > 60% spanning more than 200 BPs) found within the associated gene promoter

library(plotly)
library(dplyr)
library(stringr)
library(reshape2)
library(readxl)
library(kableExtra)
paletteLength<-100
myColor <- colorRampPalette(c("dodgerblue4", "white", "brown4"))(paletteLength)
## Create a 3-dimensional Contour Plot
Contour_3D <- read_xlsx("../1_Input/3_Methylation/3D.Histogram_Transposed.xlsx")
rownames(Contour_3D)<-Contour_3D$Gene_Region
Contour_3D<-select(Contour_3D, `North Shelf`:`Open Sea`)
##Make a Table of the CpG Methylation Distribution
Contour_3D %>% kable( align="c", booktabs=T,
 caption="Methylation Distribution") %>%
 kable_styling(latex_options=c("striped", "condensed", "repeat_header"))

test<-readxl::read_xlsx("../1_Input/3_Methylation/3D.Histogram_Transposed.xlsx",
 sheet="Transposed")
test$CpG_Number<-as.numeric(test$CpG_Number)
Hist_3D<-data.matrix(Contour_3D)
rownames(Hist_3D)<-rownames(Contour_3D)
colnames(Hist_3D)<-colnames(Contour_3D)
color <- colorRampPalette(c("grey", "orange", "red"))
t <- list(
 family = "times",
 size = 16,
 color = "black")
q<-plot_ly(z=~Hist_3D, colors=color(10),
 text=as.character(rownames(Hist_3D))) %>% add_surface() %>%
 layout(scene = list(
 xaxis = list(title = 'CpG Region',
 type="category",
 zeroline=TRUE,
 showline=TRUE,
 zerolinewidth = 4,
 zerolinecolor="darkgrey",
 linecolor="darkgrey",
 linewidth=4,
 titlefont=t,
 tickfont=t),
 yaxis = list(title = 'Gene Region',
 zerolinewidth = 4,
 zerolinecolor="darkgrey",
 linecolor="darkgrey",
 linewidth=4,
 titlefont=t,
 tickfont=t),
 zaxis = list(title = 'DMCs',
 zerolinewidth = 4,
 zerolinecolor="darkgrey",
 linecolor="darkgrey",
 linewidth=4,
 titlefont=t,
 tickfont=t)))

q #must comment out for PDF generation via knitr (Pandoc).


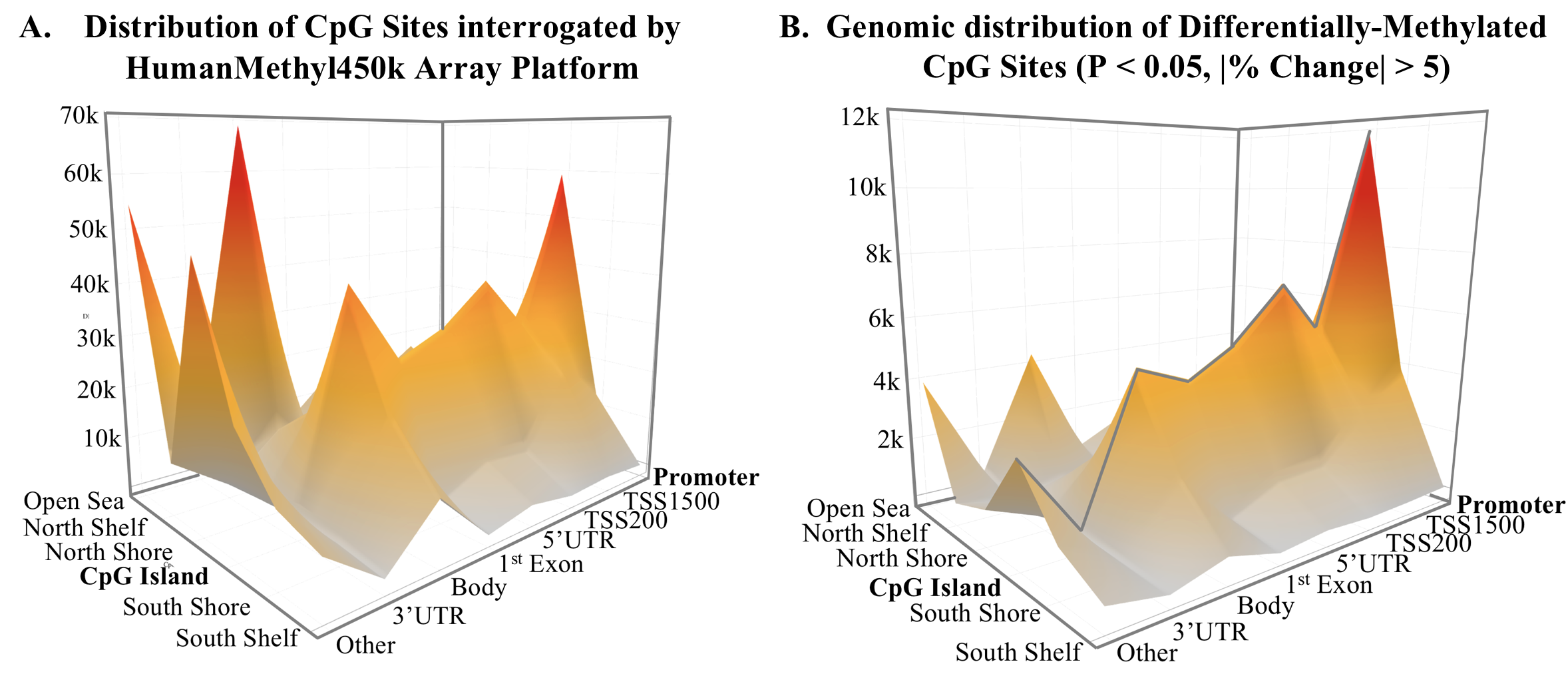


**Figure S6**: Distribution of CpG Sites. 3-dimensional contour plot depicting the distribution of DMCs about CpG and genomic regions for (A) All CpG sites interrogated by the HumanMethylation450k Array and (B) Differentially-methylated Cytosines (P<0.05 and |% Change|>5).

Fig. 3D: Heatmap and Hierarchical Clustering of Differential CpG Methylation (P < 0.05)

l**ibrary**(pheatmap) **library**(dplyr)
##Import Data Matrix
# DMR.raw <- read.csv("../1_Input/3_Methylation/LVAD_ICM.v.NICM_Methyl450k.csv")
## Filters to Apply to DMR
pvalue_threshold=0.05
DMP_location="Island"
Gene_region="Promoter_Associated"
##Filter Differential Methylation Data
DMR.p05<-DMR.raw **%>%** **filter**(pval**<**pvalue_threshold)
DMR.p05<-DMR.p05 **%>%** **select**(CpG_ProbeID,
 IF.vs..NF...Beta,
 pval,
 qval,
 Relation_to_Island,
 Regulatory_Feature_Group,
 chr,
 pos,
 J**:**A)
DMR.p05<-DMR.p05 **%>%** **filter**(**grepl**(DMP_location, Relation_to_Island))
DMR.p05.PromoterCGI<-DMR.p05 **%>%** **filter**(**grepl**(Gene_region,
 Regulatory_Feature_Group))
HM.ICM<-**data.matrix**(DMR.p05 **%>%** **select**(J**:**A))
#Import the Index File
LVAD_Counts_Data <- readxl**::read_xlsx**("../1_Input/1_Patient/Patient_Data.xlsx")
Index<-LVAD_Counts_Data **%>%** **select**(Ischemia, LV.EF, Age)
Index<-**as.data.frame**(Index) **rownames**(Index)<-LVAD_Counts_Data**$**Sample_ID
paletteLength <- 100
myColor <- **colorRampPalette**(**c**("dodgerblue4", "white", "brown4"))(paletteLength)

myBreaks <- **c**(**seq**(**min**(HM.ICM), 0, length.out=**ceiling**(paletteLength**/**2) **+** 1),
 **seq**(**max**(HM.ICM)**/**paletteLength,
 **max**(HM.ICM),
 length.out=**floor**(paletteLength**/**2)))
heatmap_DMC<-**pheatmap**(HM.ICM, scale="row",
 cluster_cols = TRUE,
 cluster_rows = TRUE,
 color = myColor,
 show_rownames = FALSE,
 border_color = NA,
 annotation_col = Index)

Fig. 4A: CpG Site Homology, De Novo Motif Discovery.

In order to determine whether the differentially-methylated positions are masking a specific list of upstream regulators, I took all of the promoter-associated CpG islands that are differentially methylated, and I looked within a 20 BP range of them for consensus sequences that are conserved within these transcriptionally-active DMRs. For this process, two approaches were necessary:

1. *De Novo* Motif Discovery
2. Known Motif Enrichment

To achieve (1) **De Novo Motif Discovery**, the following general steps were necessary:

1. Filter CpGs by specified criteria in R, exporting the BED-formatted loci.
2. Annotate this BED file using **H**ypergeometric **O**ptimization for **M**otif **E**n**R**ichment ([HOMER](http://homer.ucsd.edu/homer/index.html)) via annotatePearks.pl command.
3. Find *de novo* motifs using the findMotifs.pl command. HOMER offers a number of **Advantages** over other sequence alignment programs:

- CpG content correction (places loci into ‘bins’ based on CpG content, thereby unbiasing the motif discovery)
- **ZOOPS** (Zero Or One Per Sequence) Scoring coupled with hypergeometric enrichment calculations, comparing to background genomic sequences.
- Direct importation of genomic (.bed) sites (chromosome, chromStart, chromEnd, CpG_ID, [blank], strand).
- Uses customizable indexed FASTA file (hg19 in our case), along with Gene Ontology, relative gene position, etc… associated with most-proximal gene.

The specific workflow for *de novo* motif discovery were as follows:

1. CpG Filter:

- Promoter-Associated CpG Islands, P < 0.05

##Import the Dataset
# DMR.raw <- read.csv("../1_Input/3_Methylation/LVAD_ICM.v.NICM_Methyl450k.csv")
Inverse.DMR.DEG_p05<-**read.csv**("../1_Input/4_Combined/DEGp05_inverse_Promoter.CGIp05.csv")
## Filters to Apply to DMR
pvalue_threshold=0.05
DMP_location="Island"
Gene_region="Promoter_Associated"
RANGE=10
##Filter Differential Methylation Data
DMR.p05<-dplyr**::filter**(DMR.raw,
 pval**<**pvalue_threshold)
DMR.p05<-dplyr**::filter**(DMR.p05,
 **grepl**(DMP_location, Relation_to_Island))
DMR.p05<-dplyr**::select**(DMR.p05,
 CpG_ProbeID,
 IF.vs..NF...Beta,
 pval,
 qval,
 Relation_to_Island,
 Regulatory_Feature_Group,
 chr,
 pos,
 strand,
 J**:**A)
DMR.p05.PromoterCGI<-dplyr**::filter**(DMR.p05,
 **grepl**(Gene_region,
 Regulatory_Feature_Group)
 )
#prepare the HOMER input file (BED file format with first 6 columns)
BED<-dplyr**::select**(DMR.p05,
 chrom=chr,
 chromStart=pos,
 strand,
 CpG_ProbeID=CpG_ProbeID)
BED<-dplyr**::mutate**(BED, end=chromStart**+**RANGE)
BED<-dplyr**::mutate**(BED, start=chromStart**-**RANGE)
BED<-dplyr**::select**(BED,
 chrom,
 start,
 end,
 strand,
 CpG_ProbeID)
BED**$**space<-""
BED<-dplyr**::select**(BED,
 chrom,
 start,
 end,
 CpG_ProbeID ,
 space,
 strand)
##Convert stranded information to factor (+ = 1; - = 0)
BED**$**strand<-**as.numeric**(BED**$**strand)
BED**$**strand[BED**$**strand**==**"2"]<-0
#Write table that will serve as input for the HOMER analysis **library**(GenomicRanges) **library**(rtracklayer) **write.table**(BED,
 file=**paste0**("../2_Output/3_Methylation/Sequence.Homology/DMR.10BP_Range.txt"),
 quote=F,
 sep="\t",
 row.names = F,
 col.names = F)
gr <- GenomicRanges**::GRanges**(seqnames = **Rle**(BED**$**chrom),
 ranges = **IRanges**(BED**$**start, end = BED**$**end, names = BED**$**CpG_ProbeID))
df <- **data.frame**(seqnames=**seqnames**(gr),
 starts=**start**(gr)**-**1,
 ends=**end**(gr),
 names=**names**(gr)) **write.table**(df, file=**paste0**("../2_Output/3_Methylation/Sequence.Homology/",
 RANGE,".bed"),
 quote=F,
 sep="\t",
 row.names=F,
 col.names=F) **library**(kableExtra)
BED[1**:**10,] **%>%** **kable**( align="c", booktabs=T,
 caption="Example HOMER input .BED File") **%>%**
 **kable_styling**(latex_options=**c**("striped", "condensed", "repeat_header"))

1. Annotate BED file via HOMER:

annotatePeaks.pl DMR.p05_40BP.txt hg19 -size 20 -hist 20 -annStats stats.txt > output.txt

1. *De Novo* Motif Discovery via HOMER:

findMotifsGenome.pl DMR.p05_40BP.txt hg19 Folder_Output/ -size 50 -mask

Fig. 4B: Known Motif Enrichment.

TOMTOM is useful to compare the de novo motifs that were generated with the differential methylation dataset.

tomtom -no-ssc -oc . -verbosity 1 -min-overlap 5 -dist pearson -evalue -thresh 10.0 -xalph query_motifs db/HUMAN/HOCOMOCOv11_full_HUMAN_mono_meme_format.meme db/MOUSE/HOCOMOCOv11_full_MOUSE_mono_meme_format.meme 10 both

##Import the Methylation Annotations **library**(IlluminaHumanMethylation450kanno.ilmn12.hg19) **library**(GenomicRanges) **library**(rtracklayer)
##Import the Dataset
# DMR.raw <- read.csv("../1_Input/3_Methylation/LVAD_ICM.v.NICM_Methyl450k.csv")
## Filters to Apply to DMR
pvalue_threshold=0.05
DMP_location="Island"
Gene_region="Promoter_Associated"
##Filter Differential Methylation Data
DMR.p05<-dplyr**::filter**(DMR.raw,
 pval**<**pvalue_threshold)
DMR.p05<-dplyr**::filter**(DMR.p05,
 **grepl**(DMP_location,
 Relation_to_Island)
 )
DMR.p05<-dplyr**::select**(DMR.p05,
 CpG_ProbeID,
 IF.vs..NF...Beta,
 pval,
 qval,
 Relation_to_Island,
 Regulatory_Feature_Group,
 chr,
 pos,
 strand,
 J**:**A)
DMR.p05.PromoterCGI<-dplyr**::filter**(DMR.p05,
 **grepl**(Gene_region,
 Regulatory_Feature_Group)
 )
#prepare the HOMER input file (BED file format with first 6 columns)
BED<-dplyr**::select**(DMR.p05,
 chrom=chr,
 chromStart=pos,
 strand,
 CpG_ProbeID=CpG_ProbeID)
BED<-dplyr**::mutate**(BED, end=chromStart**+**1)
BED<-dplyr**::mutate**(BED, start=chromStart)
BED<-dplyr**::select**(BED,
 chrom,
 start,
 end,
 strand,
 CpG_ProbeID)
BED**$**space<-""
BED<-dplyr**::select**(BED,
 chrom,
 start,
 end,
 CpG_ProbeID,
 space,
 strand)
##Convert stranded information to factor (+ = 1; - = 0)
BED**$**strand<-**as.numeric**(BED**$**strand)
BED**$**strand[BED**$**strand**==**"2"]<-0
#Write table that will serve as input for the HOMER analysis **write.table**(BED, file="../2_Output/3_Methylation/Sequence.Homology/DMR.p05_40BP.txt",
 quote=F,
 sep="\t",
 row.names = F,
 col.names = F)

Fig. 5A: Correlation between DEGs and DMCs – DMC vs. DEG for NICM Patients (n = 6).

**library**(ggplot2) **library**(gridExtra) **library**(ggpubr) **library**(readxl)
Inverse.DMR.DEG_p05<-**read_xlsx**("../3_Results/Patientwise_ScatterPlot.xlsx",
 sheet="Summary - Scatter")
NICM_A<-dplyr**::select**(Inverse.DMR.DEG_p05,
 GeneSymbol,
 DMR=A_DMR,
 DEG=A_DEG)
NICM_A<-dplyr**::mutate**(NICM_A,
 Sample_ID="A")
NICM_B<-dplyr**::select**(Inverse.DMR.DEG_p05,
 GeneSymbol,
 DMR=B_DMR,
 DEG=B_DEG)
NICM_B<-dplyr**::mutate**(NICM_B,
 Sample_ID="B")
NICM_D<-dplyr**::select**(Inverse.DMR.DEG_p05,
 GeneSymbol,
 DMR=D_DMR,
 DEG=D_DEG)
NICM_D<-dplyr**::mutate**(NICM_D,
 Sample_ID="D")
NICM_F<-dplyr**::select**(Inverse.DMR.DEG_p05,
 GeneSymbol,
 DMR=F_DMR,
 DEG=F_DEG)
NICM_F<-dplyr**::mutate**(NICM_F,
 Sample_ID="F")
NICM_G<-dplyr**::select**(Inverse.DMR.DEG_p05,
 GeneSymbol,
 DMR=G_DMR,
 DEG=G_DEG)
NICM_G<-dplyr**::mutate**(NICM_G,
 Sample_ID="G")
NICM_H<-dplyr**::select**(Inverse.DMR.DEG_p05,
 GeneSymbol,
 DMR=H_DMR,
 DEG=H_DEG)
NICM_H<-dplyr**::mutate**(NICM_H,
 Sample_ID="H")
NICM<-**rbind**(NICM_A, NICM_B)
NICM<-**rbind**(NICM, NICM_D)
NICM<-**rbind**(NICM, NICM_F)
NICM<-**rbind**(NICM, NICM_G)
NICM<-**rbind**(NICM, NICM_H)
NICM<-dplyr**::mutate**(NICM, Etiology="NICM")
# scatter plot of x and y variables
# color by groups
scatterPlot <- **ggplot**(NICM,**aes**(DMR, DEG)) **+**
 **geom_point**(alpha=0.5) **+** **theme**(text=**element_text**(size=10, family="Times"))
# Marginal density plot of x (top panel)
xdensity <- **ggplot**(NICM, **aes**(DMR, fill=Sample_ID)) **+**
 **geom_density**(alpha=.5) **+** **theme**(text=**element_text**(size=10, family="Times"))
# Marginal density plot of y (right panel)
ydensity <- **ggplot**(NICM, **aes**(DEG, fill=Sample_ID)) **+**
 **geom_density**(alpha=.5) **+** **theme**(text=**element_text**(size=10, family="Times")) **ggarrange**(scatterPlot, xdensity**+rremove**("legend"), ydensity,
 labels=**c**("A", "B", "C"),
 ncol=2, nrow=2,
 font.label = **list**(size=12, face="bold", family="Times"))


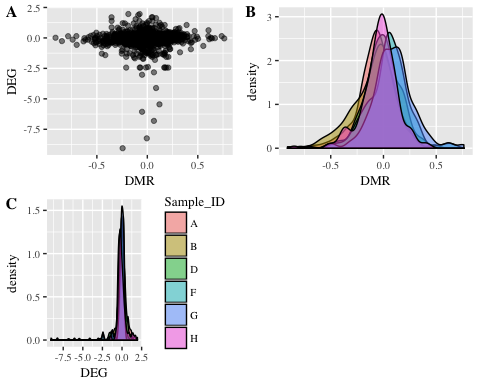


Figure S7. DEGs (P<0.05) containing promoter-associated inversely changing DMRs (P<0.05) were plotted by left ventricle samples obtained from non-ischemic cardiomyopathy(NICM) subjects.

Fig. 5B: Correlation between DEGs and DMCs – DMC vs. DEG for ICM Patients (n = 5).

###ICM Plots (identical to the NICM
# except with patients having known ischemic heart disease)
ICM_C<-dplyr**::select**(Inverse.DMR.DEG_p05,
 GeneSymbol, DMR=C_DMR,DEG=C_DEG)
ICM_C<-dplyr**::mutate**(ICM_C, Sample_ID="C")
ICM_E<-dplyr**::select**(Inverse.DMR.DEG_p05,
 GeneSymbol, DMR=E_DMR, DEG=E_DEG)
ICM_E<-dplyr**::mutate**(ICM_E, Sample_ID="E")
ICM_I<-dplyr**::select**(Inverse.DMR.DEG_p05,
 GeneSymbol, DMR=I_DMR, DEG=I_DEG)
ICM_I<-dplyr**::mutate**(ICM_I, Sample_ID="I")
ICM_J<-dplyr**::select**(Inverse.DMR.DEG_p05,
 GeneSymbol, DMR=J_DMR, DEG=J_DEG)
ICM_J<-dplyr**::mutate**(ICM_J, Sample_ID="J")
ICM_K<-dplyr**::select**(Inverse.DMR.DEG_p05,
 GeneSymbol, DMR=K_DMR, DEG=K_DEG)
ICM_K<-dplyr**::mutate**(ICM_K, Sample_ID="K")
ICM<-**rbind**(ICM_C, ICM_E)
ICM<-**rbind**(ICM, ICM_I)
ICM<-**rbind**(ICM, ICM_J)
ICM<-**rbind**(ICM, ICM_K)
ICM<-dplyr**::mutate**(ICM, Etiology="ICM")
# scatter plot of x and y variables

# color by groups
scatterPlot <- **ggplot**(ICM,**aes**(DMR, DEG, color=Etiology)) **+**
 **geom_point**(alpha=0.5) **+** **scale_color_brewer**(palette="Set1") **+** **theme**(text=**element_text**(size=10, family="Times"))
# Marginal density plot of x (top panel)
xdensity <- **ggplot**(ICM, **aes**(DMR, fill=Sample_ID)) **+**
 **geom_density**(alpha=.5) **+** **scale_color_brewer**(palette="Set1") **+** **theme**(text=**element_text**(size=10, family="Times"))
# Marginal density plot of y (right panel)
ydensity <- **ggplot**(ICM, **aes**(DEG, fill=Sample_ID)) **+**
 **geom_density**(alpha=.5) **+** **scale_color_brewer**(palette="Set1") **+** **theme**(text=**element_text**(size=10, family="Times")) **ggarrange**(scatterPlot**+rremove**("legend"),
 xdensity**+rremove**("legend"),
 ydensity,
 labels=**c**("A", "B", "C"),
 ncol=2, nrow=2,
 font.label = **list**(size=12, face="bold", family="Times"))


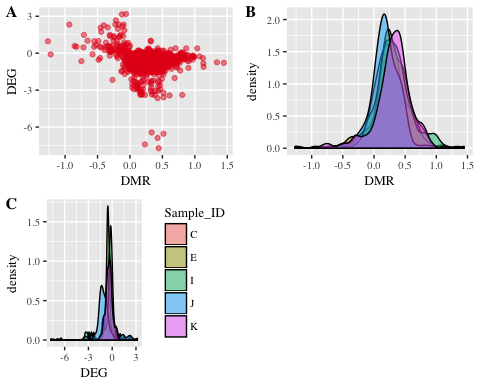


Figure S8. DEGs (P<0.05) containing promoter-associated inversely changing DMRs (P<0.05) were plotted by left ventricle samples obtained from ischemic cardiomyopathy(NICM) subjects.

Fig. 6: Combined DMC vs. DEG for both NICM and ICM Subjects.

##Combine NICM and ICM to create a synthesized output
LVAD_Total<-**rbind**(NICM, ICM)
*# color by groups*
scatterPlot <- **ggplot**(LVAD_Total,**aes**(DMR, DEG, color=Etiology)) **+**
 **geom_point**(alpha=0.5) **+** **scale_color_brewer**(palette="Set1") **+** **theme**(text=**element_text**(size=10, family="Times"))
*# Marginal density plot of x (top panel)*
xdensity <- **ggplot**(LVAD_Total, **aes**(DMR, fill=Etiology)) **+**
 **geom_density**(alpha=.5) **+** **scale_color_brewer**(palette="Set1") **+** **theme**(text=**element_text**(size=10, family="Times"))

*# Marginal density plot of y (right panel)*
ydensity <- **ggplot**(LVAD_Total, **aes**(DEG, fill=Etiology)) **+**
 **geom_density**(alpha=.5) **+** **scale_color_brewer**(palette="Set1") **+** **theme**(text=**element_text**(size=10, family="Times")) **ggarrange**(scatterPlot, xdensity**+rremove**("legend"), ydensity,
 labels=**c**("A", "B", "C"),
 ncol=1, nrow=3,
 font.label = **list**(size=12, face="bold", family="Times"))


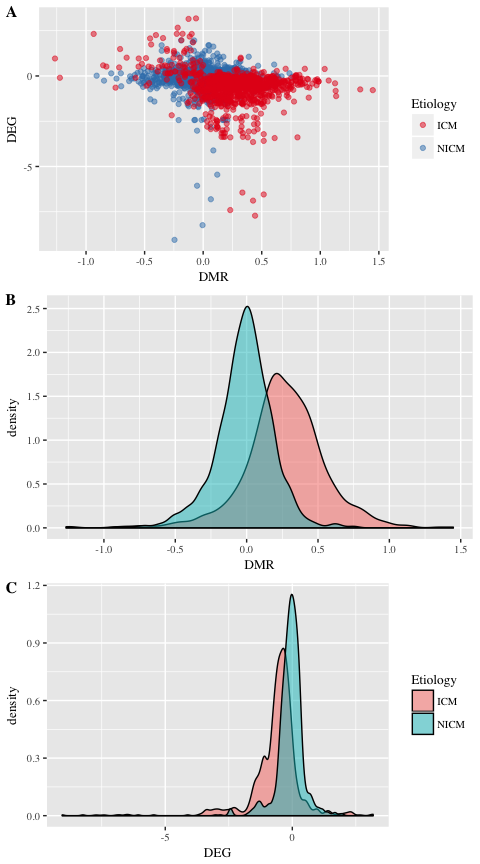


Figure S9. DEGs (P<0.05) containing promoter-associated inversely changing DMRs (P<0.05) were plotted by left ventricle samples obtained from all cardiomyopathy(NICM) subjects.

Fig. 5C: Scatter Plot of KLF15 Promoter Methylation with Inverse Gene Expression

###### Scatter Plot with marginals #######
library(plotly)
library(dplyr)
library(ggplot2)
library(ggpubr)
Inverse.DMR.DEG_p05<-read.csv("../1_Input/4_Combined/DEGp05_inverse_Promoter.CGIp05.csv")
Inverse.DMR.DEG_p05$GeneSymbol<-make.unique(as.character(Inverse.DMR.DEG_p05$GeneSymbol),
 sep="_")
rownames(Inverse.DMR.DEG_p05)<-Inverse.DMR.DEG_p05$GeneSymbol
###Adjust formatting to evaluate individual genes (use the inverse changes list)##
rownames_DMR<-colnames(select(Inverse.DMR.DEG_p05, J_DMR:A_DMR))
DMRs<-select(Inverse.DMR.DEG_p05, J_DMR:A_DMR)
## Standardize the Methylation by the Non-ischemic Subjects and convert to Percentage
DMR_standard<-DMRs %>%
 rowwise() %>%
 dplyr::mutate(Ave=mean(c(G_DMR,
 F_DMR,
 D_DMR,
 B_DMR,
 H_DMR,
 A_DMR),
 na.rm=TRUE))
DMR_standard<-DMR_standard %>% mutate("J"=(100*((J_DMR/Ave)-1)))
DMR_standard<-DMR_standard %>% mutate("K"=(100*((K_DMR/Ave)-1)))
DMR_standard<-DMR_standard %>% mutate("C"=(100*((C_DMR/Ave)-1)))
DMR_standard<-DMR_standard %>% mutate("E"=(100*((E_DMR/Ave)-1)))
DMR_standard<-DMR_standard %>% mutate("I"=(100*((I_DMR/Ave)-1)))
DMR_standard<-DMR_standard %>% mutate("G"=(100*((G_DMR/Ave)-1)))
DMR_standard<-DMR_standard %>% mutate("F"=(100*((F_DMR/Ave)-1)))
DMR_standard<-DMR_standard %>% mutate("D"=(100*((D_DMR/Ave)-1)))
DMR_standard<-DMR_standard %>% mutate("B"=(100*((B_DMR/Ave)-1)))
DMR_standard<-DMR_standard %>% mutate("H"=(100*((H_DMR/Ave)-1)))
DMR_standard<-DMR_standard %>% mutate("A"=(100*((A_DMR/Ave)-1)))
DMR_standard<-dplyr::select(DMR_standard, J:A)
rownames(DMR_standard)<-Inverse.DMR.DEG_p05$GeneSymbol
DMRs_t<-t(DMR_standard) #transpose data
colnames(DMRs_t)<-paste0(rownames(DMR_standard), "_DMR")
rownames(DMRs_t)<-sub("_DMR", "", colnames(DMRs))
## Differentially Expressed Genes
DEGs<-select(Inverse.DMR.DEG_p05, J_DEG:A_DEG)
rownames(DEGs)<-Inverse.DMR.DEG_p05$GeneSymbol
DEG_standard<-DEGs %>% rowwise() %>% dplyr::mutate(Ave=mean(c(G_DEG,
 G_DEG,
 D_DEG,
 B_DEG,
 H_DEG,
 A_DEG),
 na.rm=TRUE))
DEG_standard<-DEG_standard %>% mutate("J"=(log2(J_DEG/Ave)))
DEG_standard<-DEG_standard %>% mutate("K"=(log2(K_DEG/Ave)))
DEG_standard<-DEG_standard %>% mutate("C"=(log2(C_DEG/Ave)))
DEG_standard<-DEG_standard %>% mutate("E"=(log2(E_DEG/Ave)))
DEG_standard<-DEG_standard %>% mutate("I"=(log2(I_DEG/Ave)))
DEG_standard<-DEG_standard %>% mutate("G"=(log2(G_DEG/Ave)))
DEG_standard<-DEG_standard %>% mutate("F"=(log2(F_DEG/Ave)))
DEG_standard<-DEG_standard %>% mutate("D"=(log2(D_DEG/Ave)))
DEG_standard<-DEG_standard %>% mutate("B"=(log2(B_DEG/Ave)))
DEG_standard<-DEG_standard %>% mutate("H"=(log2(H_DEG/Ave)))
DEG_standard<-DEG_standard %>% mutate("A"=(log2(A_DEG/Ave)))
DEG_standard<-dplyr::select(DEG_standard, J:A)
rownames(DEG_standard)<-Inverse.DMR.DEG_p05$GeneSymbol
DEGs_t<-t(DEG_standard) #transpose data
colnames(DEGs_t)<-paste0(rownames(DEG_standard), "_DEG")
rownames(DEGs_t)<-sub("_DEG", "", colnames(DEGs))
##

#Import Index
Index<- readxl::read_xlsx("../1_Input/1_Patient/Patient_Data.xlsx")
rownames(Index)<-Index$Sample_ID
Scatter_Data<-merge(DMRs_t, DEGs_t, by=0)
Scatter_Data<-as.data.frame(Scatter_Data)
Scatter_Data<-merge(Index, Scatter_Data, by.x="Sample_ID", by.y="Row.names")
Scatter_Data$Etiology<-factor(Scatter_Data$Ischemia)

##Choose a Gene from within the DMR.DEG_inverse dataset
DMRs_list<-select(Scatter_Data, "KLF15_DMR",
 "KLF15_DEG", Ischemia)
t <- list(
 family = "times",
 size = 16,
 color = "black")
xax <- list(
 zeroline = TRUE,
 showline = TRUE,
 mirror = "ticks",
 gridcolor = toRGB("gray50"),
 gridwidth = 2,
 zerolinecolor = toRGB("black"),
 zerolinewidth = 4,
 linecolor = toRGB("black"),
 linewidth = 6,
 titlefont=t,
 tickfont=t,
 title="Log2(Percent Methylation)"
)
yax <- list(
 zeroline = TRUE,
 showline = TRUE,
 mirror = "ticks",
 gridcolor = toRGB("gray50"),
 gridwidth = 2,
 zerolinecolor = toRGB("black"),
 zerolinewidth = 4,
 linecolor = toRGB("black"),
 linewidth = 6,
 titlefont=t,
 tickfont=t,
 title="Log2(Fold-Change)"
)
KLF15_plot<-plot_ly(Scatter_Data, x = ~KLF15_DMR, y = ~KLF15_DEG,
 type="scatter",
 text = paste("Patient:", Scatter_Data$Sample_ID,
 ", Ischemic:",
 Scatter_Data$Ischemia,
 ", Age: ",
 Scatter_Data$Age),
 mode = "markers",
 color = ~Ischemia,
 colors = c("dodgerblue4", "firebrick4"),
 marker=list(size=20, opacity=0.8)
 ) %>%
 layout(xaxis=xax, yaxis=yax)
KLF15_plot

Scatter Plot of DEG (Q<0.05, |Fold-Change| > 1.5, FPKM > 2) vs. promoter-associated DMP (P<0.05, |Percent Change| > 10%) for the KLF15 gene across all LVAD subjects.

regression<-lm(KLF15_DEG~KLF15_DMR, data=DMRs_list)
summary(regression)

##
## Call:
## lm(formula = KLF15_DEG ~ KLF15_DMR, data = DMRs_list)
##
## Residuals:
## Min 1Q Median 3Q Max
## -2.26561 -0.71502 -0.04725 1.05967 1.42713
##
## Coefficients:
## Estimate Std. Error t value Pr(>|t|)
## (Intercept) -0.57127 0.46745 -1.222 0.253
## KLF15_DMR -0.01881 0.02002 -0.939 0.372
##
## Residual standard error: 1.228 on 9 degrees of freedom
## Multiple R-squared: 0.08931, Adjusted R-squared: -0.01187
## F-statistic: 0.8827 on 1 and 9 DF, p-value: 0.372

Fig. 5D Bubble Plot and Gene Density of KLF15 Differential Methylation

We next wanted to determine whether the top differentially-methylated positions represented larger regions of enhanced methylation change. To test for this, a bubble plot was created for downstream targets of EZH2 and top candidate regulators KLF15. Percent Methylation of all CpG sites interrogated by the HumanMethyl450k array were plotted by genomic locus, with the bubble size proportional to -Log_10_(P Value).

**library**(plotly)
########### KLF15
KLF15<-readxl**::read_xlsx**("../3_Results/KLF_CpGs.xlsx",
 sheet="KLF15-Scatter")
*# Make scatter plot based on ALL CpG sites associated with gene:*
KLF15_plot<-**plot_ly**(KLF15,
 x = **~**CpG_Position_Chr3,
 y = **~**Percent.Methylation,
 type="scatter",
 text = **paste**("CpG Site -", KLF15**$**CpG_ProbeID,
 ", % Methylation -",
 KLF15**$**Percent.Methylation,
 ", p-value -",
 KLF15**$**pval),
 mode = "markers", color = **~**`-log(p)`, size = **~**`-log(p)`)
KLF15_plot

Density plot illustrating the distribution and differential methylation of CpG Sites associated with the KLF15 gene, regardless of statistical significance.

KLF15_density<-**ggplot**(KLF15, **aes**(CpG_Position_Chr3,
 fill = Percent.Methylation)) **+** **geom_density**(fill = "#ff4d4d", alpha = 0.2)
KLF15_density

Fig. 6A: Merging RNA Sequencing with Inverse Differential DNA Methylation

The next task was to determine whether the differentially methylated regions with coordinated gene expression associated with either genomic regions or known transcriptional pathways.

##Create the inversely changing Genes and DMRs (Venn Diagram Adaptation) **library**(dplyr) **library**(data.table) **library**(readxl)
#Import the DEG and DMR
# DMR.raw <- read.csv("../1_Input/3_Methylation/LVAD_ICM.v.NICM_Methyl450k.csv")
DEGs <- **read.csv**("../1_Input/2_RNA/ICM.v.NICM_RNA.csv")
DEG_ncounts<-**read_xlsx**("../1_Input/2_RNA/Counts_RNA.xlsx",
 sheet="Normalized Counts")
DEG_raw<-**left_join**(DEGs, DEG_ncounts, by="gene_id")
## Filters to Apply
pvalue_threshold=0.05
DMP_location="Island"
Gene_region="Promoter_Associated"
##Filter Differential Methylation Data
DMR.p05<-**filter**(DMR.raw, pval**<**pvalue_threshold)
DMR.p05<-dplyr**::select**(DMR.p05, CpG_ProbeID,
 perc.change=IF.vs..NF...Beta, pval, qval,
 Relation_to_Island,
 Regulatory_Feature_Group,
 chrom=chr, chromStart=pos, strand, J**:**A)
DMR.p05<-**filter**(DMR.p05, **grepl**(DMP_location, Relation_to_Island))
DMR.p05.PromoterCGI<-dplyr**::filter**(DMR.p05,
 **grepl**(Gene_region, Regulatory_Feature_Group)) **colnames**(DMR.p05.PromoterCGI)<-**paste0**(**colnames**(DMR.p05.PromoterCGI), "_DMR")

##Filter RNA Expression
DEG.p05<-**filter**(DEG_raw, p_value**<**pvalue_threshold)
DEG.p05<-**data.table**(DEG.p05)
DEG.p05**$**gene<-**as.character**(DEG.p05**$**gene)
DEG.p05.separated<-DEG.p05[, **strsplit**(gene, ',', fixed=T), by=test_id]
DEG.p05.separated<-**distinct**(DEG.p05.separated)
DEG.p05.final<-**merge**(DEG.p05.separated, DEG.p05, by="test_id")
DEG.p05.final**$**FoldChange<-**as.numeric**(**as.character**(DEG.p05.final**$**FoldChange))
DEG.p05.final**$**log2.fold_change.<-**as.numeric**(**as.character**(DEG.p05.final**$**log2.fold_change.))
#
##Annotate the RNA Data with CpG_ProbeIDs that match
UCSC_Gene_Ref<-**as.character**(DMR.raw**$**UCSC_RefGene_Name)
reference.table<-dplyr**::select**(DMR.raw, CpG_ProbeID)
reference.table<-**data.table**(reference.table)
reference.table<-**cbind**(reference.table, UCSC_Gene_Ref)
reference<-reference.table[, **strsplit**(UCSC_Gene_Ref, ';', fixed=T), by=CpG_ProbeID]
reference<-**distinct**(reference)

DEG.p05.Annotated<-dplyr**::left_join**(DEG.p05.final, reference, by="V1")
DEG.p05.Annotated<-**distinct**(DEG.p05.Annotated) **colnames**(DEG.p05.Annotated)<-**paste0**(**colnames**(DEG.p05.Annotated), "_DEG")
#Merge the two datasets
DMR.DEG_p05<-**merge**(DEG.p05.Annotated,
 DMR.p05.PromoterCGI,
 by.x='CpG_ProbeID_DEG',
 by.y="CpG_ProbeID_DMR")
DMR.DEG_p05<-dplyr**::select**(DMR.DEG_p05,
 GeneSymbol=V1_DEG,
 CpG_ProbeID_DEG,
 FoldChange_DEG,
 p_value_DEG,
 q_value_DEG,
 perc.change_DMR,
 pval_DMR,
 chrom_DMR,
 chromStart_DMR,
 strand_DMR,
 J_DEG**:**A_DEG,
 J_DMR**:**A_DMR)
DMR.DEG_p05**$**FoldChange.DEG<-**as.numeric**(**as.character**(DMR.DEG_p05**$**FoldChange_DEG))

#Export the large dataset **write.csv**(DMR.DEG_p05, "../1_Input/4_Combined/DMR.DEG_Merged at P<0.05.csv")

# filter for only the inversely changing DMRs with DEGs
Inverse.DMR.DEG_p05<-**filter**(DMR.DEG_p05,
 (FoldChange_DEG**>**0 **&** perc.change_DMR**<**0) **|** (FoldChange_DEG**<**0 **&** perc.change_DMR**>**0))
Inverse.DMR.DEG_p05<-dplyr**::mutate**(Inverse.DMR.DEG_p05,
 chromEnd_DMR=chromStart_DMR**+**1)
Inverse.UP.DMR.DEG_p05<-dplyr**::filter**(DMR.DEG_p05,
 FoldChange_DEG**>**0 **&** perc.change_DMR**<**0)
Inverse.DOWN.DMR.DEG_p05<-dplyr**::filter**(DMR.DEG_p05,
 FoldChange_DEG**<**0 **&** perc.change_DMR**>**0)
Both.UP<-dplyr**::filter**(Inverse.DOWN.DMR.DEG_p05,
 FoldChange_DEG**>**0, perc.change_DMR**>**0)
Both.DOWN<-dplyr**::filter**(Inverse.DOWN.DMR.DEG_p05,
 FoldChange_DEG**<**0, perc.change_DMR**<**0) **write.csv**(Both.UP, "../1_Input/4_Combined/DMR.DEG_Both UP_p<0.05.csv", row.names=FALSE) **write.csv**(Both.DOWN, "../1_Input/4_Combined/DMR.DEG_Both Down_P<0.05.csv", row.names=FALSE) **write.csv**(Inverse.DMR.DEG_p05, "../1_Input/4_Combined/DEGp05_inverse_Promoter.CGIp05.csv",
 row.names=FALSE)

#Identify the Non-overlapping Genes and DMRs
DEG.ONLY.List<-**setdiff**(DEG.p05.Annotated**$**CpG_ProbeID,
 DMR.p05.PromoterCGI**$**CpG_ProbeID)
DMR.ONLY.List<-**setdiff**(DMR.p05.PromoterCGI**$**CpG_ProbeID,
 DEG.p05.Annotated**$**CpG_ProbeID)
##Determine the Genes that do NOT have DMRs
test.RNA<-**cbind**(DEG.ONLY.List, 1)
test.RNA<-**as.data.frame**(test.RNA)
test.RNA<-**distinct**(test.RNA)
test.RNA.2<-**merge**(test.RNA,
 DEG.p05.Annotated,
 by.x="DEG.ONLY.List",
 by.y="CpG_ProbeID_DEG")
test.RNA.3<-**select**(test.RNA.2, V1_DEG, FoldChange_DEG)
test.RNA.4<-**distinct**(test.RNA.3)
DEG.ONLY.UP.COUNT<-**filter**(test.RNA.4, FoldChange_DEG**>**0)
DEG.ONLY.DOWN.COUNT<-**filter**(test.RNA.4, FoldChange_DEG**<**0)
# Determine the DMRs that annotate to genes that are NOT differentially expressed
test.DMR<-**cbind**(DMR.ONLY.List, 1)
test.DMR<-**as.data.frame**(test.DMR)
test.DMR<-**distinct**(test.DMR)
test.DMR.2<-**merge**(test.DMR, DMR.p05.PromoterCGI, by.x="DMR.ONLY.List", by.y="CpG_ProbeID_DMR")
test.DMR.3<-**select**(test.DMR.2, DMR.ONLY.List, perc.change_DMR)
test.DMR.4<-**distinct**(test.DMR.3)
DMR.ONLY.UP.COUNT<-**filter**(test.DMR.4, perc.change_DMR**>**0)
DMR.ONLY.DOWN.COUNT<-**filter**(test.DMR.4, perc.change_DMR**<**0)
# Inversely Expressed Gene Labels
Gene_Labels<-**select**(Inverse.DMR.DEG_p05, chrom=chrom_DMR,
 chromStart=chromStart_DMR, chromEnd=chromEnd_DMR, GeneSymbol)
Gene_Labels<-**arrange**(Gene_Labels, chromStart)
Gene_Labels**$**chrom<-**factor**(Gene_Labels**$**chrom, levels=**c**("chr1", "chr2", "chr3", "chr4",
 "chr5", "chr6", "chr7", "chr8",
 "chr9", "chr10", "chr11", "chr12",
 "chr13", "chr14", "chr15", "chr16",
 "chr17", "chr18", "chr19", "chr20",
 "chr21", "chr22", "chr23", "chrX",
 "chrY"))
Gene_Labels<-Gene_Labels[**order**(Gene_Labels**$**chrom),]
Gene_Labels<-Gene_Labels[**!duplicated**(Gene_Labels[,4]),]

q05<-**filter**(Inverse.DMR.DEG_p05, q_value_DEG**<**0.05)
Labels_q05<-**select**(q05, chrom=chrom_DMR,
 chromStart=chromStart_DMR, chromEnd=chromEnd_DMR, GeneSymbol)
Labels_q05<-**arrange**(Labels_q05, chromStart)
Labels_q05**$**chrom<-**factor**(Labels_q05**$**chrom, levels=**c**("chr1", "chr2", "chr3", "chr4",
 "chr5", "chr6", "chr7", "chr8",
 "chr9", "chr10", "chr11", "chr12",
 "chr13", "chr14", "chr15", "chr16",
 "chr17", "chr18", "chr19", "chr20",
 "chr21", "chr22", "chr23", "chrX",
 "chrY"))
Labels_q05<-Labels_q05[**order**(Labels_q05**$**chrom),]
Labels_q05<-Labels_q05[**!duplicated**(Labels_q05[,4]),]

#Fold Change UP (RNA)
Gene_FoldChange.UP<-Inverse.UP.DMR.DEG_p05 **%>%** **select**(chrom=chrom_DMR,
 chromStart=chromStart_DMR, FoldChange_DEG)
Gene_FoldChange.UP<-**mutate**(Gene_FoldChange.UP, chromEnd=chromStart**+**1)
Gene_FoldChange.UP<-Gene_FoldChange.UP **%>%** **select**(chrom, chromStart, chromEnd, FoldChange_DEG)
Gene_FoldChange.UP<-**arrange**(Gene_FoldChange.UP, chromStart)
Gene_FoldChange.UP**$**chrom<-**factor**(Gene_FoldChange.UP**$**chrom, levels=**c**("chr1", "chr2", "chr3", "chr4",
 "chr5", "chr6", "chr7", "chr8",
 "chr9", "chr10", "chr11", "chr12",
 "chr13", "chr14", "chr15", "chr16",
 "chr17", "chr18", "chr19", "chr20",
 "chr21", "chr22", "chr23", "chrX",
 "chrY"))
Gene_FoldChange.UP<-Gene_FoldChange.UP[**order**(Gene_FoldChange.UP**$**chrom),]
#Fold Change DOWN (RNA)
Gene_FoldChange.DOWN<-Inverse.DOWN.DMR.DEG_p05 **%>%** **select**(chrom=chrom_DMR,
 chromStart=chromStart_DMR, FoldChange_DEG)
Gene_FoldChange.DOWN<-**mutate**(Gene_FoldChange.DOWN, chromEnd=chromStart**+**1)
Gene_FoldChange.DOWN<-Gene_FoldChange.DOWN **%>%** **select**(chrom,
 chromStart, chromEnd, FoldChange_DEG)
Gene_FoldChange.DOWN<-**arrange**(Gene_FoldChange.DOWN, chromStart)
Gene_FoldChange.DOWN**$**chrom<-**factor**(Gene_FoldChange.DOWN**$**chrom, levels=**c**("chr1", "chr2", "chr3", "chr4",
 "chr5", "chr6", "chr7", "chr8",
 "chr9", "chr10", "chr11", "chr12",
 "chr13", "chr14", "chr15", "chr16",
 "chr17", "chr18", "chr19", "chr20",
 "chr21", "chr22", "chr23", "chrX",
 "chrY"))
Gene_FoldChange.DOWN<-Gene_FoldChange.DOWN[**order**(Gene_FoldChange.DOWN**$**chrom),]
##Fold Change List
Gene_FoldChange_List<-**list**(Gene_FoldChange.UP, Gene_FoldChange.DOWN) **library**(circlize) **library**(gtools) **library**(dplyr)
om = **circos.par**("track.margin")
oc = **circos.par**("cell.padding") **circos.par**(track.margin = **c**(0, 0), cell.padding = **c**(0, 0, 0, 0)) **circos.par**(start.degree = **-**250) **circos.initializeWithIdeogram**(track.height = 0.05)
### Labels for inversely changing DMRs with DEG **circos.genomicLabels**(Gene_Labels, labels.column=4, side='outside', cex=0.38)
## Add CpG Island Lines here (blocks to tell the reader where the CpG Islands are located?)
# Methylation Density
DMR.PerChange<-**select**(DMR.p05.PromoterCGI, chrom=chrom_DMR,
 chromStart=chromStart_DMR, perc.change=perc.change_DMR)
DMR.PerChange<-**mutate**(DMR.PerChange, chromEnd=chromStart**+**1)
DMR.PerChange<-**select**(DMR.PerChange, chrom, chromStart, chromEnd, perc.change)
DMR.PerChange**$**chrom<-**factor**(DMR.PerChange**$**chrom, levels=**c**("chr1", "chr2", "chr3", "chr4",
 "chr5", "chr6", "chr7", "chr8",
 "chr9", "chr10", "chr11", "chr12",
 "chr13", "chr14", "chr15", "chr16",
 "chr17", "chr18", "chr19", "chr20",
 "chr21", "chr22", "chr23", "chrX",
 "chrY"))
DMR.PerChange<-DMR.PerChange[**order**(DMR.PerChange**$**chrom),]
Methyl.UP<-**filter**(DMR.PerChange, perc.change**>**0)
Methyl.DOWN<-**filter**(DMR.PerChange, perc.change**<**0)
Methyl.List<-**list**(Methyl.UP, Methyl.DOWN) **circos.genomicDensity**(Methyl.List, col=**c**("#FF000080", "darkgreen"),
 track.height=0.1, bg.border=NA)
##DEG with inverse GPI Islands Promoters **circos.genomicTrackPlotRegion**(Gene_FoldChange_List,
 ylim = **c**(**-**4, 4), bg.border=NA,
 panel.fun = **function**(region, value, ...) {
 col = **ifelse**(value[[1]] **>** 0, "darkgoldenrod1", "blue")
 **circos.genomicPoints**(region, value, col = col, cex = 0.8, pch = 16)
 cell.xlim = **get.cell.meta.data**("cell.xlim")
 **for**(h **in** **c**(**-**4, **-**2, 0, 2, 4)) {
 **circos.lines**(cell.xlim, **c**(h, h), col ="#00000040")
 }
}, track.height = 0.1) **circos.par**(track.margin=om, cell.padding=oc)
## Add link for all DEGs with DMRs in promoter CGIs
Link_Anchor <- **read.csv**("../1_Input/4_Combined/Circos/Link_Anchor.csv")
Link<-**read.csv**("../1_Input/4_Combined/Circos/Link_DEG.DMR_Promoter.CGI_P<0.05.csv")
Link**$**chrom<-**factor**(Link**$**chrom, levels=**c**("chr1", "chr2", "chr3", "chr4",
 "chr5", "chr6", "chr7", "chr8",
 "chr9", "chr10", "chr11", "chr12",
 "chr13", "chr14", "chr15", "chr16",
 "chr17", "chr18", "chr19", "chr20",
 "chr21", "chr22", "chr23", "chrX",
 "chrY"))
Link<-Link[**order**(Link**$**chrom),]
Link_Anchor<-Link_Anchor[1**:nrow**(Link),] **circos.genomicLink**(Link, Link_Anchor, col="black", lwd=0.5)

Fig. 6B: Hierarchical Pathway Analysis of DEGs with Inverse DMCs.

Pathway Analysis was performed on the 124 genes inversely correlated with 211 DMPs using the **WEB**-based **G**ene **S**e**T** **A**naLysis **T**oolkit [WEBGestalt](http://www.webgestalt.org/option.php).

**library**(dplyr) **library**(readxl) **library**(pheatmap) **library**(RColorBrewer)
one <- **read_xlsx**("../1_Input/4_Combined/Webgestalt_Reactome.Analysis/Pathway.Clustering.Input.xlsx",
 sheet = "1")

two <- **read_xlsx**("../1_Input/4_Combined/Webgestalt_Reactome.Analysis/Pathway.Clustering.Input.xlsx",
 sheet = "2")

three <- **read_xlsx**("../1_Input/4_Combined/Webgestalt_Reactome.Analysis/Pathway.Clustering.Input.xlsx",
 sheet = "3")

four <- **read_xlsx**("../1_Input/4_Combined/Webgestalt_Reactome.Analysis/Pathway.Clustering.Input.xlsx",
 sheet = "4")

five <- **read_xlsx**("../1_Input/4_Combined/Webgestalt_Reactome.Analysis/Pathway.Clustering.Input.xlsx",
 sheet = "5")
##Combine all of the datasets
total<-**full_join**(one, two, by='GeneSymbol')
total<-**full_join**(total, three, by='GeneSymbol')
total<-**full_join**(total, four, by='GeneSymbol')
total<-**full_join**(total, five, by='GeneSymbol')
total<-**distinct**(total) **rownames**(total)<-total**$**GeneSymbol
total<-dplyr**::select**(total,
 `TCA Cycle`=FoldChange.DEG_1_TCA.Cycle,
 `electron transport`=FoldChange.DEG_2_electron.t,
 `Complex I`=FoldChange.DEG_3_ComplexI,
 `Pyruvate Metabolism`=FoldChange.DEG.4_Pyruvate,
 `Mitochondrial Fatty Acid Oxidation`=`FoldChange.DEG_5_Mtx FAO`)
cluster<-total[,2**:ncol**(total)]
cluster<-**data.matrix**(cluster)
cluster[**is.na**(cluster)]<-0 **rownames**(cluster)<-total**$**GeneSymbol

##row names to display
row.display<-one
test<-row.display**$**GeneSymbol

paletteLength <- 100
myColor <- **colorRampPalette**(**c**("dodgerblue4", "white", "gold2"))(paletteLength)
# length(breaks) == length(paletteLength) + 1
# use floor and ceiling to deal with even/odd length pallettelengths
myBreaks <- **c**(**min**(cluster),
 **seq**(**-**2,
 0,
 length.out=**ceiling**(paletteLength**/**2)),
 **seq**(2**/**paletteLength, 2,
 length.out=**floor**(paletteLength**/**2)**-**1),
 **max**(cluster)) **pheatmap**(cluster,
 cluster_cols=FALSE,
 border_color=NA,
 cluster_rows=TRUE,
 scale = 'none',
 show_colnames = T,
 show_rownames = T,
 color = myColor,
 breaks = myBreaks)

Fig. 7B: EZH2 as a Putative Nodal Regulator with DNA Methylation.

Based on the analysis of gene expression via RNA sequencing using (2016 [ENCODE](https://www.encodeproject.org) Consortium databased enrichment within [enrichr](http://amp.pharm.mssm.edu/Enrichr/), differential gene expression was found to enrich ChIP-Sequencing dataset for EZH2. Using this information, it was desired to determine whether the downsstream targets are hyper-methylated, as EZH2 induction would theorize. Therefore, the following analysis was performed to subset downstream targets according to relative methylation (P<0.05).

**library**(data.table) **library**(dplyr) **library**(readxl) **library**(RColorBrewer) **library**(pheatmap)
## Load Data for the T2_ND Methyl Analyis
DEG_raw <- **read.csv**("../1_Input/2_RNA/ICM.v.NICM_RNA.csv")
EZH2_targets <- **read_xlsx**("../1_Input/2_RNA/Enrichr/ENCODE_TF_ChIP-seq_2015_table.xlsx",
 sheet = "EZH2-Targets", col_names = FALSE)
# DMR.raw <- read.csv("../1_Input/3_Methylation/LVAD_ICM.v.NICM_Methyl450k.csv")

## Filters to Apply to DMR
pvalue_threshold=0.05
DMP_location="Island"
Gene_region="Promoter_Associated"
##Filter Differential Methylation Data
DMR.p05<-DMR.raw **%>%** **filter**(pval**<**pvalue_threshold)
DMR.p05<-DMR.p05 **%>%** **select**(CpG_ProbeID,
 IF.vs..NF...Beta,
 pval,
 qval,
 Relation_to_Island,
 Regulatory_Feature_Group,
 chr,
 pos,
 J**:**A)
DMR.p05<-DMR.p05 **%>%** **filter**(**grepl**(DMP_location, Relation_to_Island))
DMR.p05.PromoterCGI<-DMR.p05 **%>%** **filter**(**grepl**(Gene_region, Regulatory_Feature_Group))
#annotate the CpGs
UCSC_Gene_Ref<-**as.character**(DMR.raw**$**UCSC_RefGene_Name)
reference.table<-DMR.raw **%>%** **select**(CpG_ProbeID)
reference.table<-**data.table**(reference.table)
reference.table<-**cbind**(reference.table, UCSC_Gene_Ref)
reference<-reference.table[, **strsplit**(UCSC_Gene_Ref, ';', fixed=T), by=CpG_ProbeID]
reference<-**distinct**(reference)
DMR.p05.PromoterCGI<-**merge**(DMR.p05.PromoterCGI, reference, by="CpG_ProbeID")
DMR.p05.PromoterCGI<-DMR.p05.PromoterCGI **%>%** **select**(GeneSymbol=V1,
 CpG_ProbeID, perc.change=IF.vs..NF...Beta, pval_DMR=pval, chrom=chr, chromStart=pos)
DMR.p05.PromoterCGI<-DMR.p05.PromoterCGI **%>%** **mutate**(chromEnd=chromStart**+**1)

##Filter RNA Expression
DEG.p05<-**filter**(DEG_raw, q_value**<**pvalue_threshold)
DEG.p05<-**data.table**(DEG.p05)
DEG.p05**$**gene<-**as.character**(DEG.p05**$**gene)
DEG.p05.separated<-DEG.p05[, **strsplit**(gene, ',', fixed=T), by=test_id]
DEG.p05.separated<-**distinct**(DEG.p05.separated)
DEG.p05.final<-**merge**(DEG.p05.separated, DEG.p05, by="test_id")
DEG.p05.final**$**FoldChange<-**as.numeric**(**as.character**(DEG.p05.final**$**FoldChange))
DEG.p05.final**$**log2.fold_change.<-**as.numeric**(**as.character**(DEG.p05.final**$**log2.fold_change.))

#merge the two to determine DEGs targeted by EZH2
EZH2_DEGs<-**merge**(EZH2_targets, DEG.p05.final, by.x="X__1", by.y="gene")
EZH2_DMRs<-**merge**(EZH2_targets, DMR.p05.PromoterCGI, by.x="X__1", by.y="GeneSymbol")
#UP for DMR
EZH2_DMRs.UP<-EZH2_DMRs **%>%** **filter**(perc.change**>**0)
EZH2_DMRs.UP<-EZH2_DMRs.UP **%>%** **mutate**(color="red")
EZH2_DMR.Labels_UP<-EZH2_DMRs.UP **%>%** **select**(chrom, chromStart, chromEnd, color) **write.csv**(EZH2_DMR.Labels_UP, "../1_Input/5_EZH2/EZH2_DMR.Labels_UP.csv", row.names = FALSE)
#DOWN for DMR
EZH2_DMRs.DOWN<-EZH2_DMRs **%>%** **filter**(perc.change**<**0)
EZH2_DMRs.DOWN<-EZH2_DMRs.DOWN **%>%** **mutate**(color="blue")
EZH2_DMR.Labels_DOWN<-EZH2_DMRs.DOWN **%>%** **select**(chrom, chromStart, chromEnd, color) **write.csv**(EZH2_DMR.Labels_DOWN,"../1_Input/5_EZH2/EZH2_DMR.Labels_DOWN.csv", row.names = FALSE)
Gene_Labels<-EZH2_DMRs **%>%** **select**(chrom, chromStart, chromEnd, GeneSymbol=X__1) **write.csv**(Gene_Labels, "../1_Input/5_EZH2/EZH2_Gene.Labels.csv", row.names = FALSE)

#create the 'anchor' for the EZH2 gene
EZH2_location<-DMR.raw **%>%** **filter**(**grepl**("EZH2",
 UCSC_RefGene_Name) **&** **grepl**("TSS1500", UCSC_RefGene_Group) **&** **grepl**("S_Shore", Relation_to_Island))
EZH2_coordinates<-EZH2_location **%>%** **select**(chrom=chr, chromStart=pos)
EZH2_coordinates<-EZH2_coordinates **%>%** **mutate**(chromEnd=chromStart**+**1)
EZH2_Anchor<-EZH2_coordinates[**rep**(**seq_len**(**nrow**(EZH2_coordinates)),
 times=**nrow**(**rbind**(EZH2_DMR.Labels_UP, EZH2_DMR.Labels_DOWN))),] **write.csv**(EZH2_Anchor,"../1_Input/5_EZH2/EZH2_Anchor.csv", row.names = FALSE)

#merge together
EZH2_DEG.DMR<-**merge**(EZH2_DEGs, EZH2_DMRs, by="X__1") **library**(circlize) **library**(gtools) **library**(dplyr)

om = **circos.par**("track.margin")
oc = **circos.par**("cell.padding") **circos.par**(track.margin = **c**(0, 0), cell.padding = **c**(0, 0, 0, 0)) **circos.par**(start.degree = **-**70) **circos.initializeWithIdeogram**(plotType = NULL)

##EZH2
###labels
Gene_Labels_EZH2<-**read.csv**("../1_Input/5_EZH2/EZH2_Gene.Labels.csv")
Gene_Labels_EZH2<-**arrange**(Gene_Labels_EZH2, chromStart)
Gene_Labels_EZH2**$**chrom<-**factor**(Gene_Labels_EZH2**$**chrom,
 levels=**c**("chr1", "chr2", "chr3", "chr4",
 "chr5", "chr6", "chr7", "chr8",
 "chr9", "chr10", "chr11", "chr12",
 "chr13", "chr14", "chr15", "chr16",
 "chr17", "chr18", "chr19", "chr20",
 "chr21", "chr22", "chr23", "chrX",
 "chrY"))
Gene_Labels_EZH2<-Gene_Labels_EZH2[**order**(Gene_Labels_EZH2**$**chrom),]
Gene_Labels_EZH2<-Gene_Labels_EZH2[**!duplicated**(Gene_Labels_EZH2[,4]),] **circos.genomicLabels**(Gene_Labels_EZH2, labels.column=4, side='outside', cex=1)

###UP ONLY (to Color RED)
EZH2_DMR.Targets_UP<-**read.csv**("../1_Input/5_EZH2/EZH2_DMR.Labels_UP.csv")
EZH2_Anchor<-**read.csv**("../1_Input/5_EZH2/EZH2_Anchor.csv")
EZH2_DMR.Targets_UP**$**chrom<-**factor**(EZH2_DMR.Targets_UP**$**chrom,
 levels=**c**("chr1", "chr2", "chr3", "chr4",
 "chr5", "chr6", "chr7", "chr8",
 "chr9", "chr10", "chr11", "chr12",
 "chr13", "chr14", "chr15", "chr16",
 "chr17", "chr18", "chr19", "chr20",
 "chr21", "chr22", "chr23", "chrX",
 "chrY"))
EZH2_DMR.Targets_UP<-EZH2_DMR.Targets_UP[**order**(EZH2_DMR.Targets_UP**$**chrom),] **circos.genomicLink**(EZH2_DMR.Targets_UP, EZH2_Anchor[1**:nrow**(EZH2_DMR.Targets_UP),],
 col="goldenrod2", lwd=2)

###DOWN ONLY (to Color Green)
EZH2_DMR.Targets_DOWN<-**read.csv**("../1_Input/5_EZH2/EZH2_DMR.Labels_DOWN.csv")
EZH2_DMR.Targets_DOWN**$**chrom<-**factor**(EZH2_DMR.Targets_DOWN**$**chrom,
 levels=**c**("chr1", "chr2", "chr3", "chr4",
 "chr5", "chr6", "chr7", "chr8",
 "chr9", "chr10", "chr11", "chr12",
 "chr13", "chr14", "chr15", "chr16",
 "chr17", "chr18", "chr19", "chr20",
 "chr21", "chr22", "chr23", "chrX",
 "chrY"))
EZH2_DMR.Targets_DOWN<-EZH2_DMR.Targets_DOWN[**order**(EZH2_DMR.Targets_DOWN**$**chrom),] **circos.genomicLink**(EZH2_DMR.Targets_DOWN, EZH2_Anchor[1**:nrow**(EZH2_DMR.Targets_DOWN),],
 col="dodgerblue3", lwd=2)

Supplemental Information: R Session Information.

All packages and setting are acquired using the following command:

sinfo<-devtools**::session_info**()
sinfo**$**platform

## setting value
## version R version 3.4.2 (2017-09-28)
## system x86_64, darwin15.6.0
## ui X11
## language (EN)
## collate en_US.UTF-8
## tz <NA>
## date 2017-12-18

sinfo**$**packages **%>%** **kable**(
 align="c",
 longtable=T,
 booktabs=T,
 caption="Packages and Required Dependencies") **%>%**
 **kable_styling**(latex_options=**c**("striped", "repeat_header", "condensed"))

Table S1. Empirical DNA-Binding Protein Enrichment of Differentially-Expressed Gene Promoters (-1kB → +500B) with ENCODE ChIP-Sequencing Database.

| **Transcription**  **Factor** | **Overlap** | **-Log10(Q-Value)** | **-Log (P-value)** | **Z-score** | **Combined Score** |
| --- | --- | --- | --- | --- | --- |
| **EZH2** | 234/2000 | 10.15 | 13.07 | -1.63 | 49.08 |
| **EP300** | 227/2000 | 8.69 | 11.31 | -1.79 | 46.47 |
| **FOXM1** | 34/168 | 4.88 | 7.31 | -1.93 | 32.52 |
| **TCF12** | 101/857 | 3.60 | 5.91 | -1.89 | 25.68 |
| **RAD21** | 188/1973 | 2.01 | 4.11 | -1.66 | 15.71 |
| **SMC3** | 190/2000 | 2.01 | 4.07 | -1.69 | 15.81 |
| **RELA** | 86/785 | 1.97 | 3.96 | -1.75 | 15.92 |
| **SUZ12** | 189/2000 | 1.97 | 3.93 | -1.58 | 14.29 |
| **EZH2** | 188/2000 | 1.88 | 3.79 | -1.68 | 14.66 |
| **TCF12** | 123/1223 | 1.88 | 3.75 | -1.80 | 15.53 |
| **IKZF1** | 184/2000 | 1.41 | 3.24 | -1.68 | 12.51 |
| **SPI1** | 107/1074 | 1.41 | 3.19 | -1.72 | 12.64 |
| **SPI1** | 189/2068 | 1.41 | 3.18 | -1.71 | 12.52 |
| **MYOD1** | 102/1026 | 1.30 | 3.03 | -1.78 | 12.42 |
| **NFIC** | 70/663 | 1.19 | 2.90 | -1.83 | 12.19 |
| **GATA3** | 114/1185 | 1.14 | 2.82 | -1.78 | 11.53 |
| **CBX2** | 178/2000 | 0.88 | 2.51 | -1.71 | 9.92 |
| **POLR2A** | 178/2000 | 0.88 | 2.51 | -1.59 | 9.18 |
| **NR3C1** | 29/237 | 0.73 | 2.34 | -1.84 | 9.93 |
| **CBX8** | 175/2000 | 0.60 | 2.18 | -1.67 | 8.40 |
| **ZC3H11A** | 128/1429 | 0.46 | 2.03 | -1.71 | 7.98 |
| **CEBPB** | 63/640 | 0.45 | 2.00 | -1.82 | 8.39 |
| **POLR2A** | 172/2000 | 0.35 | 1.88 | -1.51 | 6.56 |
| **EZH2** | 169/2000 | 0.11 | 1.60 | -1.58 | 5.83 |
| **POLR2A** | 169/2000 | 0.11 | 1.60 | -1.55 | 5.74 |

Table S2. EZH2 Expression Post-LAD Ligation (Tarnaski *et al.* PMID: 14679301;GDS488)

| **Group** | **Log_2_(Fold-Change)** | **Std. Dev.** | **SEM** |
| --- | --- | --- | --- |
| **1 hr** | 0.38 | 0.36 | 0.25 |
| **4 hrs** | 0.73 | 0.17 | 0.12 |
| **24 hrs** | 0.69 | 0.05 | 0.04 |
| **48 hrs** | 1.52 | 0.32 | 0.22 |
| **1 wk** | 2.09 | 0.33 | 0.23 |
| **CON (1 hr sham)** | 0 | 0.08 | 0.06 |

Table S3. Methylation Distribution (P < 0.05)

|  | **North Shelf** | **North Shore** | **CpG Island** | **South Shore** | **South Shelf** | **Open Sea** |
| --- | --- | --- | --- | --- | --- | --- |
| **Promoter** | 139 | 3872 | 11946 | 3251 | 109 | 2241 |
| **TSS1500** | 82 | 2647 | 4994 | 2305 | 59 | 1093 |
| **TSS200** | 57 | 1225 | 6952 | 946 | 50 | 1148 |
| **5’ UTR** | 176 | 1138 | 5244 | 1024 | 125 | 1458 |
| **1st Exon** | 31 | 379 | 4284 | 342 | 19 | 618 |
| **Body** | 512 | 2154 | 4927 | 1741 | 405 | 4340 |
| **3’ UTR** | 56 | 163 | 228 | 115 | 37 | 438 |
| **Other** | 418 | 1174 | 3002 | 835 | 389 | 3903 |

Table S4. Differentially Methylated Promoter CpG Islands with Inversely Expressed Genes (P<0.05).

| **Table S4 Probe ID** | **Gene** | **RNA Sequencing** | | |  | **DNA Methylation** | |
| --- | --- | --- | --- | --- | --- | --- | --- |
|  |  | **F.C.** | **P-value** | **Q-value** |  | **% Change** | **P-value** |
| **cg00142925** | APOA1 | -2.11 | 5.0E-05 | 0.004 |  | 15.16 | 2.3E-03 |
| **cg01732804** | TUBA4A | -1.93 | 5.0E-05 | 0.004 |  | 42.25 | 7.3E-03 |
| **cg17731992** | HLA-B | -3.29 | 5.0E-05 | 0.004 |  | 30.72 | 1.0E-02 |
| **cg18013012** | TUBA4A | -1.93 | 5.0E-05 | 0.004 |  | 16.85 | 4.3E-02 |
| **cg18332146** | CTSC | 2.00 | 5.0E-05 | 0.004 |  | -7.46 | 4.4E-02 |
| **cg23369371** | TUBA4A | -1.93 | 5.0E-05 | 0.004 |  | 15.17 | 4.9E-02 |
| **cg23702897** | MXD4 | -1.97 | 5.0E-05 | 0.004 |  | 16.26 | 4.2E-02 |
| **cg24269434** | KLF15 | -2.01 | 5.0E-05 | 0.004 |  | 31.35 | 1.1E-03 |
| **cg27358585.2** | HLA-C | -7.79 | 5.0E-05 | 0.004 |  | 32.71 | 1.9E-04 |
| **cg02264038** | RRAS2 | -1.72 | 1.0E-04 | 0.006 |  | 16.71 | 3.2E-02 |
| **cg00862770** | FKBP5 | -2.65 | 1.5E-04 | 0.009 |  | 19.72 | 4.3E-02 |
| **cg27660920** | FAM83D | 3.37 | 2.0E-04 | 0.011 |  | -9.44 | 2.8E-02 |
| **cg08226146** | RCAN1 | -1.59 | 3.5E-04 | 0.017 |  | 16.14 | 5.5E-04 |
| **cg24033122** | ITGAL | 2.50 | 4.5E-04 | 0.021 |  | -14.11 | 3.7E-02 |
| **cg27358585** | HLA-C | -2.28 | 4.5E-04 | 0.021 |  | 32.71 | 1.9E-04 |
| **cg05756848** | AGPAT9 | -1.60 | 5.0E-04 | 0.022 |  | 40.97 | 4.0E-02 |
| **cg10685945** | AGPAT9 | -1.60 | 5.0E-04 | 0.022 |  | 25.53 | 4.2E-02 |
| **cg22744368** | SLCO4A1 | -2.47 | 8.5E-04 | 0.033 |  | 17.73 | 4.7E-02 |
| **cg18262201** | PFKFB3 | 1.73 | 9.0E-04 | 0.035 |  | -14.15 | 3.1E-02 |
| **cg08296680** | SLC16A3 | 1.83 | 1.2E-03 | 0.043 |  | -11.02 | 3.9E-02 |
| **cg14654468** | SLC16A3 | 1.83 | 1.2E-03 | 0.043 |  | -9.06 | 4.0E-02 |
| **cg21327887** | MAPT | -1.91 | 1.5E-03 | 0.049 |  | 50.49 | 2.6E-02 |
| **cg21477691** | ARID5A | -1.58 | 1.5E-03 | 0.050 |  | 27.13 | 4.5E-02 |
| **cg08160619** | CORO1A | 1.84 | 1.7E-03 | 0.054 |  | -22.09 | 3.1E-02 |
| **cg03615269** | TRPS1 | 1.67 | 1.8E-03 | 0.056 |  | -15.23 | 2.7E-02 |
| **cg06368590** | TRPS1 | 1.67 | 1.8E-03 | 0.056 |  | -24.63 | 1.7E-02 |
| **cg16821992** | TRPS1 | 1.67 | 1.8E-03 | 0.056 |  | -18.85 | 7.9E-03 |
| **cg14889643** | BCL2 | -1.55 | 1.8E-03 | 0.056 |  | 14.71 | 6.9E-03 |
| **cg21870469** | GMPR | -1.51 | 1.8E-03 | 0.056 |  | 20.29 | 2.5E-02 |
| **cg25839562** | GMPR | -1.51 | 1.8E-03 | 0.056 |  | 55.94 | 2.2E-03 |
| **cg06919956** | MRPL41 | -1.51 | 2.0E-03 | 0.060 |  | 28.87 | 6.8E-03 |
| **cg18791923** | MRPL33 | -1.57 | 2.1E-03 | 0.062 |  | 44.75 | 9.9E-03 |
| **cg22067325** | MRPL33 | -1.57 | 2.1E-03 | 0.062 |  | 20.30 | 2.3E-02 |
| **cg12429935** | C7orf60 | -1.57 | 2.1E-03 | 0.063 |  | 27.19 | 4.8E-02 |
| **cg05194250** | ID1 | -1.54 | 2.3E-03 | 0.068 |  | 18.11 | 2.8E-02 |
| **cg14353231** | ID1 | -1.54 | 2.3E-03 | 0.068 |  | 12.52 | 2.9E-02 |
| **cg26472301** | ID1 | -1.54 | 2.3E-03 | 0.068 |  | 36.65 | 7.8E-03 |
| **cg11470399** | PLK1 | 3.13 | 2.6E-03 | 0.074 |  | -6.21 | 4.5E-02 |
| **cg10973038** | GSTO1 | -1.50 | 2.8E-03 | 0.078 |  | 16.83 | 3.1E-02 |
| **cg01588438** | ADHFE1 | -1.52 | 3.5E-03 | 0.092 |  | 21.50 | 3.2E-02 |
| **cg20295442** | ADHFE1 | -1.52 | 3.5E-03 | 0.092 |  | 25.52 | 4.5E-02 |
| **cg06727855** | COX6C | -1.49 | 3.6E-03 | 0.093 |  | 29.25 | 1.8E-02 |
| **cg01385367** | SLC25A30 | -1.50 | 3.8E-03 | 0.097 |  | 20.68 | 2.4E-02 |
| **cg06102790** | SLC25A30 | -1.50 | 3.8E-03 | 0.097 |  | 97.50 | 3.7E-02 |
| **cg01815035** | XPR1 | -1.49 | 3.9E-03 | 0.099 |  | 30.62 | 1.9E-02 |
| **cg20141910** | XPR1 | -1.49 | 3.9E-03 | 0.099 |  | 22.31 | 3.6E-02 |
| **cg01290701** | XRCC4 | -1.81 | 4.0E-03 | 0.099 |  | 14.56 | 4.4E-02 |
| **cg04360080** | MTTP | -2.96 | 4.0E-03 | 0.099 |  | 47.71 | 3.2E-02 |
| **cg07472158** | MTTP | -2.96 | 4.0E-03 | 0.099 |  | 28.32 | 1.0E-02 |
| **cg11398097** | MTTP | -2.96 | 4.0E-03 | 0.099 |  | 24.01 | 3.6E-03 |
| **cg25971314** | XRCC4 | -1.81 | 4.0E-03 | 0.099 |  | 10.73 | 4.6E-02 |
| **cg02687417** | SDC4 | -1.45 | 4.1E-03 | 0.103 |  | 13.67 | 3.0E-02 |
| **cg24395907** | ALDH4A1 | -1.50 | 4.6E-03 | 0.110 |  | 12.79 | 4.6E-02 |
| **cg21746459** | ZNF189 | -1.58 | 4.9E-03 | 0.114 |  | 13.20 | 3.7E-02 |
| **cg10500218** | IER5 | -1.50 | 5.4E-03 | 0.123 |  | 22.86 | 2.0E-02 |
| **cg24503796** | SCD | 1.66 | 5.7E-03 | 0.129 |  | -11.35 | 3.6E-02 |
| **cg03724882** | CDKN3 | 2.16 | 6.4E-03 | 0.139 |  | -23.15 | 1.4E-02 |
| **cg02882301** | PPP1R3F | -1.50 | 6.8E-03 | 0.144 |  | 29.68 | 1.8E-02 |
| **cg12002260** | MYL12A | -2.03 | 6.8E-03 | 0.144 |  | 32.21 | 1.4E-02 |
| **cg12107637** | MYL12A | -2.03 | 6.8E-03 | 0.144 |  | 20.57 | 1.6E-02 |
| **cg14051366** | PPP1R3F | -1.50 | 6.8E-03 | 0.144 |  | 36.78 | 3.3E-03 |
| **cg21351102** | MYL12A | -2.03 | 6.8E-03 | 0.144 |  | 20.18 | 4.9E-02 |
| **cg13901901** | ACADM | -1.49 | 7.2E-03 | 0.150 |  | 42.83 | 2.0E-02 |
| **cg04835163** | FPGT | -1.42 | 8.5E-03 | 0.167 |  | 51.51 | 8.9E-03 |
| **cg04835163** | TNNI3K | -1.42 | 8.5E-03 | 0.167 |  | 51.51 | 8.9E-03 |
| **cg06464452** | STBD1 | -1.42 | 9.2E-03 | 0.178 |  | 24.08 | 4.2E-02 |
| **cg12973168** | PSMD8 | -1.45 | 9.2E-03 | 0.178 |  | 27.08 | 4.5E-02 |
| **cg20430816** | STBD1 | -1.42 | 9.2E-03 | 0.178 |  | 24.20 | 3.6E-02 |
| **cg15440363** | GPD1L | -1.48 | 9.4E-03 | 0.179 |  | 54.16 | 1.3E-03 |
| **cg04019128** | EIF1B | -1.44 | 9.6E-03 | 0.182 |  | 21.24 | 3.4E-02 |
| **cg15151911** | EIF1B | -1.44 | 9.6E-03 | 0.182 |  | 19.95 | 4.7E-02 |
| **cg26167161** | EIF1B | -1.44 | 9.6E-03 | 0.182 |  | 16.21 | 2.2E-03 |
| **cg00896540** | FABP5 | -1.41 | 1.0E-02 | 0.190 |  | 19.53 | 1.9E-02 |
| **cg25995724** | FABP5 | -1.41 | 1.0E-02 | 0.190 |  | 10.38 | 2.9E-02 |
| **cg14345069** | UFSP2 | -1.44 | 1.0E-02 | 0.191 |  | 34.54 | 1.4E-02 |
| **cg01105385** | PIK3R1 | -1.51 | 1.1E-02 | 0.199 |  | 23.71 | 2.4E-02 |
| **cg20474370** | PIK3R1 | -1.51 | 1.1E-02 | 0.199 |  | 24.60 | 1.7E-02 |
| **cg15833596** | PFKFB2 | -1.51 | 1.1E-02 | 0.200 |  | 35.24 | 2.5E-03 |
| **cg20291513** | PFKFB2 | -1.51 | 1.1E-02 | 0.200 |  | 42.10 | 2.3E-02 |
| **cg22500102** | PFKFB2 | -1.51 | 1.1E-02 | 0.200 |  | 65.45 | 4.3E-03 |
| **cg14291622** | PLIN2 | -1.47 | 1.1E-02 | 0.204 |  | 50.14 | 1.0E-02 |
| **cg05029645** | MICA | -12.43 | 1.2E-02 | 0.212 |  | 10.53 | 3.6E-02 |
| **cg13829635** | MICA | -12.43 | 1.2E-02 | 0.212 |  | 17.12 | 4.3E-02 |
| **cg17221483** | MICA | -12.43 | 1.2E-02 | 0.212 |  | 15.80 | 1.2E-02 |
| **cg21915666** | MICA | -12.43 | 1.2E-02 | 0.212 |  | 26.52 | 1.5E-02 |
| **cg00824793** | WDR31 | -1.49 | 1.3E-02 | 0.215 |  | 29.18 | 3.6E-02 |
| **cg04096435** | AMD1 | -1.41 | 1.3E-02 | 0.215 |  | 22.67 | 3.2E-02 |
| **cg15077919** | AMD1 | -1.41 | 1.3E-02 | 0.215 |  | 46.99 | 4.6E-02 |
| **cg15211596** | AMD1 | -1.41 | 1.3E-02 | 0.215 |  | 22.63 | 2.0E-02 |
| **cg00132616** | ARID5B | -1.41 | 1.3E-02 | 0.218 |  | 14.72 | 4.8E-02 |
| **cg03020000** | ARID5B | -1.41 | 1.3E-02 | 0.218 |  | 15.43 | 4.8E-02 |
| **cg03361817** | ARID5B | -1.41 | 1.3E-02 | 0.218 |  | 37.13 | 1.8E-03 |
| **cg03077572** | PCYT2 | -1.43 | 1.3E-02 | 0.219 |  | 31.13 | 4.3E-02 |
| **cg19863411** | PCYT2 | -1.43 | 1.3E-02 | 0.219 |  | 14.99 | 4.8E-02 |
| **cg20248866** | PCYT2 | -1.43 | 1.3E-02 | 0.219 |  | 10.07 | 2.8E-03 |
| **cg26986937** | ETV1 | -1.41 | 1.4E-02 | 0.229 |  | 34.72 | 4.3E-03 |
| **cg08732750** | LOC339524 | 1.55 | 1.4E-02 | 0.231 |  | -7.93 | 4.7E-02 |
| **cg11582018** | LOC339524 | 1.55 | 1.4E-02 | 0.231 |  | -18.22 | 1.5E-02 |
| **cg26853048** | HSPA4 | -1.41 | 1.4E-02 | 0.233 |  | 12.70 | 3.2E-02 |
| **cg10439144** | CPEB4 | -1.42 | 1.6E-02 | 0.255 |  | 33.64 | 6.8E-05 |
| **cg03442014** | NDUFAB1 | -1.36 | 1.6E-02 | 0.256 |  | 16.26 | 2.1E-02 |
| **cg27358585** | HLA-C | -1.94 | 1.7E-02 | 0.259 |  | 32.71 | 1.9E-04 |
| **cg11945251** | RPLP1 | -1.40 | 1.7E-02 | 0.267 |  | 30.70 | 9.5E-03 |
| **cg14833254** | RPLP1 | -1.40 | 1.7E-02 | 0.267 |  | 17.83 | 4.4E-02 |
| **cg14591386** | NDUFAF4 | -1.38 | 1.8E-02 | 0.278 |  | 11.20 | 1.6E-02 |
| **cg24456602** | TMEM126A | -1.51 | 1.9E-02 | 0.278 |  | 22.12 | 2.2E-02 |
| **cg26778336** | SRCIN1 | -1.73 | 1.9E-02 | 0.280 |  | 11.24 | 4.4E-02 |
| **cg12646585** | WDYHV1 | -1.54 | 1.9E-02 | 0.284 |  | 23.13 | 2.7E-02 |
| **cg16249105** | PRDX3 | -1.36 | 2.0E-02 | 0.287 |  | 11.33 | 3.7E-02 |
| **cg22553140** | PRDX3 | -1.36 | 2.0E-02 | 0.287 |  | 62.79 | 4.4E-03 |
| **cg02186441** | MDH1 | -1.45 | 2.0E-02 | 0.290 |  | 31.61 | 3.1E-02 |
| **cg14736172** | MDH1 | -1.45 | 2.0E-02 | 0.290 |  | 14.31 | 2.6E-02 |
| **cg17972846** | MDH1 | -1.45 | 2.0E-02 | 0.290 |  | 36.41 | 1.7E-02 |
| **cg22513888** | MDH1 | -1.45 | 2.0E-02 | 0.290 |  | 46.83 | 5.4E-03 |
| **cg22298206** | SMAD6 | -1.45 | 2.0E-02 | 0.291 |  | 38.69 | 2.1E-02 |
| **cg04544154** | ELF4 | 1.53 | 2.0E-02 | 0.293 |  | -5.29 | 9.6E-03 |
| **cg02738763** | TARSL2 | -1.37 | 2.1E-02 | 0.303 |  | 45.14 | 4.5E-02 |
| **cg05201033** | TARSL2 | -1.37 | 2.1E-02 | 0.303 |  | 27.14 | 3.1E-02 |
| **cg15670579** | TARSL2 | -1.37 | 2.1E-02 | 0.303 |  | 18.31 | 4.0E-02 |
| **cg09655497** | TMEM164 | -1.36 | 2.2E-02 | 0.307 |  | 29.18 | 2.5E-02 |
| **cg20589243** | TMEM164 | -1.36 | 2.2E-02 | 0.307 |  | 23.21 | 2.0E-02 |
| **cg16228589** | NCKIPSD | -1.41 | 2.2E-02 | 0.307 |  | 16.44 | 2.9E-02 |
| **cg22329233** | NCKIPSD | -1.41 | 2.2E-02 | 0.307 |  | 18.84 | 2.8E-02 |
| **cg06902669** | DECR1 | -1.38 | 2.2E-02 | 0.307 |  | 18.85 | 2.1E-02 |
| **cg02994018** | NDUFB3 | -1.36 | 2.3E-02 | 0.316 |  | 28.60 | 2.7E-02 |
| **cg08409687** | NDUFB3 | -1.36 | 2.3E-02 | 0.316 |  | 11.53 | 5.0E-02 |
| **cg17079352** | NDUFB3 | -1.36 | 2.3E-02 | 0.316 |  | 25.90 | 2.9E-02 |
| **cg07073120** | DCXR | -1.38 | 2.3E-02 | 0.318 |  | 15.69 | 2.5E-02 |
| **cg16687867** | DLAT | -1.38 | 2.4E-02 | 0.321 |  | 21.37 | 3.3E-02 |
| **cg00636809** | EGR2 | 1.66 | 2.4E-02 | 0.322 |  | -6.55 | 3.3E-02 |
| **cg02165720** | NDUFB11 | -1.35 | 2.4E-02 | 0.322 |  | 17.30 | 3.6E-02 |
| **cg05418506** | NDUFB11 | -1.35 | 2.4E-02 | 0.322 |  | 26.86 | 8.7E-03 |
| **cg11663421** | NDUFB11 | -1.35 | 2.4E-02 | 0.322 |  | 25.20 | 9.6E-03 |
| **cg18140045** | NDUFB11 | -1.35 | 2.4E-02 | 0.322 |  | 31.40 | 1.9E-02 |
| **cg18388748** | NDUFB11 | -1.35 | 2.4E-02 | 0.322 |  | 37.15 | 6.1E-03 |
| **cg27477594** | MGST3 | -1.37 | 2.5E-02 | 0.332 |  | 22.40 | 1.2E-02 |
| **cg21733597** | IDH3A | -1.37 | 2.6E-02 | 0.338 |  | 56.94 | 4.3E-03 |
| **cg06291846** | CEBPB | -1.39 | 2.6E-02 | 0.340 |  | 27.89 | 4.8E-03 |
| **cg05388468** | GLRX2 | -1.54 | 2.6E-02 | 0.343 |  | 21.72 | 3.6E-02 |
| **cg05822179** | GLRX2 | -1.54 | 2.6E-02 | 0.343 |  | 13.61 | 8.6E-03 |
| **cg08398452** | GLRX2 | -1.54 | 2.6E-02 | 0.343 |  | 24.75 | 3.7E-02 |
| **cg18267434** | GLRX2 | -1.54 | 2.6E-02 | 0.343 |  | 35.28 | 2.8E-04 |
| **cg26924209** | GLRX2 | -1.54 | 2.6E-02 | 0.343 |  | 16.86 | 5.5E-03 |
| **cg14407075** | LARP4 | -1.35 | 2.7E-02 | 0.348 |  | 11.93 | 3.6E-02 |
| **cg18712503** | LARP4 | -1.35 | 2.7E-02 | 0.348 |  | 47.51 | 2.3E-02 |
| **cg11283640** | NDUFB4 | -1.35 | 2.8E-02 | 0.352 |  | 16.97 | 2.1E-02 |
| **cg11283995** | NDUFB4 | -1.35 | 2.8E-02 | 0.352 |  | 15.00 | 4.2E-02 |
| **cg23647884** | NDUFB4 | -1.35 | 2.8E-02 | 0.352 |  | 29.07 | 1.9E-02 |
| **cg20474699** | ANKRD9 | -1.41 | 2.8E-02 | 0.358 |  | 38.64 | 4.2E-02 |
| **cg23080985** | ALKBH7 | -1.35 | 3.0E-02 | 0.370 |  | 35.18 | 4.3E-02 |
| **cg13483597** | TGFB1I1 | 1.37 | 3.0E-02 | 0.372 |  | -7.68 | 3.6E-02 |
| **cg00533811** | CHKB | -1.41 | 3.0E-02 | 0.373 |  | 33.45 | 1.7E-02 |
| **cg04798154** | CHKB | -1.41 | 3.0E-02 | 0.373 |  | 15.58 | 1.5E-02 |
| **cg10968404** | CHKB | -1.41 | 3.0E-02 | 0.373 |  | 27.08 | 2.5E-03 |
| **cg01388957** | CDC37L1 | -1.36 | 3.2E-02 | 0.382 |  | 19.73 | 1.5E-02 |
| **cg01558660** | CDC37L1 | -1.36 | 3.2E-02 | 0.382 |  | 86.65 | 1.7E-04 |
| **cg14075496** | CDC37L1 | -1.36 | 3.2E-02 | 0.382 |  | 21.36 | 4.3E-02 |
| **cg05029645** | MICA | -2.21 | 3.2E-02 | 0.383 |  | 10.53 | 3.6E-02 |
| **cg13829635** | MICA | -2.21 | 3.2E-02 | 0.383 |  | 17.12 | 4.3E-02 |
| **cg17221483** | MICA | -2.21 | 3.2E-02 | 0.383 |  | 15.80 | 1.2E-02 |
| **cg21915666** | MICA | -2.21 | 3.2E-02 | 0.383 |  | 26.52 | 1.5E-02 |
| **cg05406791** | CSDA | -1.43 | 3.2E-02 | 0.384 |  | 30.35 | 2.9E-02 |
| **cg15125566** | RASA3 | 1.37 | 3.3E-02 | 0.387 |  | -8.25 | 3.3E-02 |
| **cg05529097** | FXR2 | -1.36 | 3.3E-02 | 0.394 |  | 53.00 | 3.1E-03 |
| **cg05913233** | FXR2 | -1.36 | 3.3E-02 | 0.394 |  | 18.39 | 3.4E-02 |
| **cg18754754** | NDUFB5 | -1.36 | 3.4E-02 | 0.396 |  | 22.15 | 1.9E-02 |
| **cg23168649** | NDUFB5 | -1.36 | 3.4E-02 | 0.396 |  | 29.42 | 1.7E-02 |
| **cg14836868** | POLB | -1.49 | 3.4E-02 | 0.396 |  | 13.54 | 4.1E-02 |
| **cg18894794** | POLB | -1.49 | 3.4E-02 | 0.396 |  | 23.43 | 2.7E-02 |
| **cg23279929** | POLB | -1.49 | 3.4E-02 | 0.396 |  | 32.17 | 1.9E-02 |
| **cg07012573** | BEX4 | -1.32 | 3.4E-02 | 0.396 |  | 33.61 | 1.9E-02 |
| **cg15362194** | COX5B | -1.36 | 3.4E-02 | 0.399 |  | 42.41 | 2.3E-02 |
| **cg26880806** | COX5B | -1.36 | 3.4E-02 | 0.399 |  | 23.77 | 2.8E-03 |
| **cg10055139** | C11orf52 | -1.33 | 3.6E-02 | 0.411 |  | 25.27 | 2.3E-02 |
| **cg24676534** | UQCRQ | -1.32 | 3.7E-02 | 0.417 |  | 12.01 | 7.9E-03 |
| **cg00672228** | ACADVL | -1.43 | 3.7E-02 | 0.418 |  | 14.67 | 1.2E-02 |
| **cg12709244** | ACADVL | -1.43 | 3.7E-02 | 0.418 |  | 52.42 | 7.2E-03 |
| **cg16862315** | ACADVL | -1.43 | 3.7E-02 | 0.418 |  | 22.28 | 1.2E-02 |
| **cg16399182** | COQ3 | -1.43 | 3.8E-02 | 0.421 |  | 41.37 | 1.7E-02 |
| **cg08595182** | MRPL46 | -1.34 | 3.8E-02 | 0.422 |  | 38.51 | 5.0E-02 |
| **cg13746225** | MRPL46 | -1.34 | 3.8E-02 | 0.422 |  | 22.53 | 3.0E-02 |
| **cg27326431** | MRPL46 | -1.34 | 3.8E-02 | 0.422 |  | 19.88 | 1.3E-02 |
| **cg15473751** | CKB | -1.37 | 3.9E-02 | 0.425 |  | 31.84 | 2.5E-02 |
| **cg12785122** | SCO1 | -1.34 | 3.9E-02 | 0.428 |  | 11.98 | 4.6E-02 |
| **cg09523275** | NKAPL | -1.59 | 4.0E-02 | 0.434 |  | 20.21 | 1.1E-02 |
| **cg17384889** | NKAPL | -1.59 | 4.0E-02 | 0.434 |  | 48.75 | 1.5E-05 |
| **cg10252135** | PDE7A | -1.34 | 4.1E-02 | 0.437 |  | 12.30 | 3.8E-02 |
| **cg02751449** | TRPC1 | -1.37 | 4.2E-02 | 0.445 |  | 29.43 | 2.7E-02 |
| **cg03162045** | TRPC1 | -1.37 | 4.2E-02 | 0.445 |  | 10.84 | 1.5E-02 |
| **cg20259256** | TRPC1 | -1.37 | 4.2E-02 | 0.445 |  | 15.75 | 3.9E-02 |
| **cg24794531** | TRPC1 | -1.37 | 4.2E-02 | 0.445 |  | 23.68 | 3.3E-02 |
| **cg05474467** | BTBD9 | -1.33 | 4.3E-02 | 0.449 |  | 19.09 | 2.1E-03 |
| **cg12383181** | BTBD9 | -1.33 | 4.3E-02 | 0.449 |  | 34.38 | 4.5E-02 |
| **cg24659411** | BTBD9 | -1.33 | 4.3E-02 | 0.449 |  | 12.10 | 1.8E-02 |
| **cg08705697** | PCDH9 | -1.35 | 4.3E-02 | 0.449 |  | 20.83 | 5.5E-03 |
| **cg01051318** | CAD | 1.39 | 4.3E-02 | 0.449 |  | -16.62 | 3.6E-02 |
| **cg13207467** | CSRP1 | 1.31 | 4.4E-02 | 0.451 |  | -22.38 | 1.4E-02 |
| **cg13554744** | CSRP1 | 1.31 | 4.4E-02 | 0.451 |  | -45.36 | 5.0E-04 |
| **cg04053638** | SUCLG1 | -1.36 | 4.4E-02 | 0.451 |  | 38.88 | 1.6E-02 |
| **cg06700871** | SUCLG1 | -1.36 | 4.4E-02 | 0.451 |  | 24.76 | 6.0E-03 |
| **cg02666184** | ETFB | -1.30 | 4.4E-02 | 0.454 |  | 28.20 | 2.6E-02 |
| **cg07757224** | ETFB | -1.30 | 4.4E-02 | 0.454 |  | 34.49 | 1.5E-03 |
| **cg24129914** | ETFB | -1.30 | 4.4E-02 | 0.454 |  | 44.70 | 2.1E-02 |
| **cg04102208** | ZNF622 | -1.31 | 4.5E-02 | 0.457 |  | 10.58 | 1.6E-03 |
| **cg27223343** | MAP3K6 | -1.32 | 4.5E-02 | 0.458 |  | 19.69 | 4.2E-02 |
| **cg03024517** | EHBP1L1 | -1.32 | 4.6E-02 | 0.460 |  | 31.66 | 4.1E-02 |
| **cg26060296** | NDUFS6 | -1.30 | 4.6E-02 | 0.460 |  | 15.16 | 4.3E-02 |
| **cg03861428** | SDHB | -1.31 | 4.7E-02 | 0.467 |  | 28.85 | 2.0E-02 |
| **cg00926162** | COX5A | -1.31 | 4.8E-02 | 0.477 |  | 32.70 | 2.6E-02 |
| **cg10292851** | ANKRD46 | -1.35 | 4.9E-02 | 0.480 |  | 9.67 | 1.6E-02 |
| **cg23123694** | SLC38A2 | -1.31 | 4.9E-02 | 0.480 |  | 26.26 | 1.8E-02 |

Table S5. Gene Set Enrichment of Transcriptionally-Active Promoter DMC's via WebGestalt using the Reactome Pathway Database.

| **Description** | **# Genes** | **FDR** |
| --- | --- | --- |
| **The citric acid (TCA) cycle and respiratory electron transport** | 19 | 0 |
| **Respiratory electron transport** | 14 | 2.4E-11 |
| **Complex I biogenesis** | 7 | 9.4E-05 |
| **Pyruvate metabolism and Citric Acid (TCA) cycle** | 6 | 5.8E-04 |
| **Mitochondrial Fatty Acid Beta-Oxidation** | 4 | 5.7E-03 |

Table S6. Consensus Sequence Enrichment of Differential Expressed Gene (P < 0.05) Proximal Promoters (-1kB to +500kB), using JASPAR database and Enrichr algorithm.

| **Table S6**  **Transcription**  **Factor** | **Overlap** | **P-value** | **Adj. P-value** | **Z-score** | **Combined Score** |
| --- | --- | --- | --- | --- | --- |
| **KLF11 (human)** | 163/1388 | 6.6E-10 | 2.2E-07 | -1.8 | 37.0 |
| **KLF4 (human)** | 170/1485 | 1.9E-09 | 3.1E-07 | -1.7 | 34.4 |
| **ZNF148 (human)** | 175/1591 | 2.0E-08 | 2.2E-06 | -1.7 | 30.3 |
| **MIR138 (human)** | 150/1356 | 1.6E-07 | 1.3E-05 | -1.7 | 27.0 |
| **ELF3 (human)** | 155/1488 | 3.7E-06 | 2.4E-04 | -1.7 | 21.0 |
| **CBEPA (human)** | 141/1366 | 1.7E-05 | 9.1E-04 | -1.7 | 18.4 |
| **CACYBP (human)** | 139/1350 | 2.2E-05 | 9.1E-04 | -1.7 | 18.0 |
| **MZF1 (human)** | 142/1389 | 2.5E-05 | 9.1E-04 | -1.6 | 17.3 |
| **SREBF1 (human)** | 242/2587 | 2.3E-05 | 9.1E-04 | -1.6 | 16.8 |
| **TFAP2A (mouse)** | 164/1663 | 4.1E-05 | 1.3E-03 | -1.6 | 15.9 |
| **EGR1 (human)** | 77/681 | 9.1E-05 | 2.1E-03 | -1.7 | 15.7 |
| **ZBTB7A (human)** | 138/1373 | 7.3E-05 | 2.1E-03 | -1.6 | 15.2 |
| **ETV4 (human)** | 140/1403 | 9.1E-05 | 2.1E-03 | -1.6 | 14.9 |
| **TEAD2 (human)** | 147/1486 | 9.1E-05 | 2.1E-03 | -1.6 | 14.7 |
| **TFAP2A (human)** | 317/3613 | 1.7E-04 | 3.7E-03 | -1.6 | 13.9 |
| **RELA (human)** | 250/2775 | 2.0E-04 | 4.1E-03 | -1.5 | 12.4 |
| **HINFP (human)** | 269/3047 | 4.1E-04 | 7.8E-03 | -1.4 | 10.9 |
| **ZNF281 (human)** | 100/986 | 5.3E-04 | 9.7E-03 | -1.6 | 12.2 |
| **JUN (human)** | 262/2976 | 5.8E-04 | 1.0E-02 | -1.4 | 10.4 |
| **NFE2 (human)** | 237/2681 | 8.9E-04 | 1.4E-02 | -1.4 | 9.8 |
| **TCFAP2A (human)** | 130/1367 | 1.1E-03 | 1.6E-02 | -1.5 | 10.1 |
| **TFAP2C (human)** | 140/1485 | 1.0E-03 | 1.6E-02 | -1.4 | 9.9 |
| **TEAD4 (human)** | 129/1354 | 1.1E-03 | 1.6E-02 | -1.5 | 9.9 |
| **SPI1 (human)** | 152/1638 | 1.2E-03 | 1.6E-02 | -1.5 | 10.3 |
| **SMAD4 (human)** | 144/1542 | 1.3E-03 | 1.6E-02 | -1.5 | 9.7 |
| **CEBPB (human)** | 134/1420 | 1.3E-03 | 1.6E-02 | -1.4 | 9.6 |
| **REPIN1 (human)** | 137/1466 | 1.6E-03 | 2.0E-02 | -1.4 | 9.2 |
| **NR5A2 (human)** | 322/3815 | 2.1E-03 | 2.4E-02 | -1.2 | 7.5 |
| **SP3 (human)** | 125/1332 | 2.2E-03 | 2.5E-02 | -1.4 | 8.6 |
| **NFKB1 (human)** | 297/3497 | 2.3E-03 | 2.5E-02 | -1.2 | 7.4 |
| **RELB (human)** | 136/1469 | 2.4E-03 | 2.5E-02 | -1.4 | 8.1 |
| **KLF13 (human)** | 134/1453 | 3.0E-03 | 3.0E-02 | -1.3 | 7.8 |
| **SP3 (mouse)** | 107/1129 | 3.4E-03 | 3.3E-02 | -1.3 | 7.7 |
| **NKX2-8 (human)** | 31/256 | 4.0E-03 | 3.8E-02 | -1.5 | 8.0 |
| **PURA (human)** | 52/492 | 4.9E-03 | 4.5E-02 | -1.4 | 7.7 |
| **HNF4A (mouse)** | 49/461 | 5.6E-03 | 4.5E-02 | -1.4 | 7.1 |
| **USF1 (human)** | 131/1441 | 5.4E-03 | 4.5E-02 | -1.3 | 6.8 |
| **IRF8 (human)** | 140/1550 | 5.1E-03 | 4.5E-02 | -1.3 | 6.7 |
| **SP1 (human)** | 128/1406 | 5.6E-03 | 4.5E-02 | -1.3 | 6.5 |
| **MIB2 (human)** | 135/1490 | 5.3E-03 | 4.5E-02 | -1.2 | 6.4 |
| **NFYB (human)** | 30-Jul | 5.1E-03 | 4.5E-02 | -0.7 | 3.9 |
| **CACYBP (mouse)** | 148/1656 | 5.9E-03 | 4.6E-02 | -1.1 | 5.7 |
| **LEF1 (human)** | 381/4658 | 6.2E-03 | 4.7E-02 | -0.9 | 4.8 |

Table S7: R Bioinformatics Packages and Required Dependencies

| **Package** | **Version** | **Date** | **Source** |
| --- | --- | --- | --- |
| **acepack** | 1.4.1 | 10/29/16 | CRAN (R 3.4.0) |
| **annotate** | 1.56.1 | 11/13/17 | Bioconductor |
| **AnnotationDbi** | 1.40.0 | 10/31/17 | Bioconductor |
| **assertthat** | 0.2.0 | 4/11/17 | CRAN (R 3.4.0) |
| **backports** | 1.1.1 | 9/25/17 | CRAN (R 3.4.2) |
| **base** | 3.4.2 | 10/4/17 | local |
| **base64** | 2 | 5/10/16 | CRAN (R 3.4.0) |
| **base64enc** | 0.1-3 | 7/28/15 | CRAN (R 3.4.0) |
| **beanplot** | 1.2 | 9/19/14 | CRAN (R 3.4.0) |
| **bindr** | 0.1 | 11/13/16 | CRAN (R 3.4.0) |
| **bindrcpp** | 0.2 | 6/17/17 | CRAN (R 3.4.0) |
| **Biobase** | 2.38.0 | 10/31/17 | Bioconductor |
| **BiocGenerics** | 0.24.0 | 10/31/17 | Bioconductor |
| **BiocParallel** | 1.12.0 | 10/31/17 | Bioconductor |
| **biomaRt** | 2.34.0 | 10/31/17 | Bioconductor |
| **Biostrings** | 2.46.0 | 10/31/17 | Bioconductor |
| **bit** | 1.1-12 | 4/9/14 | CRAN (R 3.4.0) |
| **bit64** | 0.9-7 | 5/8/17 | CRAN (R 3.4.0) |
| **bitops** | 1.0-6 | 8/17/13 | CRAN (R 3.4.0) |
| **blob** | 1.1.0 | 6/17/17 | CRAN (R 3.4.0) |
| **bumphunter** | 1.20.0 | 10/31/17 | Bioconductor |
| **cellranger** | 1.1.0 | 7/27/16 | CRAN (R 3.4.0) |
| **checkmate** | 1.8.5 | 10/24/17 | CRAN (R 3.4.2) |
| **circlize** | 0.4.2 | 11/18/17 | CRAN (R 3.4.2) |
| **cluster** | 2.0.6 | 3/10/17 | CRAN (R 3.4.2) |
| **codetools** | 0.2-15 | 10/5/16 | CRAN (R 3.4.2) |
| **colorspace** | 1.3-2 | 12/14/16 | CRAN (R 3.4.0) |
| **compiler** | 3.4.2 | 10/4/17 | local |
| **corrplot** | 0.84 | 10/16/17 | CRAN (R 3.4.2) |
| **cowplot** | 0.9.1 | 11/16/17 | CRAN (R 3.4.2) |
| **crosstalk** | 1.0.0 | 12/21/16 | CRAN (R 3.4.0) |
| **data.table** | 1.10.4-3 | 10/27/17 | CRAN (R 3.4.2) |
| **datasets** | 3.4.2 | 10/4/17 | local |
| **DBI** | 0.7 | 6/18/17 | CRAN (R 3.4.0) |
| **DelayedArray** | 0.4.1 | 11/7/17 | Bioconductor |
| **devtools** | 1.13.4 | 11/9/17 | CRAN (R 3.4.2) |
| **digest** | 0.6.12 | 1/27/17 | CRAN (R 3.4.0) |
| **doRNG** | 1.6.6 | 4/10/17 | CRAN (R 3.4.0) |
| **dplyr** | 0.7.4 | 9/28/17 | CRAN (R 3.4.2) |
| **evaluate** | 0.10.1 | 6/24/17 | CRAN (R 3.4.1) |
| **foreach** | 1.4.3 | 10/13/15 | CRAN (R 3.4.0) |
| **foreign** | 0.8-69 | 6/22/17 | CRAN (R 3.4.2) |
| **Formula** | 1.2-2 | 7/10/17 | CRAN (R 3.4.1) |
| **genefilter** | 1.60.0 | 10/31/17 | Bioconductor |
| **GenomeInfoDb** | 1.14.0 | 10/31/17 | Bioconductor |
| **GenomeInfoDbData** | 0.99.1 | 11/25/17 | Bioconductor |
| **GenomicAlignments** | 1.14.1 | 11/18/17 | Bioconductor |
| **GenomicFeatures** | 1.30.0 | 10/31/17 | Bioconductor |
| **GenomicRanges** | 1.30.0 | 10/31/17 | Bioconductor |
| **GEOquery** | 2.46.9 | 11/22/17 | Bioconductor |
| **ggplot2** | 2.2.1 | 12/30/16 | CRAN (R 3.4.0) |
| **ggpubr** | 0.1.6 | 11/14/17 | CRAN (R 3.4.2) |
| **GlobalOptions** | 0.0.12 | 5/21/17 | CRAN (R 3.4.0) |
| **glue** | 1.2.0 | 10/29/17 | CRAN (R 3.4.2) |
| **graphics** | 3.4.2 | 10/4/17 | local |
| **grDevices** | 3.4.2 | 10/4/17 | local |
| **grid** | 3.4.2 | 10/4/17 | local |
| **gridExtra** | 2.3 | 9/9/17 | CRAN (R 3.4.1) |
| **gtable** | 0.2.0 | 2/26/16 | CRAN (R 3.4.0) |
| **gtools** | 3.5.0 | 5/29/15 | CRAN (R 3.4.0) |
| **highr** | 0.6 | 5/9/16 | CRAN (R 3.4.0) |
| **Hmisc** | 4.0-3 | 5/2/17 | CRAN (R 3.4.0) |
| **hms** | 0.4.0 | 11/23/17 | CRAN (R 3.4.3) |
| **htmlTable** | 1.9 | 1/26/17 | CRAN (R 3.4.0) |
| **htmltools** | 0.3.6 | 4/28/17 | CRAN (R 3.4.0) |
| **htmlwidgets** | 0.9 | 7/10/17 | CRAN (R 3.4.1) |
| **httpuv** | 1.3.5 | 7/4/17 | CRAN (R 3.4.1) |
| **httr** | 1.3.1 | 8/20/17 | CRAN (R 3.4.1) |
| **illuminaio** | 0.20.0 | 10/31/17 | Bioconductor |
| **IRanges** | 2.12.0 | 10/31/17 | Bioconductor |
| **iterators** | 1.0.8 | 10/13/15 | CRAN (R 3.4.0) |
| **jsonlite** | 1.5 | 6/1/17 | CRAN (R 3.4.0) |
| **kableExtra** | 0.6.1 | 11/1/17 | CRAN (R 3.4.2) |
| **knitr** | 1.17 | 8/10/17 | CRAN (R 3.4.1) |
| **labeling** | 0.3 | 8/23/14 | CRAN (R 3.4.0) |
| **lattice** | 0.20-35 | 3/25/17 | CRAN (R 3.4.2) |
| **latticeExtra** | 0.6-28 | 2/9/16 | CRAN (R 3.4.0) |
| **lazyeval** | 0.2.1 | 10/29/17 | CRAN (R 3.4.2) |
| **limma** | 3.34.2 | 11/27/17 | Bioconductor |
| **locfit** | 1.5-9.1 | 4/20/13 | CRAN (R 3.4.0) |
| **magrittr** | 1.5 | 11/22/14 | CRAN (R 3.4.0) |
| **MASS** | 7.3-47 | 2/26/17 | CRAN (R 3.4.2) |
| **Matrix** | 1.2-12 | 11/15/17 | CRAN (R 3.4.2) |
| **matrixStats** | 0.52.2 | 4/14/17 | CRAN (R 3.4.0) |
| **mclust** | 5.4 | 11/22/17 | CRAN (R 3.4.3) |
| **memoise** | 1.1.0 | 4/21/17 | CRAN (R 3.4.0) |
| **methods** | 3.4.2 | 10/4/17 | local |
| **mime** | 0.5 | 7/7/16 | CRAN (R 3.4.0) |
| **minfi** | 1.24.0 | 10/31/17 | Bioconductor |
| **multtest** | 2.34.0 | 10/31/17 | Bioconductor |
| **munsell** | 0.4.3 | 2/13/16 | CRAN (R 3.4.0) |
| **nlme** | 3.1-131 | 2/6/17 | CRAN (R 3.4.2) |
| **nnet** | 7.3-12 | 2/2/16 | CRAN (R 3.4.2) |
| **nor1mix** | 1.2-3 | 8/30/17 | CRAN (R 3.4.1) |
| **openssl** | 0.9.9 | 11/10/17 | CRAN (R 3.4.2) |
| **parallel** | 3.4.2 | 10/4/17 | local |
| **pheatmap** | 1.0.8 | 12/11/15 | CRAN (R 3.4.0) |
| **pkgconfig** | 2.0.1 | 3/21/17 | CRAN (R 3.4.0) |
| **pkgmaker** | 0.22 | 5/14/14 | CRAN (R 3.4.0) |
| **plotly** | 4.7.1 | 7/29/17 | CRAN (R 3.4.1) |
| **plyr** | 1.8.4 | 6/8/16 | CRAN (R 3.4.0) |
| **preprocessCore** | 1.40.0 | 10/31/17 | Bioconductor |
| **prettyunits** | 1.0.2 | 7/13/15 | CRAN (R 3.4.0) |
| **progress** | 1.1.2 | 12/14/16 | CRAN (R 3.4.0) |
| **purrr** | 0.2.4 | 10/18/17 | CRAN (R 3.4.2) |
| **quadprog** | 1.5-5 | 4/17/13 | CRAN (R 3.4.0) |
| **R6** | 2.2.2 | 6/17/17 | CRAN (R 3.4.0) |
| **RColorBrewer** | 1.1-2 | 12/7/14 | CRAN (R 3.4.0) |
| **Rcpp** | 0.12.14 | 11/23/17 | CRAN (R 3.4.2) |
| **RCurl** | 1.95-4.8 | 3/1/16 | CRAN (R 3.4.0) |
| **readr** | 1.1.1 | 5/16/17 | CRAN (R 3.4.0) |
| **readxl** | 1.0.0 | 4/18/17 | CRAN (R 3.4.0) |
| **registry** | 0.3 | 7/8/15 | CRAN (R 3.4.0) |
| **reshape** | 0.8.7 | 8/6/17 | CRAN (R 3.4.1) |
| **reshape2** | 1.4.2 | 10/22/16 | CRAN (R 3.4.0) |
| **rlang** | 0.1.4 | 11/5/17 | CRAN (R 3.4.2) |
| **rmarkdown** | 1.8.3 | 11/28/17 | Github |
| **RMySQL** | 0.10.13 | 8/14/17 | CRAN (R 3.4.1) |
| **rngtools** | 1.2.4 | 3/6/14 | CRAN (R 3.4.0) |
| **rpart** | 4.1-11 | 3/13/17 | CRAN (R 3.4.2) |
| **rprojroot** | 1.2 | 1/16/17 | CRAN (R 3.4.0) |
| **Rsamtools** | 1.30.0 | 10/31/17 | Bioconductor |
| **RSQLite** | 2 | 6/19/17 | CRAN (R 3.4.1) |
| **rtracklayer** | 1.38.0 | 10/31/17 | Bioconductor |
| **rvest** | 0.3.2 | 6/17/16 | CRAN (R 3.4.0) |
| **S4Vectors** | 0.16.0 | 10/31/17 | Bioconductor |
| **scales** | 0.5.0 | 8/24/17 | CRAN (R 3.4.1) |
| **shape** | 1.4.3 | 8/16/17 | CRAN (R 3.4.1) |
| **shiny** | 1.0.5 | 8/23/17 | CRAN (R 3.4.1) |
| **siggenes** | 1.52.0 | 10/31/17 | Bioconductor |
| **splines** | 3.4.2 | 10/4/17 | local |
| **stats** | 3.4.2 | 10/4/17 | local |
| **stats4** | 3.4.2 | 10/4/17 | local |
| **stringi** | 1.1.6 | 11/17/17 | CRAN (R 3.4.2) |
| **stringr** | 1.2.0 | 2/18/17 | CRAN (R 3.4.0) |
| **SummarizedExperiment** | 1.8.0 | 10/31/17 | Bioconductor |
| **survival** | 2.41-3 | 4/4/17 | CRAN (R 3.4.2) |
| **tibble** | 1.3.4 | 8/22/17 | CRAN (R 3.4.1) |
| **tidyr** | 0.7.2 | 10/16/17 | CRAN (R 3.4.2) |
| **tools** | 3.4.2 | 10/4/17 | local |
| **utils** | 3.4.2 | 10/4/17 | local |
| **viridisLite** | 0.2.0 | 3/24/17 | CRAN (R 3.4.0) |
| **webshot** | 0.5.0 | 11/29/17 | CRAN (R 3.4.2) |
| **withr** | 2.1.0 | 11/1/17 | CRAN (R 3.4.2) |
| **XML** | 3.98-1.9 | 6/19/17 | CRAN (R 3.4.1) |
| **xml2** | 1.1.1 | 1/24/17 | CRAN (R 3.4.0) |
| **xtable** | 1.8-2 | 2/5/16 | CRAN (R 3.4.0) |
| **XVector** | 0.18.0 | 10/31/17 | Bioconductor |
| **yaml** | 2.1.14 | 11/12/16 | CRAN (R 3.4.0) |
| **zlibbioc** | 1.24.0 | 10/31/17 | Bioconductor |
